# IBAS: Interaction-bridged association studies discovering novel genes underlying complex traits

**DOI:** 10.1371/journal.pcbi.1014640

**Published:** 2026-08-17

**Authors:** Dinghao Wang, Pathum Kossinna, Karen Ardila, Senitha Kumarapeli, M. Ethan MacDonald, Jingjing Wu, Qingrun Zhang

**Affiliations:** 1 Department of Mathematics & Statistics, University of Calgary, Calgary, Alberta, Canada; 2 Department of Biochemistry & Molecular Biology, University of Calgary, Calgary, Alberta, Canada; 3 Department of Biomedical Engineering, University of Calgary, Calgary, Alberta, Canada; 4 Schulich School of Medicine & Dentistry, University of Western Ontario, London, Ontario, Canada; 5 Alberta Children’s Hospital Research Institute, University of Calgary, Calgary, Alberta, Canada; 6 Hotchkiss Brain Institute, University of Calgary, Calgary, Alberta, Canada; CANADA

## Abstract

Genetic contributions to complex traits are often mediated through coordinated gene–gene interaction networks, yet most existing association frameworks focus on marginal single-gene effects and overlook higher-order dependency structures. Direct modeling of interactions remains challenging due to combinatorial complexity and statistical instability. We introduce Interaction-Bridged Association Study (IBAS), a general framework that incorporates pathway-level interaction patterns into genotype–phenotype association analysis without explicitly enumerating interactions. IBAS leverages transcriptomic reference data to construct low-dimensional representations of pathway activity, which guide SNP-weighting and gene-level association testing within a kernel-based framework. In perturbation-based simulations, IBAS demonstrates improved stability and reproducibility compared to conventional TWAS and gene-based methods, while maintaining well-calibrated Type I error under phenotype permutation. Application to the WTCCC datasets identifies both known and novel genes across multiple complex diseases, including candidates with modest marginal effects missed by standard approaches. These findings are supported by replication in an independent cohort, and analyses across multiple reference tissues revealing both shared and tissue-specific signals. Overall, IBAS provides a statistically robust and computationally tractable framework for incorporating interaction effects into association mapping, extending beyond the single-gene paradigm and enabling more comprehensive characterization of complex trait. IBAS is available on GitHub at: https://github.com/QingrunZhangLab/IBAS

## Author summary

Understanding how genes influence complex diseases remains a major challenge in genetics. While many studies focus on individual genes, biological systems operate through coordinated interactions among groups of genes. However, directly modeling these interactions is difficult due to their complexity and instability. In this study, we introduce Interaction-Bridged Association Studies (IBAS), a new framework that captures gene–gene interaction patterns without explicitly testing every possible interaction. Instead, IBAS uses gene expression data to summarize pathway-level activity and leverages this information to guide genetic association testing. We show that IBAS produces more stable and reproducible results compared to existing methods, particularly when data are noisy or variable. Applying IBAS to multiple disease datasets, we identify both well-known and previously unreported genes, including candidates with subtle effects that are often missed by traditional approaches. Many findings are replicated in independent cohorts and across different tissues, supporting their robustness. Overall, IBAS provides a practical and reliable way to uncover how coordinated gene activity contributes to complex traits, offering new insights into disease mechanisms.

## Introduction

Discovering genetic basis of complex traits is a long-standing theme in genetics and genomics [1]. Towards this end, genotype-phenotype association mapping has emerged as an essential tool over the last 20 years [2,3]. In recent times, while identifying genes statistically associated with diseases remains a primary goal, researchers have gradually shifted their focus to their underlying mechanisms through various means of interpretations [4]. Along this line, the availability of multi-scale -omics data especially transcriptome allows researchers to develop sophisticated statistical methods to improve both the discovery of genes and their interpretations [5–10]. Among many biologically sensible hypothetical mechanisms, it is intuitive to expect that the variation of gene-gene interaction networks would play a critical role mediating genetic effects to phenotype. Methods such as iPath [11] take this premise and study it at the transcriptomic level, investigating perturbations of given pathways at the sample level and the effect on clinical phenotypes. However, despite gene-gene interactions having been studied for a century [12,13], no consensus has been reached in understanding the genetic basis of variation of interactions and its implications to phenotypic changes. In this study, the term gene–gene interaction refers to coordinated transcriptional activity within biological pathways, reflected by structured co-expression or covariance patterns among genes, rather than explicit statistical interaction or genetic epistasis between individual genes or variants. Our objective is therefore not to explicitly model pairwise interaction terms, but to learn low-dimensional representations that summarize these coordinated pathway-level expression programs.

In our view, the lack of statistical genetic models studying the genetic basis of such pathway-level interaction structures is largely due to the astronomical number of potential combinations, which leads to serious statistical instability. Indeed, studying the interaction patterns associated with phenotypes is already a p≫n problem, suffering from statistical instability as well as computational burden [14,15]. By adding an extra layer of the genetic basis of variation of interaction patterns, it only adds substantial risk of instability, potentially leading to many results that may not be robust to perturbation of input data.

Transcriptome-Wide Association Study (TWAS) is a popular model to conduct association mapping utilizing transcriptomes [5,16]. Although it only focuses on single genes, the in-depth characterization of TWAS by our group [17,18] offered a unique insight of developing models incorporating interactions. The mainstream format of TWAS is a two-step protocol: first, one can form an expression prediction model using (usually cis) genotypes in a reference dataset (e.g.,: GTEx [19]). The predicted expression is called Genetically Regulated eXpression, or GReX. Then, in the second step, in the main dataset for association mapping (that does not contain expressions), one can predict expressions using the available genotypes and finally associate the predicted expression to phenotype. It is worth noting that this perspective differs from the conventional objective of TWAS. In mainstream TWAS, gene expression is typically used to construct genetically regulated expression (GReX), and the association between GReX and the phenotype is then tested, often with the goal of identifying causal genes under an instrumental-variable framework. In contrast, the perspective adopted in our previous work and in this study treats gene expression as a data bridge that links genotype information to downstream association testing. Rather than explicitly modeling GReX–phenotype associations, expression-derived information is used to guide SNP-level modeling, including variant selection, weighting, and interaction-aware aggregation. This distinction clarifies that our framework is not a standard TWAS model, but instead an expression-informed variant-set association approach.

Our recent works showed this interpretation of “predicting expressions” was not the essence of TWAS, but rather, misleading. First, theoretical power analysis showed that TWAS could be more powerful than a hypothetical scenario in which expression data is available in the main GWAS dataset [17]. Additionally, TWAS could be underpowered compared to GWAS when the expression heritability is low [17]. Both results question the interpretation of the prediction of expression in TWAS. As such, we proposed to interpret the “prediction” step as a selection of genetic variants directed by expression. From the perspective of Machine Learning, the first step in TWAS is essentially feature selection and the second, feature aggregation. With this interpretation in mind, one may disregard GReX and instead conduct feature selection and aggregation independently. As a result, methodological research focusing on optimal combinations of feature selection and aggregation approaches could be flexibly conducted. Indeed, novel methods splitting these two steps developed by us [18,20] and independently by others [21] showed higher power than standard TWAS.

The above unique insight into TWAS has opened a door of conducting association mapping mediated by any “data-bridge” by replacing the gene expression and its predicted value with another entity for feature selection and a form of feature aggregation [18]. Indeed, another development in our group has provided a useful tool leveraging brain images [22] in a similar manner.

In this work, we designed an unconventional framework by forming a mediator representing interactions computed using gene expressions through statistical learning techniques. We then used it to facilitate the discovery of the genetic basis of variations of interactions and their consequence to phenotypic changes. More specifically, starting from a gene expression matrix of all genes in a prespecified pathway, we extract representative statistics such as PCA [23], t-SNE [24] or UMAP [25] to form the objective function for feature selection. Linear methods such as PCA naturally capture dominant covariance structures, while nonlinear approaches including t-SNE and UMAP can additionally preserve complex local relationships induced by coordinated expression patterns. These learned representations serve as interaction-informed mediators that bridge transcriptomic organization and downstream genotype–phenotype association analysis. Then, following our previous work [18,20,26], a kernel machine [27] is employed for feature aggregation aimed at association test. The outcomes are single genes whose genetic variants alter the variation of gene-gene interactions in the focal pathway, which also impact the phenotypic changes. This method is named “Interaction Bridged Association Study”, or IBAS. Importantly, such representative statistics contributed by many genes in the pathway is more robust to noise than single genes alone, therefore intuitively could be more stable mediators than single genes in a typical TWAS protocol. Indeed, perturbation experiments using GTEx data show that IBAS is more stable than standard TWAS protocols that rely on single genes. We further applied IBAS to the seven diseases of the Wellcome Trust Case Control Consortium, or WTCCC [28] and discovered novel genes underlying pathways and diseases.

## Materials and methods

In this section, we first introduce the details of IBAS implementation, followed by the descriptions of methods used to compare to IBAS. Then the methods to generate perturbation expression data to assess stability are presented. Finally, the data sources are described.

### The IBAS Framework

**Fig 1:**
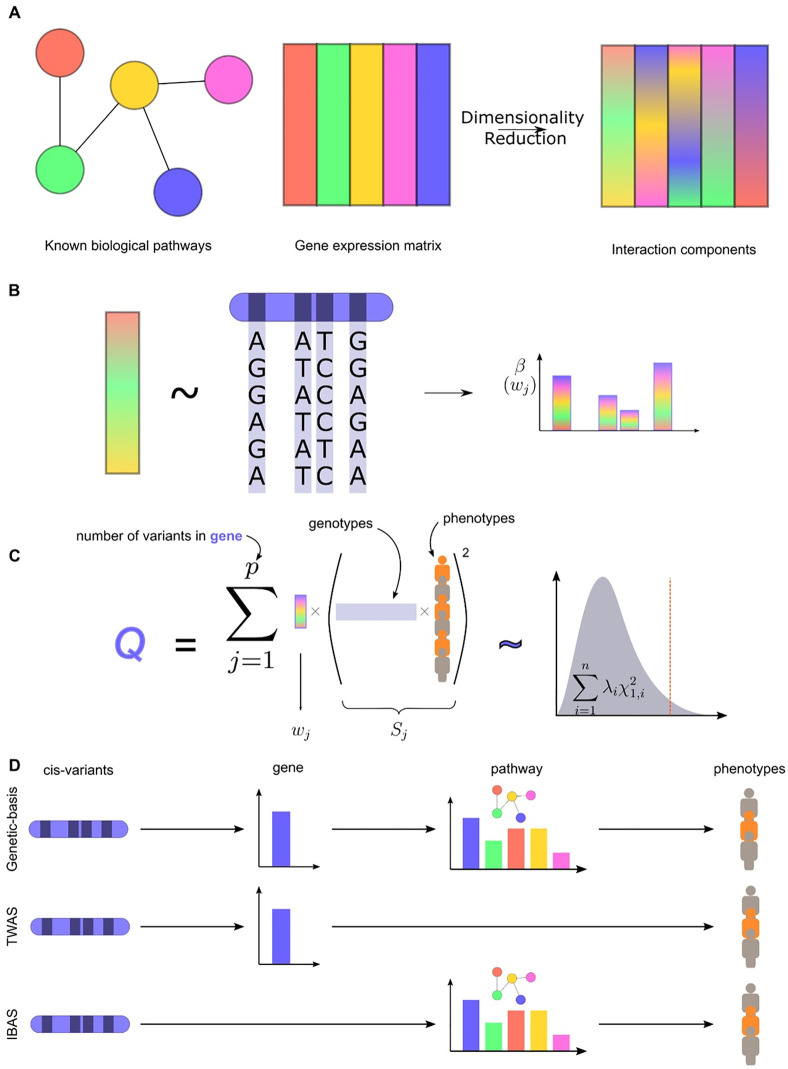
Overview of IBAS Framework. **A)** Gene expression matrices of a reference dataset (such as GTEx) are subset for known biological pathways (from sources such as KEGG, GO, etc.) and undergo dimensionality reduction (using methods such as t-SNE and UMAP) to produce interaction components (“data-bridges”). **B)** Individual interaction components are then associated with *cis*-variants of the genes in the pathway. The coefficients (or significance) of association are interpreted as the contribution of the variants towards the interaction effects at the transcriptome level. **C)** Genotypes in a GWAS dataset are then tested for association with a phenotype of interest, aggregated at the gene level using the Sequence Kernel Association Test, weighted by the interaction contributions obtained in **(B)**. **D)** The natural cascade of genetic effects affecting phenotype are modelled to various extents by existing methods. TWAS methods capture the effect of variants on individual genes’ expression and ignore the contributions of interactions at the pathway level on phenotype while IBAS captures the effect of variants on these interactions (where the individual gene contribution may be also present).

**Step 1** of IBAS utilizes a reference dataset where gene expression and genotype data are available. Known biological pathways (or even user specified collection of genes known to contain interactions) are then used to extract the expression data. The extracted subset of gene expressions (corresponding to a particular pathway) then undergoes dimensionality reduction (using methods such as PCA, t-SNE or UMAP) to produce interaction components (“data-bridges”) **(**Fig 1A). These components are expected to capture interactions at the transcriptome level. In **Step 2**, each component is then associated with individual *cis*-variants corresponding to the genes in the pathway (Fig 1B). The coefficients obtained for each variant indicate their contribution to the interactions captured by the corresponding interaction component. **Step 3** of IBAS involves a GWAS dataset where genotype data is assessed together with a phenotype of interest. Variants in the GWAS dataset are then aggregated to the gene level, weighted by their contribution to interactions (obtained in Step 2 depicted in Fig 1B) and tested for association with phenotypes using the Sequence Kernel Association Test, or SKAT [27] (Fig 1C). This results in the identification of genes associated with the phenotype of interest through the mediation of the pathway being considered.

The natural cascade of genetic variants affecting phenotype may be depicted in Fig 1D**, upper panel**: from variants to gene expression, to pathways and finally phenotype. TWAS captures the effects of variants on gene expression, and then gene expression on phenotype, ignoring the contribution of interactions at the pathway level (Fig 1D**, middle panel**). IBAS on the other hand, captures the effects of cis-variants on the interactions at the pathway level (where it could be argued that the gene-level effect itself is present) and identifies association with the phenotype (Fig 1D**, lower panel**). Therefore, IBAS reveals the genetic basis of complex traits from a compensatory angle.

The implementation of IBAS is structured into the following four components:

**IBAS Implementation (I): Biological pathways**. Biological pathways refer to known interactions among genes, proteins and metabolites [29]. These interactions are often curated from literature by various projects such as Kyoto Encyclopedia of Genes and Genomes (KEGG) [30–32], Reactome [33] or WikiPathways [34]. These databases provide access to both downloading and visualizing these pathways and their known interactions. This work utilizes the KEGG database through the use of the KEGGREST [35] R package. However, IBAS could be applied in a similar manner to any of the pathway databases or even other databases containing known biological interactions such as the Gene Ontology Resource [36].

**IBAS Implementation (II): Dimensionality Reduction**. The downloaded pathways provide context for sub-setting the reference data, but we must turn to dimensionality reduction to uncover the complex interactions within the pathways. Here, we take advantage of three methods often used in the analysis of genomics data: PCA [23], t-distributed Stochastic Neighbour Embedding (t-SNE) [24] and Uniform Manifold Approximation and Projection (UMAP) [25]. These methods are based on the concept of identifying a low-dimensional representation of points in a high-dimensional space.

*PCA* utilizes the principle of iteratively identifying the combinations of components of the higher-dimensional space that maximize captured variance. These components are identified in such a way that they are uncorrelated with all previous PCs resulting in orthogonal combinations of the original data [37]. These components have been identified in various fields to capture variance and identify differences in subpopulations of the data [38,39]. *t-SNE* was originally designed for the purpose of visualizing high-dimensional data in a low-dimensional space (typically 2-dimensional). t-SNE is now often used in the visualization of transcriptomic data, particularly in RNA-Seq [40]. t-SNE is based upon the concept of minimizing the probability distribution of points in a low-dimensional space close to their probability distribution in a high-dimensional space. *UMAP* uses concepts similar to t-SNE, to achieve a low-dimensional embedding. The mathematics behind UMAP are fairly complex [25], and at a high-level, it utilizes “fuzzy simplicial complex” which is a type of weighted graph between the points in the high-dimensional space with the edges denoting the likelihood that two points are connected.

In the current implementation, we retain the first 10 principal components (PCs) as the latent representation of pathway-level expression (or all components when the pathway contains fewer than 10 genes). This choice is motivated by the observation that leading PCs capture the dominant correlation structure among genes, while higher-order components tend to reflect noise. Consistent with PCA loading patterns, the first few PCs are typically driven by groups of highly correlated genes with large variance contributions.

PCA in IBAS serves as a representation learning step to extract low-dimensional features that summarize pathway-level interaction structure and informs SNP weight estimation. Therefore, the number of components acts as a tuning parameter: using too few components may fail to capture relevant structure, while too many may introduce noise and dilute the signal. In practice, a moderate number of components (e.g., 10 PCs) provides a stable balance.

For alternative methods, we reduce pathway dimensionality to a maximum of 10 components for UMAP and 2 components for t-SNE, (or all available components when the pathway contains fewer genes than the specified limit). Similar considerations apply to these methods, where parameters (e.g., number of neighbors, minimum distance, and perplexity) control the learned representation and are chosen based on commonly used settings.

**IBAS Implementation (III): Interaction Directed Feature Selection**. Once a pathway has undergone dimensionality reduction, it is then in a form suitable for association analysis to “select” relevant genetic variants (here we reference feature selection with regards to feature prioritization). Using the same reference dataset, cis-variants are tested for association with each individual component of the interaction components. Each component is analyzed individually as it is expected that different components would capture a different set of interactions among the gene expression in the pathway. While any number of methods could be used for this association analysis, we use the linear mixed model [41,42] which accounts for population stratification by the use of a kinship matrix (which is also called a Genomic Relationship Matrix (GRM) in some literature).

For a specific pathway, the linear mixed model is defined as:


$$\begin{array}{c} v=𝐗\beta +u_{g}+\epsilon \end{array}$$ (1)

where $v\in R^{N\times \text{1}}$ denotes a vector of interaction components in IBAS (e.g., one of the leading principal components) derived from the gene expression matrix of a pathway, where N is the sample size. $𝐗\in R^{N\times \left(n_{c}+n_{p}+\text{1}\right)}$ is the design matrix containing the intercept, the variants to be tested, and other covariates, where $n_{p}$ is the number of cis-variants in this pathway, $n_{c}$ is the number of covariates, and the additional 1 corresponds to the intercept. $\beta \in R^{\left(n_{c}+n_{p}+\text{1}\right)\times \text{1}}$ denotes the vector of coefficients corresponding to the features in 𝐗. $u_{g}\sim N(0,\sigma_{g}^{\text{2}}𝐊)$ denotes the random effects, and $\epsilon \sim N(0,\sigma_{e}^{\text{2}}𝐈)$ denotes the residual error. Here 𝐊 denotes the kinship matrix which provides a measure of the degree of relatedness of the individuals in the sample. $\sigma_{g}^{\text{2}}$ and $\sigma_{e}^{\text{2}}$ represent the variance components of the genetic random effects and residual error, respectively, and 𝐈 denotes the identity matrix.

This association is carried out for all cis-variants of the genes in the pathway. While it is possible that trans-variants also do influence the expression of the genes in the pathway, such testing is extremely resource-intensive and would suffer from a large multiple-testing problem [43]. Typically, cis-variants are considered as the variants within 1Mb of the start and end of the gene and we used this definition for our analysis as well.

IBAS Implementation (IV): Genotype-Phenotype Association Test via Feature Aggregation. Finally, gene-level testing is conducted using the Sequence Kernel Association Test (SKAT). This test combines the effect of multiple variants in a kernel-based score test to test phenotypic association of the aggregate:


$$\begin{array}{c} Q=y^{T}{𝐊}_{𝐬}y \end{array}$$ (2)

where $y\in R^{N\times \text{1}}$ is the normalized phenotype vector, and ${𝐊}_{𝐬}\in R^{N\times N}$ is the kernel matrix constructed from the centralized genotype matrix $𝐆\in R^{N\times c}$ in the GWAS dataset, where c is the number of cis-variants of a gene. In the original SKAT framework, the kernel is defined as


$$\begin{array}{c} {𝐊}_{𝐬}=𝐆{𝐖}_{\text{SKAT}}{\;𝐆}^{⊤}, \end{array}$$

and ${𝐖}_{\text{SKAT}}=\text{diag}(w_{\text{1}},\ldots ,w_{c})$ is a diagonal c by c weight matrix. The weights $w_{j}$ are typically assigned based on minor allele frequency using a Beta distribution.

While multiple kernels may be defined for the use in the score test [27], we use the modified kernel previously used in our work [18,20]:


$$\begin{array}{c} {𝐊}_{𝐰}=𝐆{𝐖}_{𝐈\text{BAS}}{\;𝐆}^{⊤} \end{array}$$ (3)

where ${𝐖}_{\text{IBAS}}=\text{diag}(\alpha_{\text{1}},\ldots ,\alpha_{i},\ldots ,\alpha_{c})$. $\alpha_{i}$ here, denotes the coefficients of association derived from Equation 1 for the reference data. These coefficients of association may be selected as the direct coefficients from the linear mixed model (i.e., $\alpha_{i}=\left|\beta_{i}\right|$; coefficient-based weighting) or as the p-values associated with each coefficient from the same (i.e., $\alpha_{i}=-\text{log}p_{i}$; p-value based weighting). This is then used in Equation 2 to calculate Q which follows a mixture of chi-square distributions [44] (Fig 1C).

When a single interaction component (e.g., the first principal component) is used, SNP weights reflect the association between genetic variants and a single dominant interaction pattern within the pathway. However, complex biological interactions are often distributed across multiple components, and relying on a single component may fail to capture the full interaction structure. To address this, we incorporate multiple interaction components and aggregate their corresponding SNP-level weights.

Specifically, for coefficient-based weighting, the meta-weight for SNP i (i =1,2,...,c) is defined as


$$\begin{array}{c} {\tilde{w}}_{i}=\frac{\text{1}}{K}\sum_{k=\text{1}}^{K}{|\beta }_{ik}|, \end{array}$$

where $\beta_{ik}$ is the regression coefficient of SNP iobtained from the k-th interaction component, and K is the number of components (e.g., the top 10 principal components).

For p-value-based weighting, we combine the evidence across components using Fisher’s method:


$$\begin{array}{c} X_{i}=-\text{2}\sum_{k=\text{1}}^{K}\text{log}(p_{ik}), \end{array}$$

where $p_{ik}$ is the p-value corresponding to SNP i for the k-th component. Under the null hypothesis, $X_{i}\sim \chi_{\text{2}K}^{\text{2}}$, from which a combined p-value is obtained and subsequently transformed into SNP weights.

These meta-weights summarize the overall contribution of each SNP to the dominant interaction structures within the pathway. Unless otherwise specified, coefficient-based aggregation is used in this study.

IBAS performs association testing at the gene level. For each gene within a pathway, SNP-level weights derived from pathway-informed interaction components are applied to its cis-variants, and a gene-level association statistic is computed using a weighted SKAT framework. Thus, similar to TWAS, the basic unit of association in IBAS is the gene rather than individual SNPs.

The output of IBAS is therefore a set of genes associated with the phenotype of interest, mediated through pathway-level interaction effects. As each pathway may contain a large number of genes, multiple hypothesis testing correction is performed using the Benjamini–Hochberg procedure [45] to control false discoveries.

### Theoretical and empirical illustration of PCA for interaction structure

To demonstrate that PCA can recover gene–gene interaction structure and its associated co-expression patterns, we design a simulation with two gene-expression scenarios.

Let p=10 denote the number of genes in a pathway and N=100 the number of samples. For each scenario, we generate a gene expression matrix. For each scenario, we generate a gene expression matrix $X\in R^{p\times N}$, where the i-th column $x_{i}\in R^{p}$ represents the expression profile of the p genes in sample i, and


$$\begin{array}{c} x_{i}\sim N(\text{0},\Sigma ),\;i=\text{1},...,N, \end{array}$$

In the independent scenario, we set the covariance matrix to be isotropic, $\Sigma =\Sigma_{\text{0}}=\sigma^{\text{2}}I_{p}$ with $\sigma^{\text{2}}=\text{1}.\text{4}\text{4}$, yielding an expression matrix $X^{\left(\text{0}\right)}$. In the interaction scenario, we instead generate an expression matrix $X^{\left(\text{1}\right)}$ under a structured covariance $\Sigma \;=\Sigma_{\text{1}}$, where correlations among a subset of genes are introduced to simulate gene–gene interactions. Specifically, $\Sigma_{\text{1}}$ contains two disjoint correlated blocks: $\left\{gene_{\text{1}},gene_{\text{2}}\right\}$ share pairwise covariance τ, and $\left\{gene_{\text{3}},gene_{\text{4}},gene_{\text{5}}\right\}$ share the same pairwise covariance τ (with τ=1.152), while all remaining gene pairs are uncorrelated. Each gene has marginal variance $\sigma^{\text{2}}$.

We then perform PCA by eigen decomposing the empirical gene–gene covariance matrix $\hat{\Sigma }=\frac{\text{1}}{N-\text{1}}XX^{T}\in R^{p\times p}$. We let $\Lambda =diag\left(\lambda_{\text{1}}\geq \cdots \geq \lambda_{p}\right)$ to denote the eigenvalues, which quantify the variance explained by each principal component, and let $V=[v_{\text{1}},\ldots ,v_{p}]$ to denote the PC loading vectors.

Under $\Sigma_{\text{1}}$, the two correlated blocks generate inflated leading eigenvalues $\sigma^{\text{2}}+\text{2}\tau$ and $\sigma^{\text{2}}+\tau$ (S1 Notes), implying that for τ>0, variance concentrates along a small number of dominant eigendirections and the explained-variance ratio $\lambda_{\text{1}}/\sum_{j-\text{1}}^{p}\lambda_{j}$ (and similarly for the next PCs). Correspondingly, the leading loading vectors align with the correlated gene modules, assigning large-magnitude weights to genes $\left\{gene_{\text{1}},gene_{\text{2}}\right\}$ and $\left\{gene_{\text{3}},gene_{\text{4}},gene_{\text{5}}\right\}$. In contrast, under $\Sigma_{\text{0}}$ (independence), the empirical loadings fluctuate around zero without a reproducible block structure.

**Fig 2:**
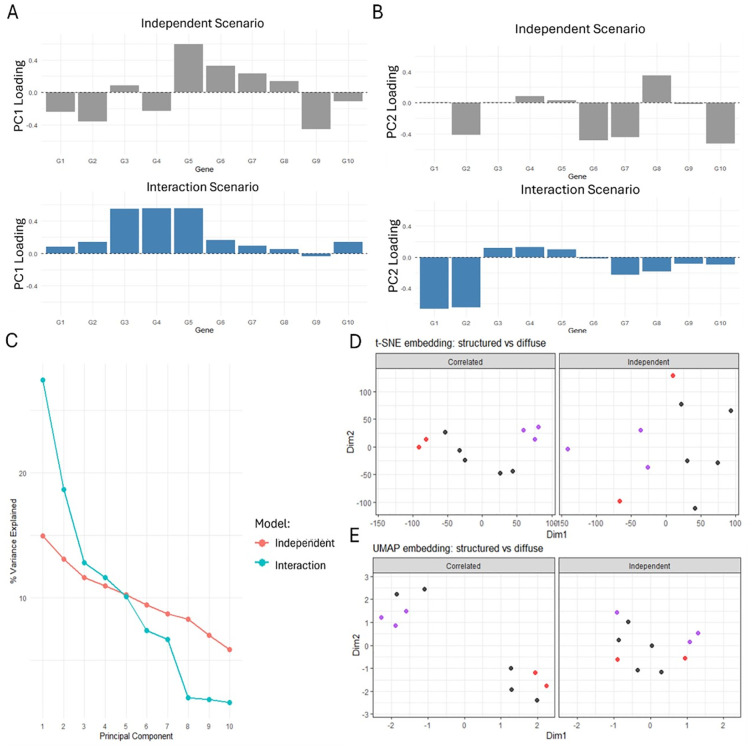
PCA and manifold learning reveal interaction-induced gene modules. **A–B)** PC1 and PC2 loadings for a simulated pathway under independent and interaction (block-correlated) scenarios. Under interaction, loadings reflect module structure ($\left\{gene_{\text{3}},gene_{\text{4}},gene_{\text{5}}\right\}$ for PC1; $\left\{gene_{\text{1}},gene_{\text{2}}\right\}$ for PC2), while under independence they appear unstructured. **C)** Variance explained by PCs, showing stronger concentration in leading components under interaction. **D–E)** t-SNE and UMAP embeddings. Correlated genes cluster by module under interaction (Pink and Yellow) but show no clear grouping under independence.

In our simulation results, PCA clearly distinguishes the interaction and independent scenarios. Under the interaction model, PC1 shows structured loadings with large magnitudes concentrated on genes from the dominant correlated module (Fig 2A), while PC2 captures the remaining module (Fig 2B). Consistent with this behavior, variance is strongly concentrated in the first few principal components in the interaction scenario (Fig 2C), reflecting the emergence of a low-dimensional dependence structure. Theoretically, under the normalized interaction model with within-block correlation ρ=0.8, the leading eigenvalues are expected to be 1+2ρ=2.6 and 1+ρ=1.8 for the second eigenvalue. In our simulation, the corresponding empirical eigenvalues are 2.74 and 1.86, respectively, closely matching these theoretical predictions and confirming that the interaction structure inflates the leading eigendirections as expected. In contrast, under the independent model, both PC1 and PC2 exhibit loadings that fluctuate around zero with no reproducible block pattern across genes (Fig 2A and 2B), and the explained variance is more evenly distributed across components (Fig 2C).

Prior work in data mining has also highlighted PCA as a fundamental tool for detecting correlation structure in high-dimensional data. For example, Böhm et al. [46] note that PCA can “perfectly find” linear correlations between genes, and they build correlation-based clustering methods on top of PCA for applications including gene expression analysis.

Beyond PCA, we also examined whether nonlinear manifold learning methods can recover the same interaction-driven dependence patterns. t-distributed stochastic neighbor embedding (t-SNE) is a nonlinear dimension-reduction method that learns a low-dimensional embedding by preserving local neighborhood structure, typically by minimizing a divergence between neighborhood similarities in the original space and in the embedding, e.g.,


$$\begin{array}{c} \text{min}\;\text{KL}(P∥Q)=\sum_{i\neq j}p_{ij}\;\text{log}\frac{p_{ij}}{q_{ij}}. \end{array}$$

As a result, genes with similar expression profiles tend to be mapped close together. Therefore, when gene–gene interaction structure induces strong within-module correlation, t-SNE tends to place genes from the same correlated module in close proximity, whereas under independence such clustering is not expected. Consequently, when gene–gene interaction structure induces strong within-module correlation, genes in the same module share similar local neighborhoods and are mapped nearby in the t-SNE embedding. In Fig 2D, this is reflected by the tight grouping of $\left\{gene_{\text{1}},gene_{\text{2}}\right\}$ (red) and the clustering of $\left\{gene_{\text{3}},gene_{\text{4}},gene_{\text{5}}\right\}$ (purple) under the interaction scenario, whereas under independence the highlighted genes are intermixed without clear module separation.

Similarly, Uniform manifold approximation and projection (UMAP) is a nonlinear embedding method that builds a weighted k-nearest-neighbor graph to represent local neighborhood structure in the original space and then learns a low-dimensional embedding that best preserves these neighbor relationships by minimizing a cross-entropy objective. A common choice for the embedding-space affinity is


$$\begin{array}{c} {\hat{w}}_{ij}=\frac{\text{1}}{\text{1}+a∥y_{i}-y_{j}{∥}^{\text{2}b}}. \end{array}$$

thus, when gene–gene interaction structure induces strong within-module correlation, genes within the same module tend to share local neighborhoods and are mapped close together, whereas under independence this clustering behavior is not expected. Under the interaction scenario, correlated genes receive consistently higher neighbor weights and form stable local neighborhoods, causing UMAP to place them in close proximity and yield module-level clustering. In contrast, under independence, neighbor relationships are weaker and less structured, producing embeddings with less coherent gene grouping (Fig 2E).

Although the above derivation assumes an idealized equi-covariance block structure, it illustrates a general mechanism: dependence among genes induces dominant eigendirections in the covariance (or correlation) matrix, concentrating variance into a small number of principal components. In more general settings with heterogeneous correlations, PCA no longer admits closed-form eigenpairs, but the leading PCs still recover the dominant low-dimensional dependence patterns. Beyond PCA, nonlinear embedding methods such as t-SNE and UMAP can also reflect gene–gene interaction structure by preserving local neighborhood relationships among genes. When interaction-driven co-expression induces within-module similarity, these methods tend to map genes from the same correlated module to nearby locations in the embedding space, producing module-level clustering patterns.

### Estimation of type I error rates

To assess whether IBAS properly controls false positive rates, we conducted an empirical Type I error evaluation using phenotype permutation. Let $y_{i}\in \{\text{0},\text{1}\}$ denote the binary phenotype for individual i, and let $n_{\text{1}}$and $n_{\text{0}}$ denote the numbers of cases and controls in the original dataset. For each replicate, a null phenotype vector


$$\begin{array}{c} {𝐲}^{\left(b\right)}=(y_{\text{1}}^{\left(b\right)},\ldots ,y_{n}^{\left(b\right)}) \end{array}$$

was generated by randomly permuting the case–control labels while preserving the counts $n_{\text{1}}$and $n_{\text{0}}$. This procedure removes any genuine genotype–phenotype association while retaining the original genotype matrix 𝐆, linkage disequilibrium structure, and gene-level variant composition.

For each permuted phenotype vector, the complete IBAS workflow was applied, including interaction-component construction, variant weighting, and the final gene-level association test using the SKAT statistic Q defined in Equation (2). The empirical Type I error rate was then estimated as


$$\begin{array}{c} \hat{T\text{1}E}=\frac{\text{1}}{N_{g}}\sum_{i}^{N_{g}}1\left(p_{i}<\alpha \right) \end{array}$$

where $p_{i}$ denotes the gene-level p-value obtained under the null phenotype, α is the nominal significance level, and $N_{g}$ is the total number of tested genes.

In addition to this numerical estimate, calibration of the null distribution was visually assessed using quantile–quantile (QQ) plots comparing observed p-values to the theoretical Uniform(0,1) distribution. Agreement between observed and expected quantiles indicates appropriate control of false positives under the null hypothesis.

### Simulation framework for generating perturbed data

To test whether interaction-based protocol is more stable than single-gene based ones, perturbation experiments were conducted to compare them. Simulations were carried out by adding perturbations to gene expression data from the Whole Blood tissue in GTEx [19] to evaluate robustness to changes in reference data. Each expression value was regenerated using a uniform distribution U~(x(1+r), x(1−r)) where x is the current expression value and r={0.1, 0.25, 0.5}. This procedure preserves overall pathway-level expression patterns while introducing gene-level noise, mimicking variability arising from factors such as sampling time and sequencing batch effects. Increasing values of rcorrespond to higher noise levels, and 10 replicates were generated at each level, resulting in 30 perturbed expression datasets.

These datasets were used, together with unperturbed genotype data, to construct data bridges for IBAS under different settings (e.g., dimensionality reduction methods and weighting strategies), as well as to train PrediXcan models. In addition, SKAT-O was applied as a baseline method without expression-derived weighting, and MAGMA was included as a reference approach that does not use SNP-level weights. The resulting models were then evaluated through association testing in the WTCCC cohorts to compare the stability and robustness of results across methods.

### Simulation framework for empirical power evaluation

To complement the perturbation-based stability analysis and empirical Type I error evaluation, we designed a controlled power simulation with predefined causal genes. The simulation was intended to mimic a pathway-mediated disease architecture, where multiple genes within the same coordinated regulatory program jointly contribute to phenotypic variation. In each replicate, causal genes were planted within pathway-level expression structures, and phenotypes were generated under additive, hybrid, and interaction models across multiple heritability levels, allowing us to evaluate the ability of IBAS and competing methods to recover known causal genes under controlled genetic architectures. As in the main IBAS analysis, GTEx expression data were used as the reference transcriptome to derive pathway-level PCA components and estimate SNP weights, while WTCCC genotype data were used as the target dataset from which simulated phenotypes were generated.

Specifically, in each simulation replicate, one pathway was randomly selected, and the genes within that pathway were ranked according to the absolute values of their PC1 loadings derived from the reference expression data. Because PC1 captures the dominant coordinated expression pattern within a pathway, genes with large absolute loadings represent major contributors to this pathway-level regulatory structure. We therefore randomly selected 10 causal genes from the top 50 ranked genes, mimicking a setting in which disease risk is driven by genetic perturbations of genes central to a shared pathway program. Then for each causal gene, five cis-variants were randomly selected. When two genes were assigned as an interaction pair, their selected SNPs were matched one-to-one to form five SNP-pair interaction terms.

For each model, a genotype-derived genetic component g was first constructed from the selected causal variants and then combined with environmental noise to achieve the target heritability. Given a prespecified $h^{\text{2}}$, we generated


$$\begin{array}{c} y=g+\epsilon , \end{array}$$

where $\epsilon \sim N\left(\text{0},\sigma_{\epsilon }^{\text{2}}I\right)$, and $\sigma_{\epsilon }^{\text{2}}$ was chosen such that


$$\begin{array}{c} h^{\text{2}}=\frac{\text{Var}(g)}{\text{Var}(g)+\text{Var}(\epsilon )}. \end{array}$$

We considered four heritability levels: $h^{\text{2}}$ = 0.05, 0.1, 0.4, and 0.7.

To evaluate method performance across different levels of interaction involvement, we used three phenotype-generating architectures. The additive model served as a baseline setting in which phenotypic variation was driven by marginal SNP effects, the interaction model represented an interaction-enriched setting driven by gene-gene interaction terms, and the hybrid model provided an intermediate architecture containing both marginal and interaction-mediated effects.

In the additive model, phenotypic variation was driven by marginal SNP effects across all 10 causal genes:


$$\begin{array}{c} g_{\text{add}}=\sum_{j=\text{1}}^{\text{1}\text{0}}\sum_{k=\text{1}}^{\text{5}}\beta_{jk}x_{jk}, \end{array}$$

where $x_{jk}$ is the genotype vector of the k-th selected SNP in causal gene j, and $\beta_{jk}\sim N(\text{0},\text{1})$ is the corresponding additive effect size.

In the hybrid model, four causal genes contributed additive effects, while the remaining six causal genes were assigned into three interaction pairs,


$$\begin{array}{c} g_{\text{hyb}}=\sum_{j=\text{1}}^{\text{4}}\sum_{k=\text{1}}^{\text{5}}\beta_{jk}x_{jk}+\sum_{(a,b)\in P_{\text{hyb}}}\sum_{k=\text{1}}^{\text{5}}\gamma_{abk}\left(x_{ak}⊙x_{bk}\right). \end{array}$$

Where $P_{\text{hyb}}=\left(G_{\text{5}},G_{\text{6}}\right)\;,\left(G_{\text{7}},G_{\text{8}}\right)\;,\left(G_{\text{9}},G_{\text{1}\text{0}}\right)$.

In the interaction model, the 10 causal genes were assigned into five gene pairs, $P_{\text{int}}=\left(G_{\text{1}},G_{\text{2}}\right)\;,\left(G_{\text{3}},G_{\text{4}}\right)\;,\left(G_{\text{5}},G_{\text{6}}\right)\;,\left(G_{\text{7}},G_{\text{8}}\right)\;,\left(G_{\text{9}},G_{\text{1}\text{0}}\right)$, and phenotypic variation was generated only from cross-gene SNP interaction effects:


$$\begin{array}{c} g_{\text{int}}=\sum_{(a,b)\in P_{\text{int}}}\sum_{k=\text{1}}^{\text{5}}\gamma_{abk}\left(x_{ak}⊙x_{bk}\right)\;, \end{array}$$

where ⊙ denotes elementwise multiplication across individuals, and $\gamma_{abk}$ is the interaction effect size.

For each combination of genetic architecture and heritability level, 500 simulation replicates were generated. We compared IBAS using PCA-derived weights with SKAT-O, PrediXcan, and MAGMA. For each method, gene-level p-values were adjusted using the Benjamini–Hochberg procedure at FDR 0.05, and empirical power was defined as the proportion of planted causal genes declared significant after correction.


$$\begin{array}{c} \text{Power}=\frac{\left|{\hat{G}}_{\text{sig}}\right|}{\left|G_{\text{causal}}\right|}, \end{array}$$

where $G_{\text{causal}}$ is the set of planted causal genes and ${\hat{G}}_{\text{sig}}$ is the set of causal genes significant after BH correction.

### Real data analysis: Reference data

Publicly available, normalized, and filtered expression data from the “Whole Blood” tissue of the GTEx Consortium [19], comprising 670 individuals and 20,315 transcripts, were used as the reference expression dataset. This data was then corrected for PEER factors [47], the top 5 genotype principal components, age and gender as suggested by the GTEx Portal. This expression data was then used for dimensionality reduction by subsetting for individual pathways. For tissue-specific evaluation, GTEx “Brain Cerebellum” and “Pancreas” expression data were processed using the same pipeline as “Whole Blood” and used as alternative reference datasets for IBAS training for Bipolar Disorder and Type 1 Diabetes, respectively.

The GWAS genotype dataset consisted of 838 individuals and 43,066,422 SNPs in the raw VCF data. Since IBAS relies on SNP-level information shared between the GWAS dataset and the reference dataset used for expression modeling, a high degree of overlap in SNP positions is desirable to ensure sufficient variants are available for weighting and association testing. Therefore, imputation was carried out using SHAPEIT4 [48] for phasing and IMPUTE5 [49] for the final imputation based on data from the 1000 Genome Project [50]. This results in 88,863,451 SNPs for the imputed data. The data was then filtered to include only individuals who had expression data available and general QC (MAF<0.05, deviation from HWE $p<{\text{1}\text{0}}^{-\text{8}}$ and relatedness >0.025) was applied, resulting in 6,187,683 SNPs for 553 individuals. This data was converted to the {tped, tfam} file format and JAWAMix5 was used to convert it to hdf5 [51] for out-of-core association analysis with the interaction components obtained from the expression data.

### Real data analysis: GWAS data containing genotype and phenotype

The Wellcome Trust Case Control Consortium [28] contains genotype data for 13,241 cases and 2,938 controls (individual sample sizes are provided in S1 Table) across 7 diseases: bipolar disease (BD), coronary artery disease (CAD), Crohn’s disease (CD), rheumatoid arthritis (RA), type 1 diabetes (T1D), type 2 diabetes (T2D) and hypertension (HT). The original dataset only contained 393,272 variants since this data was obtained using the Affymetrix GeneChip 500K arrays and thus imputation [49] was conducted as in GTEx genotype data to produce 73,162,888 variants. To further evaluate the performance of IBAS in larger cohorts, we additionally analyzed an independent T1D genome-wide association dataset from dbGaP (Accession: phs000911). This dataset comprises 10,791 samples, substantially exceeding the sample size of the WTCCC T1D cohort (4,901 samples). The inclusion of this dataset enables assessment of the reproducibility and scalability of IBAS findings in a higher-powered setting.

## Results

### IBAS is more stable than single gene-mediated TWAS

**Fig 3:**
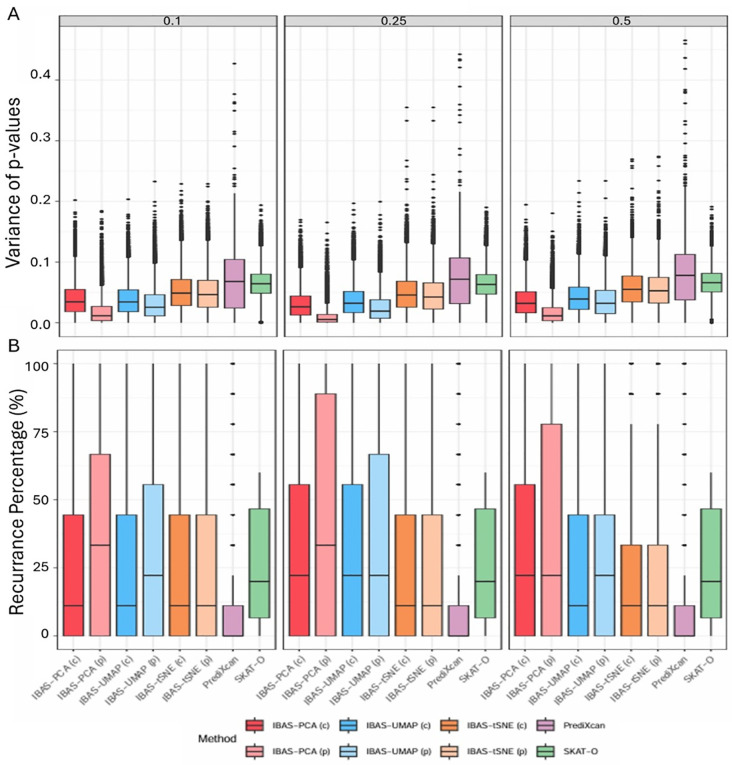
Simulated Perturbations reveal robustness of IBAS to noise in reference data. Simulations were conducted by perturbing gene expression values of each gene by percentage ranges (0.1, 0.25 and 0.5) in the reference data. 10 perturbed expression datasets (replicates) were generated for each of the noise levels and variability of results for case-control association with the Wellcome Trust Case Control Consortium Type 1 Diabetes cohort was evaluated (other cohorts were tested and available in S1-S6 Figs) using IBAS, PrediXcan and SKAT-O. PCA, t-SNE and UMAP dimensionality reduction methods as well as p-value based (p) and coefficient based (c) interaction association weights were tested in the case of IBAS. **A)** Indicates the variance of observed p-values in genes identified as significant (p<0.05) in at least one replicate while **B)** indicates the recurrent significance of genes identified as significant in at least one replicate.

The stability comparison between IBAS and PrediXcan across different reference datasets conveys a consistent and encouraging message. We utilized real data from the GTEx Consortium to construct the data bridge and investigated its effects on the Type 1 Diabetes (T1D) cohort from the WTCCC dataset (Fig 3), along with six additional cohorts presented in S1–S6 Figs to ensure that this stability generalizes across diverse genetic architectures.

All methods considered, including IBAS, perform gene-level association testing using cis-variants within a kernel-based framework. The key distinction lies in how SNP-level weights are constructed. In PrediXcan and kTWAS, weights are derived from gene-specific genotype–expression models, whereas IBAS obtains weights from regression on pathway-level interaction components. Thus, IBAS incorporates pathway-level information while retaining gene-level testing.

To broaden benchmarking beyond TWAS-based approaches, we additionally included the kernel-based gene association method SKAT-O [52] in the perturbation simulations for the T1D cohort. SKAT-O is a widely used gene-level framework that aggregates variant effects without relying on transcriptomic mediators. As an additional reference, we also applied the genotype-based gene analysis method MAGMA [53] to the WTCCC dataset. Because MAGMA does not incorporate gene expression, it is not included in the main figure; its results are instead provided in the S2 Table.

Across all settings, IBAS-based methods showed clear advantages over both PrediXcan and SKAT-O in the stability of discoveries. Fig 3A shows that IBAS achieves substantially lower variance in p-values across a wide range of perturbation levels for genes identified as significant (p < 0.05) in at least one replicate. In particular, PCA-based IBAS demonstrates markedly reduced variability compared with competing methods. Weighting by p-value, rather than by coefficient, further improves stability across all dimensionality reduction approaches.

A similar pattern is observed in the recurrence analysis (Fig 3B), which measures the proportion of genes repeatedly identified as significant across perturbations. PrediXcan exhibits low recurrence, indicating that discoveries are highly sensitive to small changes in the reference data. In contrast, IBAS consistently achieves higher recurrence rates, with PCA-based, p-value–weighted IBAS performing best.

Taken together, these results demonstrate that interaction-based modeling in IBAS yields substantially more robust and reproducible gene-level associations than single gene-mediated TWAS approaches, while also outperforming alternative aggregation methods such as SKAT-O. The MAGMA results provide additional context, confirming that IBAS maintains advantages over conventional GWAS-based gene analysis frameworks.

### Type I Error is under control in IBAS

**Fig 4:**
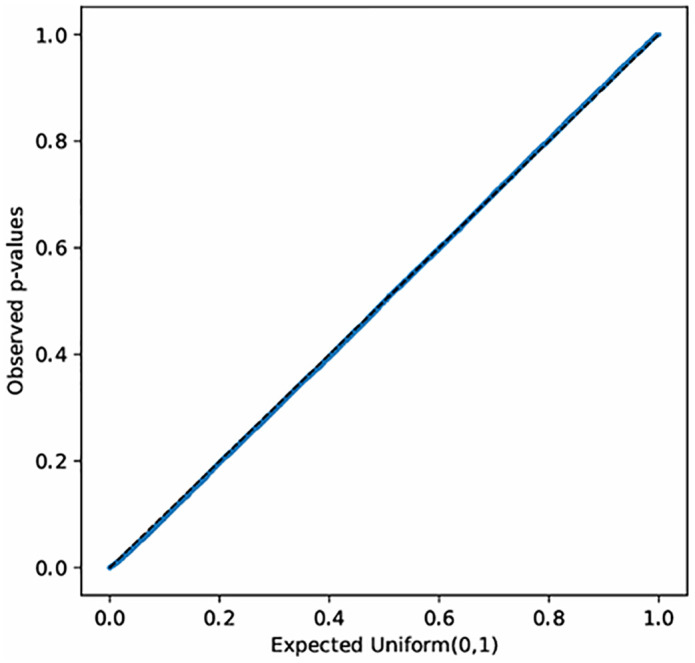
Evaluation of Type I error control in IBAS using QQ plot of null p-values. Quantile–quantile (QQ) plot of gene-level p-values obtained from the IBAS framework under the null hypothesis based on permuted phenotype vectors. The observed p-values closely follow the theoretical 𝐔𝐧𝐢𝐟𝐨𝐫𝐦(0,1) distribution (diagonal line), indicating well-calibrated Type I error and appropriate control of false positives.

To further verify the statistical validity of IBAS, we evaluated its calibration under the null hypothesis using phenotype permutation while preserving the original case–control counts. Across replicated null datasets, the empirical Type I error at the nominal level of 0.05 was estimated to be 0.0524, demonstrating that the IBAS testing procedure maintains appropriate control of false positives and closely matches the expected nominal rate. Quantile–quantile analysis of gene-level p-values additionally showed strong agreement with the theoretical Uniform(0,1) distribution (Fig 4), further confirming that the null distribution is well calibrated and that the observed stability of IBAS is not accompanied by inflation of spurious associations.

### IBAS improves empirical power in simulation studies

**Fig 5:**
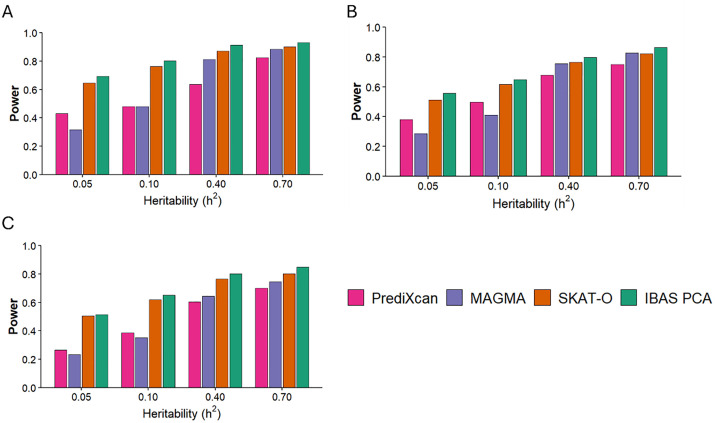
Empirical power evaluation using planted causal gene simulations. Empirical power was evaluated as the proportion of predefined causal genes recovered after Benjamini–Hochberg correction across 500 simulation replicates for each heritability level. IBAS using PCA-derived weights was compared with SKAT-O, PrediXcan, and MAGMA under three phenotype-generating models: **A)** additive model, where causal variants contribute through marginal SNP effects; **B)** hybrid model, where both additive and cross-gene interaction effects contribute to the phenotype; and **C)** interaction-enriched model, where phenotypic variation is driven by cross-gene product terms designed to mimic pathway-level dependency effects. Across the three settings, IBAS showed consistently strong recovery of causal genes, with more pronounced advantages under interaction-enriched architectures.

To further evaluate the performance of IBAS, we assessed empirical power in simulations with predefined causal genes. This analysis tested whether the pathway-level interaction bridge used by IBAS improves the recovery of true genetic signals across different genetic architectures. By comparing additive, hybrid, and interaction architectures, the simulation further assessed whether the advantage of IBAS depends on the extent to which phenotypic variation is mediated through coordinated genetic effects within pathways. Across all three simulation models, IBAS using PCA-derived weights showed stronger recovery of causal genes than SKAT-O, PrediXcan, and MAGMA (Fig 5). IBAS achieved comparable or higher power under the additive model, with increasingly clear advantages under the hybrid and interaction models. These results suggest that the pathway-level interaction representations learned by IBAS provide informative genetic prioritization beyond marginal gene effects or unweighted variant aggregation, enabling more effective recovery of coordinated genetic signals.

Although this simulation provides a simplified representation of complex disease biology, it offers a controlled setting for evaluating the recovery of known causal genes. The higher empirical power of IBAS suggests that pathway-informed interaction weights can improve sensitivity beyond conventional gene-based methods, while the real-data analyses further support its practical utility in identifying biologically meaningful genes and pathways across complex diseases.

### PCA loadings identify biologically coherent gene modules within pathways

To characterize the biological content of the interaction components generated by dimensionality reduction, we analyzed gene loadings within representative pathways and evaluated whether dominant contributors correspond to known functional modules. We found that genes with the largest absolute loadings consistently formed biologically coherent groups aligned with established pathway functions. In each examined case, the leading principal components were driven by coordinated gene modules reflecting structured pathway-level gene activity. These results demonstrate that the low-dimensional interaction representations derived in IBAS capture biologically organized pathway mechanisms.

For a pathway containing p genes, PCA decomposes the covariance matrix of the expression matrix into eigenvalues $\lambda_{\text{1}}\geq \lambda_{\text{2}}\geq \ldots \geq$ $\lambda_{p}$ and corresponding eigenvectors $v_{\text{1}},v_{\text{2}},\ldots ,v_{p}$. The first principal component (PC1), associated with $\lambda_{\text{1}}$, captures the maximal variance within the pathway-level expression space. The entries of $v_{\text{1}}=(v_{\text{1}\text{1}},\ldots ,v_{\text{1}p}{)}^{⊤}$ represent gene loadings, quantifying each gene’s contribution to the dominant interaction axis. Genes were ranked by absolute loadings $|v_{\text{1}j}|$, as magnitude reflects strength of contribution independent of sign. The top five genes were then evaluated for functional consistency with pathway biology.

As an illustrative example, we examined the MAPK signaling pathway (hsa04010), under Environmental Information Processing and Signal Transduction. Ranking genes by absolute loadings of the first principal component identified *HSPA1A*, *HSPA6*, *CSF1*, *HSPA1L*, and *HSPA1B* as the strongest contributors to the dominant variance axis (S3 Table). Four of these genes belong to the HSP70 heat-shock protein family, which are well known to be strongly co-expressed under cellular stress conditions. MAPK cascades regulate stress-responsive transcriptional programs, and coordinated induction of heat-shock genes is a characteristic downstream response. In addition, *CSF1*, a cytokine involved in immune and inflammatory signaling, engages MAPK pathways and participates in coordinated activation programs. The emergence of these genes as top contributors indicates that the principal component captures a structured co-expression pattern consistent with stress and inflammatory signaling within the pathway.

A similar pattern was observed in the Cytokine–cytokine receptor interaction pathway (hsa04060), categorized under Environmental Information Processing and Signaling Molecules and Interaction (S3 Table). The genes with the largest absolute loadings included *CCL4*, *IL10RB*, *TNFRSF1A*, *CCL3*, and *CXCR2*, comprising chemokines and their cognate receptors central to immune signaling. Chemokines such as *CCL3* and *CCL4* are frequently co-induced during inflammatory activation, while receptors including *CXCR2* and *TNFRSF1A* mediate coordinated cytokine responses. The concentration of these immune signaling genes among the top loadings indicates that the principal component reflects a coherent inflammatory signaling program within the pathway, arising from shared covariance structure among functionally related genes.

Together, these examples demonstrate that principal components derived from pathway-level expression matrices capture coordinated gene activity that aligns with established biological functions. Because IBAS leverages these structured interaction representations as mediators in genotype–phenotype association testing, the method effectively links genetically driven variation in coordinated pathway programs to phenotypic outcomes. This provides both statistical stabilization through dimensionality reduction and mechanistic interpretability grounded in known pathway biology.

### IBAS discovers novel genes through the mediation of interactions

**Fig 6:**
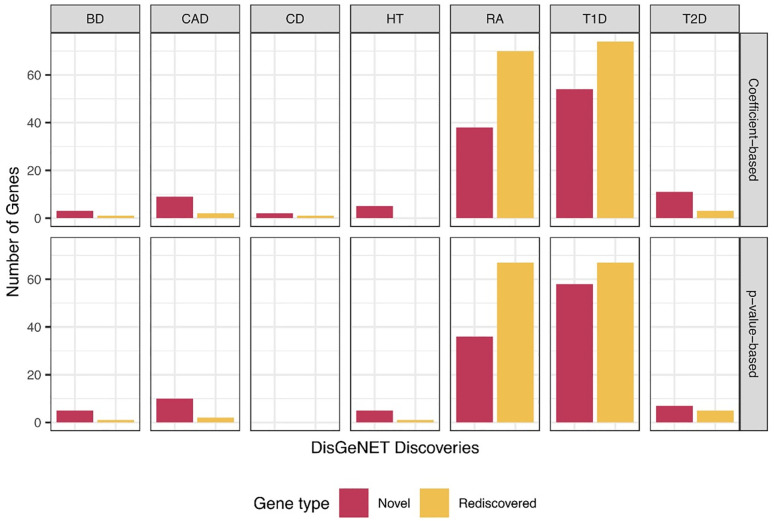
Evaluation of genes associated with disease uncovered by IBAS. Genes identified as associated with each of the 7 diseases (columns) in the WTCCC dataset (with GTEx used as reference) across both coefficient-based and p-value based (rows) for the PCA data-bridge were annotated as “Rediscovered” if found as previously associated with the same disease in the DisGeNET database. Remaining genes are annotated as “Novel”. Evaluation of t-SNE and UMAP can be found in S13-S14 Figs.

IBAS uncovered significant discoveries across the 7 diseases of the WTCCC case-control cohort; a number of which were previously identified in the DisGeNET database [54] with an additional number of discoveries that were novel (Fig 6). Most discoveries were for the Rheumatoid Arthritis (RA) and T1D cohorts both of which also contain the most annotations in the DisGeNET database being autoimmune diseases that have been extensively studied. However, IBAS was also able to uncover significantly associated genes for Bipolar Disorder (BD), Coronary Artery Disease (CAD), Hypertension (HT) and Type-2 Diabetes (T2D) (S4 Table). Furthermore, the discovery of numerous novel genes is expected and welcomed since most association studies incorporate purely genetic information (or, in the case of TWAS, transcriptomic information of a single gene).

**Fig 7:**
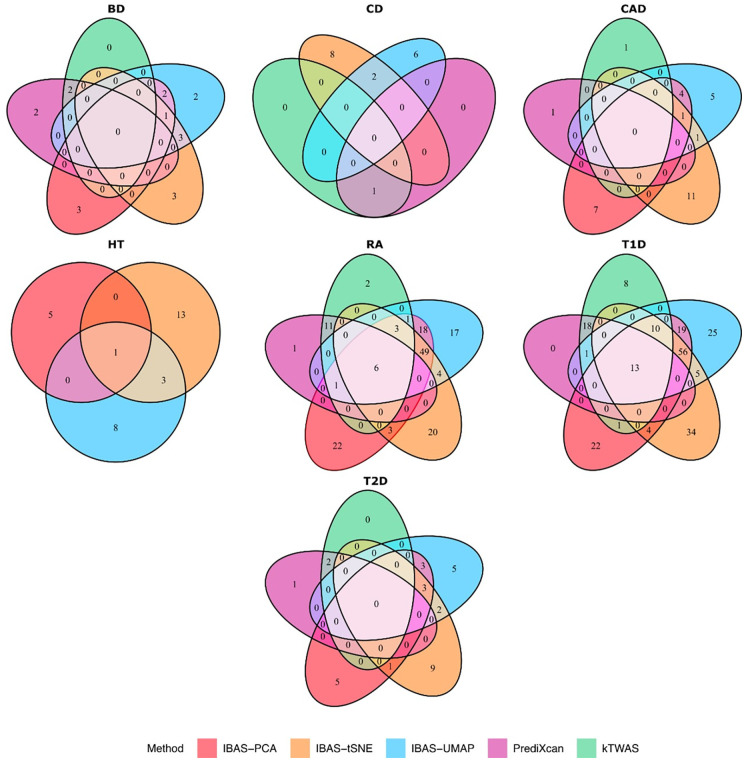
Comparison of IBAS results with kTWAS and PrediXcan. Genes identified by each of the IBAS methods (PCA, t-SNE and UMAP) as well as PrediXcan and kTWAS were compared to identify overlap and uniqueness of discoveries across the 7 diseases of the WTCCC dataset.

IBAS shows contrastive results compared to other gene-disease association tools (Fig 7). PrediXcan [5] and kTWAS [20] are used for this comparison since they represent analyses based on the typical TWAS framework and a kernel-based framework similar to IBAS but without the interaction effects, respectively. Results from IBAS are once again based on the meta-weights (mean for coefficient based and Fisher combined p-values for p-value based across all dimensionalities reduced components).

kTWAS and PrediXcan were found to report fewer results in most diseases, but with a level of overlap between them that suggests that while kTWAS does gain more power due to the use of the kernel-based test for feature aggregation as shown previously [20], they extract similar information from transcriptomic data. IBAS on the other hand, incorporates interactions from gene expression and thus is expected to discover genes based on their association with these interactions. The high number of genes discovered in T1D and RA by both kTWAS and PrediXcan further support the theory previously mentioned that there may be a high marginal effect from genes associated with these diseases. It is also noteworthy that IBAS uncovers several genes associated with HT: one of which, *CLOCK*, has known associations with HT in the DisGeNET database (Fig 6). This further strengthens the hypothesis that IBAS is able to effectively uncover genes that may be associated with disease through their interactions at the pathway level.

### Biological annotation of results

**Fig 8:**
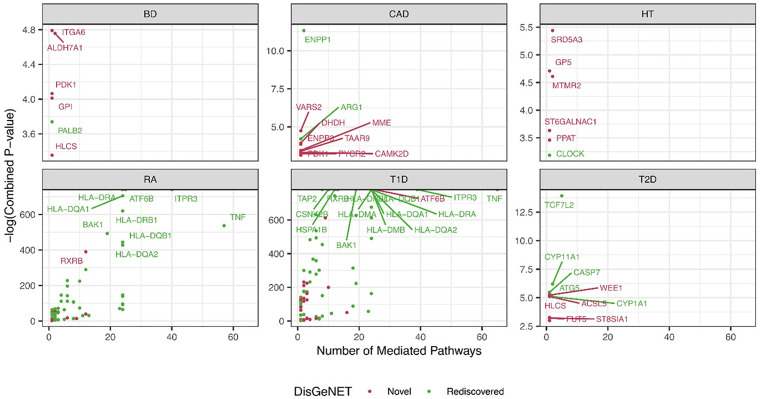
IBAS identifies associations with marginal effects as well as interaction-driven effects. Comparison of significance of previously discovered (“Rediscovered”) and “Novel” genes associated with the 6 disease (no significant genes associated with CD using IBAS-PCA) cohorts of the WTCCC dataset. Combined P-values (y-axis) indicate Fisher combined p-values of genes identified across multiple pathways; while the x-axis denotes the number of pathways mediated through which the gene was identified in as associated with the phenotype.

IBAS distinguishes itself from previous annotations in the WTCCC cohort by identifying genes that may have a small marginal effect but contribute to disease phenotype mediated through relevant pathways (Fig 8). In particular, observing the patterns in diseases where many significant genes are identified such as RA and T1D, previously annotated diseases are identified across multiple pathways – indicating that their strong marginal effect may have contributed to their significance. This could explain the discovery of a large number of genes in the HLA region being identified as significant for these auto-immune disorders [55] as well as *TNF* [55] and *ITPR3* [56]. The weaker marginal effects of genes may explain the lower number of genes identified to be associated with the other diseases since most methods do not account for pathway-level interactions as IBAS does.

IBAS identifies a key mechanism suggesting the mediation of metabolism related pathways and genes in BD. 4 out of the 5 novel genes identified by IBAS are associated with various metabolic processes [57–60]. The role of metabolism in psychiatric disorders has long been investigated [61] with a clear association established between dysfunctional metabolism and psychiatric disorders including BD [62].

*HLCS*, a gene known for its role in biotin metabolism [63] in the body, is found to be highly associated with BD through the biotin metabolism pathway [32,64] by IBAS indicating a potential pathway that could mediate the development of BD. Biotin or Vitamin B7 is known to be vital to brain function as it is a crucial component in the process of glucose metabolism [65]. Furthermore, *HLCS* is highly specific to neurons (both inhibitory and excitatory) in the brain according to the Human Protein Atlas [66,67]. Changes in the excitatory and inhibitory (E/I) synaptic balance have been previously associated with bipolar disorder in animal models [68] and the specificity of HLCS as well as its importance in metabolism make it a potential mediator in the emergence of the BD phenotype. While no studies have been conducted on the direct association of *HLCS* on BD, one study identified increased methylation at CpG sites in *HLCS* in borderline personality disorder patients in their genome-wide association study but failed to find the same in a validation analysis [69]. Increased methylation could indeed be a potential mechanism of repression of *HLCS* expression, resulting in dysfunctional biotin-dependant metabolic pathways which in turn could create an imbalance in the activation of E/I neurons contributing to manic episodes.

In the case of other significant associations, IBAS hints at potential biologically pivotal candidates in disease etiology. *VARS2*, the most significant gene identified with association to CAD (excluding all previously annotated genes in DisGeNET) has been identified to cause heart failure in zebrafish models when knocked-out [70]. Another significant gene, *ENPP3*, has known cardiac-specific function and is downstream of *NKX2–5*, a transcription factor associated with congenital heart disease [71]. *PPAT*, identified to be significant in the HT phenotype, was concluded to be an important gene in the control of BP rhythm in rats [72]. A significant gene (*ACSL5*) identified in T2D, has been studied to be the primary target of the most strongly associated variant of T2D in the *TCF7L2* gene [73], and is important in the metabolism of fatty acids.

**Fig 9:**
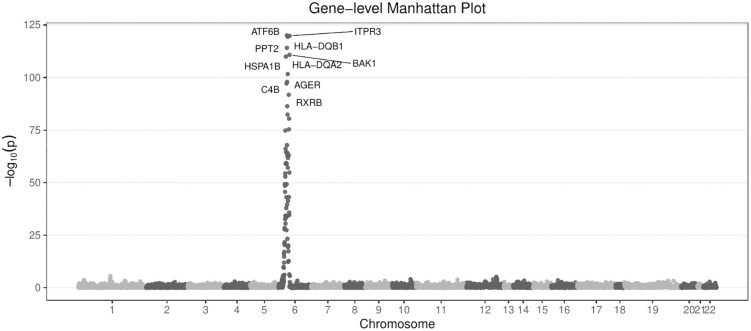
Gene-level Manhattan plot for type 1 diabetes (T1D) in the WTCCC dataset using IBAS. Gene-level Manhattan plot for the WTCCC type 1 diabetes (T1D) dataset using the IBAS framework. Each point represents a gene, plotted by chromosomal position (x-axis) and its association significance measured as $-{\text{log}}_{\text{10}}(\text{p})$ (y-axis). Gene-level statistics were constructed using PCA-based interaction components and coefficient-based variant weighting, with expression weights derived from GTEx Whole Blood tissue. The labeled genes correspond to the top 10 most significant associations.

To further clarify the unit of association in IBAS, we present Manhattan plots of gene-level association results across the genome. Each point represents a gene, with its genomic position defined by gene location and its significance determined by the IBAS gene-level test statistic (Figs 9 and S7–S12).

### Sensitivity to the number of principal components

To evaluate the sensitivity of IBAS to the choice of dimensionality, we repeated the WTCCC T1D analysis using K = 5, 10, and 20 retained pathway components. As expected, the exact composition of the top-ranked genes varied across settings, reflecting the distributed covariance structure within biological pathways. The overlap between the top 50 genes identified using K = 10 and K = 20 was 23 genes (46%), and 14 genes were consistently identified across all three analyses. Importantly, these shared genes included several well-established T1D-associated genes within the major histocompatibility complex (MHC) region, including AGER, C2, HLA-B, HLA-DRA, LTA, TNF, and TNXB. Many of these genes were also prioritized in the independent dbGaP T1D cohort, indicating that the principal biological signals recovered by IBAS are reproducible across datasets and are not driven by a particular choice of dimensionality. These results suggest that the strongest disease-relevant signals, particularly MHC-region T1D genes, are stable across reasonable choices of K, whereas the exact ranking and composition of lower-priority genes remain sensitive to dimensionality. Notably, the MHC region represents the most robust and extensively validated genetic locus for T1D, and the persistence of multiple MHC genes across all K values and across independent cohorts supports the stability of the major biological conclusions derived from IBAS.

### Validation of IBAS discoveries in a larger independent T1D cohort

To further evaluate whether the discoveries from the WTCCC cohort remain detectable in larger datasets, we applied IBAS to an independent T1D genome-wide association cohort from dbGaP (Accession: phs000911). For comparability with the WTCCC analysis, the same reference data, pathway definitions, and interaction-derived variant weights from GTEx whole blood were used. Genotype preprocessing and quality control followed the same general pipeline as for the WTCCC data.

Applying IBAS to the dbGaP cohort produced a set of significant genes that showed substantial concordance with the WTCCC discoveries. The results reported here are based on the PCA IBAS framework with coefficient-based variant weighting, consistent with the primary configuration used in the WTCCC analysis. Approximately 70% of the genes identified in the WTCCC analysis were also detected in the dbGaP dataset, indicating that the interaction-mediated signals uncovered by IBAS are reproducible across independent cohorts despite differences in sample composition and cohort size (S5 Table). The shared genes are strongly enriched in the extended major histocompatibility complex (MHC) region, including multiple classical HLA genes such as *HLA-DQA1*, *HLA-DQB1*, *HLA-DRB1*, *HLA-A*, *HLA-B*, and *HLA-C*, as well as immune-related genes located in the same region such as *TNF*, *LTA*, *LTB*, *TAP1*, *TAP2*, and *PSMB8*. Additional immune-associated genes, such as *ITPR3*, were also consistently detected across the two cohorts. This pattern is consistent with the well-established role of the MHC region as the dominant genetic contributor to T1D susceptibility.

Importantly, the larger dbGaP cohort also revealed additional genes that were not detected in the WTCCC analysis but have strong biological relevance to T1D. These include several canonical non-HLA T1D susceptibility genes such as *INS*, *IL2RA*, *CTLA4*, and *ERBB3*, which are well known to regulate immune responses and pancreatic β-cell function. The appearance of these established loci in the larger dataset suggests that increasing sample size improves the sensitivity of IBAS to detect genes with moderate effects mediated through interaction patterns. At the same time, the preservation of a substantial subset of WTCCC discoveries demonstrates that IBAS captures stable interaction-mediated genetic signals rather than cohort-specific artifacts.

The partial overlap between cohorts is also expected given the complex genetic architecture of T1D. Association signals are highly concentrated within the extended MHC region, where strong linkage disequilibrium and dense gene clusters can lead to multiple correlated genes appearing significant depending on cohort structure and statistical power. Moreover, because IBAS identifies genes whose variants influence interaction structures within biological pathways, rather than relying solely on marginal single-gene effects, the exact ranking of genes can vary across datasets while still reflecting the same underlying interaction-driven genetic mechanisms.

Together, these results provide additional support that IBAS identifies biologically meaningful genes underlying T1D and that its discoveries remain detectable when applied to larger and independent cohorts.

### Evaluation of tissue-specific transcriptome references in IBAS

To assess whether tissue specificity of the reference transcriptome influences IBAS discoveries, we conducted additional analyses using disease-relevant GTEx tissues as alternative reference datasets. Specifically, GTEx Brain Cerebellum expression data were used to train IBAS for the Bipolar Disorder (BD) cohort, and GTEx Pancreas expression data were used for Type 1 Diabetes (T1D). While the primary analyses employed Whole Blood expression due to its broad regulatory coverage, the IBAS framework is not restricted to a single tissue and can naturally incorporate alternative transcriptomic references when biologically appropriate. Across both diseases, tissue-matched references produced gene sets that partially overlapped with Whole Blood discoveries but also revealed additional biologically interpretable candidates, indicating that the interaction structures captured during dimensionality reduction depend on the biological context encoded in the training transcriptome.

In the BD analysis, the Brain-derived reference yielded an expanded set of associated genes, several of which have clear functional relevance to neuronal regulatory processes and psychiatric disease biology (S6 Table). These include genes involved in neuronal chromatin regulation (*EHMT2*), synaptic adhesion and structural connectivity (*CTNNA2*), and axon guidance and neural signaling (*EFNA2*), all of which represent plausible mediators of transcriptional programs affecting neural circuitry. The Brain-based analysis also identified multiple immune-related loci, including members of the TNF signaling pathway and the HLA region, consistent with increasing evidence supporting neuroimmune contributions to psychiatric disorders. Additional candidates associated with mitochondrial respiration and neuronal energy metabolism (*NDUFS7*, *ATP6V1G2*) further highlight the importance of energy-demanding neuronal processes. Together, these findings indicate that when IBAS is trained on brain-derived transcriptomic interaction patterns, it preferentially highlights genes involved in neural regulatory, immune, and metabolic systems that are biologically consistent with the central nervous system context of BD.

To evaluate whether this tissue-dependent pattern extends beyond psychiatric disease, we performed a parallel analysis for T1D using pancreas-derived transcriptomic data. The pancreas-trained model again retained core immune components detectable from Whole Blood, including genes in the TNF pathway and multiple HLA loci, reflecting the established autoimmune basis of T1D (S7 Table). Beyond these shared immune signals, the pancreas-based reference additionally emphasized regulators of cellular stress and inflammatory signaling such as *ATF6B*, a key mediator of the unfolded protein response implicated in endoplasmic reticulum stress, and *ADAM17*, an important modulator of cytokine activation and inflammatory signaling. Additional candidates including *AGER*, an inflammatory receptor associated with metabolic and immune dysfunction, further suggest that pancreas-derived interaction structures capture regulatory programs related to β-cell stress and immune-mediated damage.

Importantly, these observations do not suggest that Whole Blood references are inappropriate; rather, they demonstrate that different tissues encode distinct but complementary interaction-mediated regulatory programs. Because IBAS uses the reference transcriptome primarily to derive pathway-level interaction weights rather than to predict expression directly, the method can flexibly accommodate tissue-specific transcriptomic resources such as those provided by GTEx. This flexibility enables biologically informed analyses tailored to disease context when tissue-matched expression data are available, while still allowing robust discoveries using broadly sampled tissues when they provide greater statistical power.

### Computational complexity and runtime evaluation

IBAS introduces additional computational cost due to its dimensionality reduction step, compared to PrediXcan and MAGMA. For a pathway with N samples, g genes, and m variants, PCA scales on the order of $O(Ng^{\text{2}}+g^{\text{3}})$, while t-SNE and UMAP typically scale between O(NlogN) and $O(N^{\text{2}})$, depending on the implementation. The downstream association and aggregation steps scale approximately linearly with the number of variants, i.e., O(Nm). In our experiments, MAGMA required 52 seconds, PrediXcan 221.7 seconds, and IBAS 667 seconds, with the additional runtime of IBAS primarily driven by the dimensionality reduction step (~400 seconds). Although IBAS incurs higher computational cost, this additional step enables the extraction of pathway-level interaction patterns through low-dimensional representations, which are not captured by standard TWAS or GWAS approaches. All methods were executed on the same computational node (80 CPU cores; 4 × Intel Xeon Gold 6148 @ 2.40GHz; 3022 GB RAM), ensuring a fair comparison.

## Discussion

### Dimensionality selection in representation learning

The choice of dimensionality in the representation learning step is an important practical consideration in IBAS. In this work, we adopt a fixed number of leading components (e.g., top 10 PCs), which provides a stable summary of pathway-level interaction structure in our experiments. However, this choice is not unique, and alternative criteria such as cumulative variance explained or data-adaptive selection strategies could also be considered. More broadly, the dimensionality reduction step introduces a tuning parameter that may influence the balance between signal capture and noise incorporation. While our results suggest that IBAS is robust to reasonable choices of dimensionality and to parameter settings in alternative methods such as UMAP and t-SNE, a systematic investigation of optimal representation learning strategies remains an important direction for future work.

### Stability and validations

We expect that the development of IBAS is a significant progress towards the goal of developing statistically stable models involving interactions. This assumption is because IBAS does not aim to explore all combinations of interactions and therefore does not run the risk of overfitting at this stage. Indeed, we have revealed that the performance of IBAS is stable with respect to the alternation of input expression data (Fig 3). Additionally, as t-SNE and UMAP can extract nonlinear components incorporating interactions flexibly, its performance should be also stable to alternations of the prior knowledge of membership of pathways.

More rigorous validations are needed to quantitatively support the above assumptions. First, numerical simulations with known genetic architectures may be conducted to assess the theoretical properties of IBAS. This is not a trivial task as there is very limited literature reporting patterns of gene-gene interactions that involve many genes in a pathway. During the development of IBAS, we have carried out simulations following the three non-linear genetic architectures, i.e., epistatic, compensatory, and heterogenous models. These non-linear models are better accepted by the field and have been used in the previous publications in our group [18,20,74]. However, these architectures are designed to mimic interactions of two genes and are therefore not suitable for the purpose of verifying IBAS. Instead, we opted to use perturbations of real data in our simulations since there was a lack of literature and benchmarking surrounding the simulation of realistic pathway-level interactions.

### Association v.s. Causality

It is worth noting that, although IBAS used PCA/t-SNE/UMAP represented gene-gene interactions to mediate the association test, we are not claiming the causality of “genetics ⇒ interaction patterns ⇒ phenotype”. Instead, there could be all kinds of causality models, for instance, the pleiotropic model in which genetics causes changes in both interaction patterns and phenotype. Here, the key insight is that we used the interaction patterns to methodologically mediate the discovery, despite the real biological causality relationship is unknown. The same issue applies to many existing works leveraging multi-scale omics to identify association between genetics and phenotype [5–10,18,20,75].

### Tissue choice influences IBAS discoveries while providing complementary biological insights

A critical consideration in the IBAS framework is the choice of reference tissue used to construct the interaction bridge. Our primary discovery analyses relied on Whole Blood transcriptomes, which provide large sample sizes and broad regulatory coverage across common biological pathways. Nevertheless, many complex diseases are expected to involve interaction networks that are partly tissue-specific, particularly in organs directly related to disease pathology. To evaluate this possibility, we performed comparative analyses using disease-relevant GTEx tissues as alternative reference transcriptomes. For Bipolar Disorder, training IBAS on Brain Cerebellum expression data revealed additional genes with clear roles in neuronal regulation and circuitry, including *EHMT2* and *CTNNA2*, supporting the relevance of neural interaction programs. Similarly, for Type 1 Diabetes, using pancreas-derived transcriptomic data emphasized immune and cellular stress regulators such as members of the *TNF* and *HLA* pathways together with stress-response genes including *ATF6B* and inflammatory mediators such as *ADAM17*, consistent with autoimmune β-cell damage as a central disease mechanism. Importantly, these observations do not imply that Whole Blood is unsuitable; rather, they indicate that different tissues encode complementary interaction-mediated regulatory structures. This flexibility allows researchers to select reference tissues according to disease biology while still leveraging broadly sampled tissues when they offer greater statistical power, thereby enhancing the practical applicability of IBAS across diverse complex traits.

### Strengths and Limitations of IBAS

IBAS offers several methodological advantages for association studies involving gene–gene interactions. First, by leveraging dimensionality reduction, IBAS captures structured interaction patterns at the pathway level without explicitly enumerating combinatorial interactions, thereby avoiding the severe statistical and computational burden associated with high-order interaction modeling. Second, the use of pathway-informed representation learning enables IBAS to incorporate biologically meaningful correlation structures into SNP weighting, which can improve power compared to methods that rely solely on gene-level or SNP-level signals. Third, the modular design of IBAS allows flexibility in choosing representation learning techniques (e.g., PCA, UMAP, t-SNE) and weighting strategies, making the framework adaptable to different data types and biological contexts.

Despite these advantages, IBAS also has several limitations. The method depends on the quality and relevance of the reference transcriptomic data, including tissue selection and sample size, which may influence the extracted interaction patterns. In addition, the dimensionality reduction step introduces tuning parameters (e.g., number of components or embedding parameters), and while our results suggest robustness to reasonable choices, suboptimal settings may affect performance. Furthermore, IBAS relies on pathway annotations, and incomplete or inaccurate pathway definitions may limit its ability to capture relevant interaction structures. Finally, while IBAS improves stability by summarizing interaction patterns, it does not explicitly model specific interaction pairs, which may limit interpretability at the individual interaction level.

## Supporting information

S1 Fig Simulated perturbations reveal robustness of IBAS to noise in reference data (bipolar disorder, BD). Gene expression values were perturbed at levels of 0.1, 0.25, and 0.5 to generate 10 replicate datasets per noise level. Association variability was evaluated using the WTCCC BD cohort for both IBAS and PrediXcan. IBAS was implemented with PCA, t-SNE, and UMAP dimensionality reduction, combined with p-value-based (p) and coefficient-based (c) weighting. (a) Variance of observed p-values among genes identified as significant (p < 0.05) in at least one replicate. (b) Recurrence of significant genes across replicates.
(DOCX)

S2 Fig Simulated perturbations reveal robustness of IBAS to noise in reference data (coronary artery disease, CAD). (DOCX)

S3 Fig Simulated perturbations reveal robustness of IBAS to noise in reference data (Crohn’s disease, CD). (DOCX)

S4 Fig Simulated perturbations reveal robustness of IBAS to noise in reference data (hypertension, HT). (DOCX)

S5 Fig Simulated perturbations reveal robustness of IBAS to noise in reference data (rheumatoid arthritis, RA). (DOCX)

S6 Fig Simulated perturbations reveal robustness of IBAS to noise in reference data (type 2 diabetes, T2D). (DOCX)

S7 Fig Gene-level Manhattan plot for bipolar disorder (BD) in the WTCCC dataset using IBAS. Each point represents a gene, plotted by chromosomal position (x-axis) and association significance as −log₁₀(p) (y-axis). Gene-level statistics were derived using PCA-based interaction components and coefficient-based variant weighting, with expression weights from GTEx Whole Blood. Labeled genes correspond to the top 10 associations.
(DOCX)

S8 Fig Gene-level Manhattan plot for coronary artery disease (CAD) in the WTCCC dataset using IBAS. (DOCX)

S9 Fig Gene-level Manhattan plot for Crohn’s disease (CD) in the WTCCC dataset using IBAS. (DOCX)

S10 Fig Gene-level Manhattan plot for rheumatoid arthritis (RA) in the WTCCC dataset using IBAS. (DOCX)

S11 Fig Gene-level Manhattan plot for type 2 diabetes (T2D) in the WTCCC dataset using IBAS. (DOCX)

S12 Fig Gene-level Manhattan plot for hypertension (HT) in the WTCCC dataset using IBAS. (DOCX)

S13 Fig Evaluation of genes associated with disease identified by IBAS using UMAP. Genes identified across seven WTCCC diseases (columns) using coefficient-based and p-value-based weighting (rows) were annotated as “Rediscovered” if previously reported in the DisGeNET database, and “Novel” otherwise.
(DOCX)

S14 Fig Evaluation of genes associated with disease identified by IBAS using t-SNE. (DOCX)

S1 Table Sample sizes of WTCCC cohorts. (DOCX)

S2 Table Significant genes identified by MAGMA in the WTCCC type 1 diabetes (T1D) cohort for comparison with IBAS-based results. (CSV)

S3 Table Top genes ranked by absolute PC1 loadings in the MAPK signaling pathway (hsa04010). (CSV)

S4 Table IBAS-identified genes across WTCCC diseases with DisGeNET annotation support. (CSV)

S5 Table IBAS-identified genes in the dbGaP cohort. (CSV)

S6 Table IBAS-identified genes in bipolar disorder (BD) using brain-derived reference weights. (CSV)

S7 Table IBAS-identified genes in type 1 diabetes (T1D) using pancreas-derived reference weights. (CSV)

S1 Notes Spectral justification for PCA-based interaction representation in IBAS (DOCX)
