# Supplementary material for: IBAS: Interaction-bridged association studies discovering novel genes underlying complex traits
Source: PLoS Comput Biol. 2026 Aug 17;22(8):e1014640. doi: 10.1371/journal.pcbi.1014640 (PMC13480648; doi:10.1371/journal.pcbi.1014640)
Supplement: Supplementary file 1 [file pcbi.1014640.s001.docx]

# S1 Fig– Simulated Perturbations reveal robustness of IBAS to noise in reference data (BD).

Simulations were conducted by perturbing gene expression values of each gene by percentage ranges (0.1, 0.25 and 0.5) in the reference data. 10 perturbed expression datasets (replicates) were generated for each of the noise levels and variability of results for case-control association with the Wellcome Trust Case Control Consortium Bipolar Disorder cohort was evaluated using both IBAS and PrediXcan. PCA, t-SNE and UMAP dimensionality reduction methods as well as p-value based (p) and coefficient based (c) interaction association weights were tested in the case of IBAS. **A)** Indicates the variance of observed p-values in genes identified as significant ($p<0.05$) in at least one replicate while **B)** indicates the recurrent significance of genes identified as significant in at least one replicate.
