# Supplementary material for: IBAS: Interaction-bridged association studies discovering novel genes underlying complex traits
Source: PLoS Comput Biol. 2026 Aug 17;22(8):e1014640. doi: 10.1371/journal.pcbi.1014640 (PMC13480648; doi:10.1371/journal.pcbi.1014640)
Supplement: Supplementary file 7 [file pcbi.1014640.s007.docx]

# S7 Fig – Gene-level Manhattan plot for bipolar disorder (BD) in the WTCCC dataset using IBAS.

Gene-level Manhattan plot for the WTCCC bipolar disorder (BD) dataset using the IBAS framework. Each point represents a gene, plotted by chromosomal position (x-axis) and its association significance measured as $-\log_{10} \left( p \right)$ (y-axis). Gene-level statistics were constructed using PCA-based interaction components and coefficient-based variant weighting, with expression weights derived from GTEx Whole Blood tissue. The labeled genes correspond to the top 10 most significant associations.
