# Supplementary material for: IBAS: Interaction-bridged association studies discovering novel genes underlying complex traits
Source: PLoS Comput Biol. 2026 Aug 17;22(8):e1014640. doi: 10.1371/journal.pcbi.1014640 (PMC13480648; doi:10.1371/journal.pcbi.1014640)
Supplement: Supplementary file 8 [file pcbi.1014640.s008.docx]

# S8 Fig – Gene-level Manhattan plot for coronary artery disease (CAD) in the WTCCC dataset using IBAS.

Gene-level Manhattan plot for the WTCCC coronary artery disease (CAD) dataset using the IBAS framework.
