# Supplementary material for: IBAS: Interaction-bridged association studies discovering novel genes underlying complex traits
Source: PLoS Comput Biol. 2026 Aug 17;22(8):e1014640. doi: 10.1371/journal.pcbi.1014640 (PMC13480648; doi:10.1371/journal.pcbi.1014640)
Supplement: Supplementary file 12 [file pcbi.1014640.s012.docx]

# S12 Fig – Gene-level Manhattan plot for hypertension (HT) in the WTCCC dataset using IBAS.

Gene-level Manhattan plot for the WTCCC hypertension (HT) dataset using the IBAS framework.
