# Supplementary material for: IBAS: Interaction-bridged association studies discovering novel genes underlying complex traits
Source: PLoS Comput Biol. 2026 Aug 17;22(8):e1014640. doi: 10.1371/journal.pcbi.1014640 (PMC13480648; doi:10.1371/journal.pcbi.1014640)
Supplement: Supplementary file 13 [file pcbi.1014640.s013.docx]

# S13 Fig – Evaluation of genes associated with disease uncovered by IBAS-UMAP.

Genes identified as associated with each of the 7 diseases (columns) in the WTCCC dataset (with GTEx used as reference) across both coefficient-based and p-value based (rows) for the UMAP data-bridge were annotated as “Rediscovered” if found as previously associated with the same disease in the DisGeNET database. Remaining genes are annotated as “Novel”.
