# Supplementary material for: IBAS: Interaction-bridged association studies discovering novel genes underlying complex traits
Source: PLoS Comput Biol. 2026 Aug 17;22(8):e1014640. doi: 10.1371/journal.pcbi.1014640 (PMC13480648; doi:10.1371/journal.pcbi.1014640)
Supplement: Supplementary file 15 [file pcbi.1014640.s015.docx]

# S1 Table – Sample sizes of WTCCC cohorts.

| **Disease** | **Case** | **Control** |
| --- | --- | --- |
| Bipolar Disorder (BD) | 1868 | 2938 |
| Coronary Artery Disease (CAD) | 1926 | 2938 |
| Crohn’s Disease (CD) | 1748 | 2938 |
| Rheumatoid Arthritis (RA) | 1860 | 2938 |
| Type 1 Diabetes (T1D) | 1963 | 2938 |
| Type 2 Diabetes (T2D) | 1924 | 2938 |
| Hypertension (HT) | 1952 | 2938 |
