# Supplementary material for: IBAS: Interaction-bridged association studies discovering novel genes underlying complex traits
Source: PLoS Comput Biol. 2026 Aug 17;22(8):e1014640. doi: 10.1371/journal.pcbi.1014640 (PMC13480648; doi:10.1371/journal.pcbi.1014640)
Supplement: Supplementary file 16 [file pcbi.1014640.s022.docx]

# S1 Notes – Spectral Properties of Equi-Correlation Covariance Structures

***Eigenstructure of an equi-covariance block***

Under the interaction scenario, consider a correlated block of size $m$ in the population covariance matrix, where each gene pair has covariance $\tau$ (assume $\tau> 0$). The corresponding $m\times m$ covariance submatrix can be written as

$\Sigma_{block}=\left[ \begin{matrix} \begin{matrix} \begin{matrix} \begin{matrix} \sigma^{2} & \tau\end{matrix} \\ \begin{matrix} \tau& \sigma^{2} \end{matrix} \end{matrix} & \begin{matrix} \begin{matrix} \ldots& \tau\end{matrix} \\ \begin{matrix} \ldots& \tau\end{matrix} \end{matrix} \end{matrix} \\ \begin{matrix} \begin{matrix} \begin{matrix} \vdots& \vdots\end{matrix} \\ \begin{matrix} \tau& \tau\end{matrix} \end{matrix} & \begin{matrix} \begin{matrix} \ddots& \vdots\end{matrix} \\ \begin{matrix} \ldots& \sigma^{2} \end{matrix} \end{matrix} \end{matrix} \end{matrix} \right]=\left( \sigma^{2}-\tau\right)I_{m}+{\tau\boldsymbol{1}_{\boldsymbol{m}}\boldsymbol{1}}_{\boldsymbol{m}}^{\boldsymbol{T}}$ ,

where $\boldsymbol{1}_{m}$ denotes the $m$-dimensional all-ones vector.

**Claim A:** $\Sigma_{block}$ has top eigenvector $\boldsymbol{1}_{\boldsymbol{m}}$.

$\Sigma_{block}\boldsymbol{1}_{\boldsymbol{m}}=\left( \sigma^{2}+(m-1) \tau\right)\boldsymbol{1}_{\boldsymbol{m}}$ ,

**Proof.** Use $\mathbf{1}_{m}^{\top}\mathbf{1}_{m}=m$ and $(\mathbf{1}_{m}\mathbf{1}_{m}^{\top})\mathbf{1}_{m}=\mathbf{1}_{m}(\mathbf{1}_{m}^{\top}\mathbf{1}_{m})=m\mathbf{1}_{m}$:

$$\Sigma_{\text{block}}\mathbf{1}_{m}=(\sigma^{2}-\tau)\mathbf{1}_{m}+\tau(\mathbf{1}_{m}\mathbf{1}_{m}^{\top})\mathbf{1}_{m}=(\sigma^{2}-\tau)\mathbf{1}_{m}+\tau m\mathbf{1}_{m}=(\sigma^{2}+(m-1)\tau)\mathbf{1}_{m}.$$

So $\mathbf{1}_{m}$is an eigenvector with eigenvalue $\sigma^{2}+(m-1)\tau$.

**Claim B:** $\Sigma_{block}$ has eigenvector $u$.

For any $w\in\mathbb{R}^{m}$ with $\mathbf{1}_{m}^{\top}u=0$,

$$\Sigma_{\text{block}}u=(\sigma^{2}-\tau)u.$$

**Proof.** If $\mathbf{1}_{m}^{\top}w=0$, then $(\mathbf{1}_{m}\mathbf{1}_{m}^{\top})w=\mathbf{1}_{m}(\mathbf{1}_{m}^{\top}w)=0$. Hence

$$\Sigma_{\text{block}}w=(\sigma^{2}-\tau)w+\tau(\mathbf{1}_{m}\mathbf{1}_{m}^{\top})w=(\sigma^{2}-\tau)w.$$

So $u$ that orthogonal to $\mathbf{1}_{m}$is an eigenvector with eigenvalue $\sigma^{2}-\tau$. This eigenspace has dimension $m-1$.

If $\tau>0$, then

$$\sigma^{2}+(m-1)\tau\text{ }>\text{ }\sigma^{2}-\tau,$$

so the largest eigenvalue of the block is $\sigma^{2}+(m-1)\tau$, and the other eigenvalue $\sigma^{2}-\tau$ has multiplicity $m-1$. (If $\tau<0$, the ordering flips.)

***Extension to block-diagonal covariance matrices***

Since $\Sigma_{1}$ is block-diagonal, the eigenpairs of each block lift to eigenpairs of the full matrix by padding zeros.

Let the first block be $B\in\mathbb{R}^{m\times m}$, also define the eigen vector of $B$ by $w_{1},\ldots,w_{m}$. Let the full $p$-dimensional vectors:

$$u_{i}=(w_{i},0,\ldots,0)^{\top}.$$

First note that $Bw_{i}=\varphi_{i}w_{i}, i = 1,...,m$, so $\varphi_{i}$ are eigenvalues of the block $B$.

Now write a diagonal block $\Sigma$ in form:

$$\Sigma=\left( \begin{matrix} B & 0 \\ 0 & C \end{matrix} \right),$$

where $C$ is the covariance matrix for the rest of genes. Then:

$\Sigma u_{i}=\left( \begin{matrix} B & 0 \\ 0 & C \end{matrix} \right)\left( \begin{aligned} w_{i} \\ 0 \end{aligned} \right)=\left( \begin{aligned} Bw_{i} \\ 0 \end{aligned} \right)=\varphi_{i}\text{ }\left( \begin{aligned} w_{i} \\ 0 \end{aligned} \right)=\varphi_{i}u_{i}$.

So $u_{i}$ is an eigenvector of the full $\Sigma$.

***Eigenstructure of*** $\boldsymbol{\Sigma}_{\boldsymbol{1}}$***with two disjoint correlated blocks***

Similarly, In our case $\Sigma_{1}$has two disjoint correlated blocks: $\{gene_{1},gene_{2}\}$(size $m_{1}=2$) and $\{gene_{3},gene_{4},gene_{5}\}$ (size $m_{2}=3$), and all cross-block covariances are 0. The remaining genes (6–10) are independent with variance $\sigma^{2}$. Then $\Sigma_{1}$is block diagonal, so its eigenvalues are the union of eigenvalues from each diagonal block.

For block $\{gene_{1},gene_{2}\}$: eigenvalues $\sigma^{2}+(2-1)\tau=\sigma^{2}+\tau$ (mult. 1) and $\sigma^{2}-\tau$ (mult. 1).

For block $\{gene_{3},gene_{4},gene_{5}\}$: eigenvalues $\sigma^{2}+(3-1)\tau=\sigma^{2}+2\tau$ (mult. 1) and $\sigma^{2}-\tau$ (mult. 2).

So, the top and second eigen value should be: $\sigma^{2}+2\tau$ and $\sigma^{2}+\tau$, thereby concentrating explained variance in the first few PCs and yielding loading vectors with large magnitude on the correlated genes.

In our interaction scenario we take $\tau>0$, corresponding to positively correlated (co-expressed) genes. When $\tau<0$, the largest eigenvalue is attained in the subspace orthogonal to $\mathbf{1}_{m}$, yielding contrast directions with mixed-sign loadings rather than a single co-expression direction.
