# Supplementary material for: IBAS: Interaction-bridged association studies discovering novel genes underlying complex traits
Source: PLoS Comput Biol. 2026 Aug 17;22(8):e1014640. doi: 10.1371/journal.pcbi.1014640 (PMC13480648; doi:10.1371/journal.pcbi.1014640)
Supplement: Supplementary file 17 [file pcbi.1014640.s024.docx]

**Reviewers' comments:**

We are grateful to the reviewers for their thoughtful and constructive feedback. We have addressed all comments in detail below, and the corresponding revisions have been incorporated into the manuscript and highlighted in yellow.

**Reviewer #1:** Aiming to account for higher-order interactions of genes, the authors proposed IBAS (Interaction-Bridged Association Study), “a general framework that incorporates interaction patterns into association mapping” as a new TWAS method. Numerical comparisons with some existing methods and application to WTCCC GWAS data showed some advantages of the proposed method. The proposed method has potential to be useful in practice.

We sincerely thank the reviewer for the positive evaluation of our work and for recognizing the potential practical utility of the proposed IBAS framework. We are encouraged that the motivation of incorporating higher-order gene–gene interaction patterns into association mapping is viewed as meaningful and relevant.

In response to the reviewer’s comments, we have carefully revised the manuscript to further clarify the methodological framework, improve the presentation of results, and strengthen the biological interpretation of our findings. Detailed responses to each specific comment are provided below.

Main comments.
1. As stated in the abstract, the authors proposed “a general framework that incorporates interaction patterns into association mapping”. More specifically, some components, e.g. PCs, of the expression levels of a set of functionally related genes (taken form GO or KEGG etc) are associated with SNPs; the association parameter estimates are used as weights to form a kernel to test the association between the SNPs and an outcome. My main question is: would the authors show both theoretically and empirically how “interaction patterns” of the genes are in indeed incorporated into the proposed analysis (though for example use of PCs)?

We thank the reviewer for this insightful and important question. We agree that, in the original manuscript, the connection between the low-dimensional representations (e.g., principal components) and the underlying gene–gene interaction structure was not sufficiently clarified.

To address this, we have added a new section entitled “**Theoretical and Empirical Illustration of PCA for Interaction Structure**” (Page 7, Line 291) in the revised manuscript. In this section, we explicitly demonstrate how interaction patterns among genes are captured through dimensionality reduction, both theoretically and empirically.

From a theoretical perspective, we show that when gene expression exhibits structured covariance induced by gene–gene interactions (e.g., correlated gene modules), the covariance matrix admits dominant eigenvalues corresponding to these interaction patterns. The leading principal components therefore align with these correlated gene modules, concentrating variance along low-dimensional directions that reflect pathway-level interaction structure. Additional theoretical details are provided in **Supplementary Note S1**.

Importantly, this interpretation is consistent with prior work. For example, Böhm et al (<https://doi.org/10.1145/1007568.1007620>). note that principal component analysis (PCA) is a standard tool for detecting correlations (i.e., linear dependencies) among features, where dominant components represent underlying correlated structures in the data. In the context of gene expression, such correlations are widely interpreted as coordinated or co-regulated gene activity within pathways.

From an empirical perspective, we conduct simulation studies comparing independent and interaction-driven expression scenarios. The results show that, under interaction structures, principal component loadings concentrate on correlated gene groups and the explained variance is dominated by the leading components, whereas under independence no such structure is observed. These findings are presented in **Figure 2**.

Together, these additions clarify that IBAS does not rely on individual gene expression signals, but instead leverages low-dimensional representations that summarize coordinated gene activity arising from interaction-driven covariance structure. These interaction-informed components are then used to guide SNP weighting and downstream association testing, thereby incorporating interaction patterns into the analysis in a statistically principled manner.

“***Theoretical and Empirical Illustration of PCA for Interaction Structure***

*To demonstrate that PCA can recover gene–gene interaction structure and its associated co-expression patterns, we design a simulation with two gene-expression scenarios.*

*Let* $p=10$ *denote the number of genes in a pathway and* $N=100$ *the number of samples. For each scenario, we generate a gene expression matrix. For each scenario, we generate a gene expression matrix* $X\in\mathbb{R}^{p\times N}$ *, where the* $i$*-th column* $x_{i}\in\mathbb{R}^{p}$ *represents the expression profile of the* $p$ *genes in sample* $i$ *, and*

$x_{i}\mathcal{\sim N}\left( 0,\Sigma\right)$*,* $i=1,...,N$

*In the independent scenario, we set the covariance matrix to be isotropic,* $\Sigma=\Sigma_{0}=\sigma^{2}I_{p}$ *with* $\sigma^{2}=1.44$*, yielding an expression matrix* $X^{\left( 0 \right)}$*. In the interaction scenario, we instead generate an expression matrix* $X^{\left( 1 \right)}$ *under a structured covariance* $\Sigma=\Sigma_{1}$ *, where correlations among a subset of genes are introduced to simulate gene–gene interactions. Specifically,* $\Sigma_{1}$ *contains two disjoint correlated blocks:* $\{gene_{1},gene_{2}\}$ *share pairwise covariance* $\tau$*, and* $\{gene_{3},gene_{4},gene_{5}\}$ *share the same pairwise covariance* $\tau$ *(with* $\tau=1.152$*), while all remaining gene pairs are uncorrelated. Each gene has marginal variance* $\sigma^{2}$*.*

*We then perform PCA by eigen decomposing the empirical gene–gene covariance matrix* $\hat{\Sigma}=\frac{1}{N-1}XX^{T}\in\mathbb{R}^{p\times p}$*. We let* $\Lambda=diag\left( \lambda_{1}\geq\cdots\geq\lambda_{p} \right)$ *to denote the eigenvalues, which quantify the variance explained by each principal component, and let* $V=[v_{1},\ldots,v_{p}]$ *to denote the PC loading vectors.*

*Under* $\Sigma_{1}$*, the two correlated blocks generate inflated leading eigenvalues* $\sigma^{2}+2\tau$ *and* $\sigma^{2}+\tau$ *(****Supplementary Notes S1****), implying that for* $\tau>0$ *, variance concentrates along a small number of dominant eigendirections and the explained-variance ratio* $\lambda_{1}/\sum_{j=1}^{p} \lambda_{j}$ *(and similarly for the next PCs). Correspondingly, the leading loading vectors align with the correlated gene modules, assigning large-magnitude weights to genes* $\{gene_{1},gene_{2}\}$ *and* $\{gene_{3},gene_{4},gene_{5}\}$*. In contrast, under* $\Sigma_{0}$ *(independence), the empirical loadings fluctuate around zero without a reproducible block structure.*

*In our simulation results, PCA clearly distinguishes the interaction and independent scenarios. Under the interaction model, PC1 shows structured loadings with large magnitudes concentrated on genes from the dominant correlated module (****Fig. 2A****), while PC2 captures the remaining module (****Fig. 2B****). Consistent with this behavior, variance is strongly concentrated in the first few principal components in the interaction scenario (****Fig. 2C****), reflecting the emergence of a low-dimensional dependence structure. Theoretically, under the normalized interaction model with within-block correlation* $\rho=0.8$*, the leading eigenvalues are expected to be* $1+2\rho=2.6$ *and* $1+\rho=1.8$ *for the second eigenvalue. In our simulation, the corresponding empirical eigenvalues are* $2.74$ *and* $1.86$*, respectively, closely matching these theoretical predictions and confirming that the interaction structure inflates the leading eigendirections as expected. In contrast, under the independent model, both PC1 and PC2 exhibit loadings that fluctuate around zero with no reproducible block pattern across genes (****Figs. 2A and 2B****), and the explained variance is more evenly distributed across components (****Fig. 2C****).*

*Prior work in data mining has also highlighted PCA as a fundamental tool for detecting correlation structure in high-dimensional data. For example, Böhm et al. [45] note that PCA can “perfectly find” linear correlations between genes, and they build correlation-based clustering methods on top of PCA for applications including gene expression analysis.*

*Beyond PCA, we also examined whether nonlinear manifold learning methods can recover the same interaction-driven dependence patterns. t-distributed stochastic neighbor embedding (t-SNE) is a nonlinear dimension-reduction method that learns a low-dimensional embedding by preserving local neighborhood structure, typically by minimizing a divergence between neighborhood similarities in the original space and in the embedding, e.g.,*

$$minKL(P\parallel Q)=\sum_{i\neq j} p_{ij}log\frac{p_{ij}}{q_{ij}}.$$

*As a result, genes with similar expression profiles tend to be mapped close together. Therefore, when gene–gene interaction structure induces strong within-module correlation, t-SNE tends to place genes from the same correlated module in close proximity, whereas under independence such clustering is not expected. Consequently, when gene–gene interaction structure induces strong within-module correlation, genes in the same module share similar local neighborhoods and are mapped nearby in the t-SNE embedding. In* ***Fig. 2D****, this is reflected by the tight grouping of* $\{gene_{1},gene_{2}\}$ *(red) and the clustering of* $\{gene_{3},gene_{4},gene_{5}\}$ *(purple) under the interaction scenario, whereas under independence the highlighted genes are intermixed without clear module separation.*

*Similarly, Uniform manifold approximation and projection (UMAP) is a nonlinear embedding method that builds a weighted* $k$*-nearest-neighbor graph to represent local neighborhood structure in the original space and then learns a low-dimensional embedding that best preserves these neighbor relationships by minimizing a cross-entropy objective. A common choice for the embedding-space affinity is*

$$\hat{w}_{ij}=\frac{1}{1+a\parallel y_{i}-y_{j}\parallel^{2b}}.$$

*thus, when gene–gene interaction structure induces strong within-module correlation, genes within the same module tend to share local neighborhoods and are mapped close together, whereas under independence this clustering behavior is not expected. Under the interaction scenario, correlated genes receive consistently higher neighbor weights and form stable local neighborhoods, causing UMAP to place them in close proximity and yield module-level clustering. In contrast, under independence, neighbor relationships are weaker and less structured, producing embeddings with less coherent gene grouping (****Fig. 2E****).*

*Although the above derivation assumes an idealized equi-covariance block structure, it illustrates a general mechanism: dependence among genes induces dominant eigendirections in the covariance (or correlation) matrix, concentrating variance into a small number of principal components. In more general settings with heterogeneous correlations, PCA no longer admits closed-form eigenpairs, but the leading PCs still recover the dominant low-dimensional dependence patterns. Beyond PCA, nonlinear embedding methods such as t-SNE and UMAP can also reflect gene–gene interaction structure by preserving local neighborhood relationships among genes. When interaction-driven co-expression induces within-module similarity, these methods tend to map genes from the same correlated module to nearby locations in the embedding space, producing module-level clustering patterns.”*

# ***“******Figure 2 – PCA and manifold learning reveal interaction-induced gene modules.***

***A–B)*** *PC1 and PC2 loadings for a simulated pathway under independent and interaction (block-correlated) scenarios. Under interaction, loadings reflect module structure (*$\{gene_{3},gene_{4},gene_{5}\}$ *for PC1;* $\{gene_{1},gene_{2}\}$ *for PC2), while under independence they appear unstructured.*
***C)*** *Variance explained by PCs, showing stronger concentration in leading components under interaction.*
***D–E)*** *t-SNE and UMAP embeddings. Correlated genes cluster by module under interaction (Pink and Yellow) but show no clear grouping under independence.*”

# ***“******Supplementary Notes S1 – Spectral Properties of Equi-Correlation Covariance Structures***

***Eigenstructure of an equi-covariance block***

*Under the interaction scenario, consider a correlated block of size* $m$ *in the population covariance matrix, where each gene pair has covariance* $\tau$ *(assume* $\tau> 0$*). The corresponding* $m\times m$ *covariance submatrix can be written as*

$\Sigma_{block}=\left[ \begin{matrix} \begin{matrix} \begin{matrix} \begin{matrix} \sigma^{2} & \tau\end{matrix} \\ \begin{matrix} \tau& \sigma^{2} \end{matrix} \end{matrix} & \begin{matrix} \begin{matrix} \ldots& \tau\end{matrix} \\ \begin{matrix} \ldots& \tau\end{matrix} \end{matrix} \end{matrix} \\ \begin{matrix} \begin{matrix} \begin{matrix} \vdots& \vdots\end{matrix} \\ \begin{matrix} \tau& \tau\end{matrix} \end{matrix} & \begin{matrix} \begin{matrix} \ddots& \vdots\end{matrix} \\ \begin{matrix} \ldots& \sigma^{2} \end{matrix} \end{matrix} \end{matrix} \end{matrix} \right]=\left( \sigma^{2}-\tau\right)I_{m}+{\tau\boldsymbol{1}_{\boldsymbol{m}}\boldsymbol{1}}_{\boldsymbol{m}}^{\boldsymbol{T}}$ *,*

*where* $\boldsymbol{1}_{m}$ *denotes the* $m$*-dimensional all-ones vector.*

***Claim A:*** $\Sigma_{block}$ *has top eigenvector* $\boldsymbol{1}_{\boldsymbol{m}}$*.*

$\Sigma_{block}\boldsymbol{1}_{\boldsymbol{m}}=\left( \sigma^{2}+(m-1) \tau\right)\boldsymbol{1}_{\boldsymbol{m}}$ *,*

***Proof.*** *Use* $\boldsymbol{1}_{m}^{\top}\boldsymbol{1}_{m}=m$ *and* $(\boldsymbol{1}_{m}\boldsymbol{1}_{m}^{\top})\boldsymbol{1}_{m}=\boldsymbol{1}_{m}(\boldsymbol{1}_{m}^{\top}\boldsymbol{1}_{m})=m\boldsymbol{1}_{m}$*:*

$$\Sigma_{\text{block}}\boldsymbol{1}_{m}=(\sigma^{2}-\tau)\boldsymbol{1}_{m}+\tau(\boldsymbol{1}_{m}\boldsymbol{1}_{m}^{\top})\boldsymbol{1}_{m}=(\sigma^{2}-\tau)\boldsymbol{1}_{m}+\tau m\boldsymbol{1}_{m}=(\sigma^{2}+(m-1)\tau)\boldsymbol{1}_{m}.$$

*So* $\boldsymbol{1}_{m}$*is an eigenvector with eigenvalue* $\sigma^{2}+(m-1)\tau$*.*

***Claim B:*** $\Sigma_{block}$ *has eigenvector* $u$*.*

*For any* $w\in\mathbb{R}^{m}$ *with* $\boldsymbol{1}_{m}^{\top}u=0$*,*

$$\Sigma_{\text{block}}u=(\sigma^{2}-\tau)u.$$

***Proof.*** *If* $\boldsymbol{1}_{m}^{\top}w=0$*, then* $(\boldsymbol{1}_{m}\boldsymbol{1}_{m}^{\top})w=\boldsymbol{1}_{m}(\boldsymbol{1}_{m}^{\top}w)=0$*. Hence*

$$\Sigma_{\text{block}}w=(\sigma^{2}-\tau)w+\tau(\boldsymbol{1}_{m}\boldsymbol{1}_{m}^{\top})w=(\sigma^{2}-\tau)w.$$

*So* $u$ *that orthogonal to* $\boldsymbol{1}_{m}$*is an eigenvector with eigenvalue* $\sigma^{2}-\tau$*. This eigenspace has dimension* $m-1$*.*

*If* $\tau>0$*, then*

$$\sigma^{2}+(m-1)\tau\text{ }>\text{ }\sigma^{2}-\tau,$$

*so the largest eigenvalue of the block is* $\sigma^{2}+(m-1)\tau$*, and the other eigenvalue* $\sigma^{2}-\tau$ *has multiplicity* $m-1$*. (If* $\tau<0$*, the ordering flips.)*

***Extension to block-diagonal covariance matrices***

*Since* $\Sigma_{1}$ *is block-diagonal, the eigenpairs of each block lift to eigenpairs of the full matrix by padding zeros.*

*Let the first block be* $B\in\mathbb{R}^{m\times m}$*, also define the eigen vector of* $B$ *by* $w_{1},\ldots,w_{m}$*. Let the full* $p$*-dimensional vectors:*

$$u_{i}=(w_{i},0,\ldots,0)^{\top}.$$

*First note that* $Bw_{i}=\varphi_{i}w_{i}, i = 1,...,m$*, so* $\varphi_{i}$ *are eigenvalues of the block* $B$*.*

*Now write a diagonal block* $\Sigma$ *in form:*

$$\Sigma=\left( \begin{matrix} B & 0 \\ 0 & C \end{matrix} \right),$$

*where* $C$ *is the covariance matrix for the rest of genes. Then:*

$\Sigma u_{i}=\left( \begin{matrix} B & 0 \\ 0 & C \end{matrix} \right)\left( \begin{aligned} w_{i} \\ 0 \end{aligned} \right)=\left( \begin{aligned} Bw_{i} \\ 0 \end{aligned} \right)=\varphi_{i}\text{ }\left( \begin{aligned} w_{i} \\ 0 \end{aligned} \right)=\varphi_{i}u_{i}$*.*

*So* $u_{i}$ *is an eigenvector of the full* $\Sigma$*.*

***Eigenstructure of*** $\boldsymbol{\Sigma}_{\boldsymbol{1}}$***with two disjoint correlated blocks***

*Similarly, In our case* $\Sigma_{1}$*has two disjoint correlated blocks:* $\{gene_{1},gene_{2}\}$*(size* $m_{1}=2$*) and* $\{gene_{3},gene_{4},gene_{5}\}$ *(size* $m_{2}=3$*), and all cross-block covariances are 0. The remaining genes (6–10) are independent with variance* $\sigma^{2}$*. Then* $\Sigma_{1}$*is block diagonal, so its eigenvalues are the union of eigenvalues from each diagonal block.*

*For block* $\{gene_{1},gene_{2}\}$*: eigenvalues* $\sigma^{2}+(2-1)\tau=\sigma^{2}+\tau$ *(mult. 1) and* $\sigma^{2}-\tau$ *(mult. 1).*

*For block* $\{gene_{3},gene_{4},gene_{5}\}$*: eigenvalues* $\sigma^{2}+(3-1)\tau=\sigma^{2}+2\tau$ *(mult. 1) and* $\sigma^{2}-\tau$ *(mult. 2).*

*So, the top and second eigen value should be:* $\sigma^{2}+2\tau$ *and* $\sigma^{2}+\tau$*, thereby concentrating explained variance in the first few PCs and yielding loading vectors with large magnitude on the correlated genes.*

*In our interaction scenario we take* $\tau>0$*, corresponding to positively correlated (co-expressed) genes. When* $\tau<0$*, the largest eigenvalue is attained in the subspace orthogonal to* $\boldsymbol{1}_{m}$*, yielding contrast directions with mixed-sign loadings rather than a single co-expression direction.*

*”*

2. The authors' view of TWAS, as demonstrated in a series of their publications, is to use gene expression data to select or weight SNPs, then conduct SNPs-outcome association testing (e.g. by a kernel method). This view is interesting and not wrong, but it is indeed different from the purpose of the mainstream TWAS: either for detecting causal genes to the outcome (in the framework of instrument variable regression) or for associating the genetically regulated gene expression (GReX) with the outcome. It would help the reader if the authors can state this difference clearly.

We thank the reviewer for this insightful comment and fully agree that our perspective differs from the conventional interpretation of TWAS, and that this distinction should be clearly articulated to avoid confusion.

In the revised manuscript, we have explicitly clarified this point in the Introduction (Page 3, line 90).

Specifically, we now distinguish between the mainstream TWAS framework, where gene expression is used to construct genetically regulated expression (GReX) and its association with phenotype is tested, often with a causal interpretation under an instrumental-variable framework, and our perspective, in which gene expression is treated as a data bridge to guide SNP-level modeling.

We emphasize that, in our framework, expression-derived information is used for variant selection, weighting, and interaction-aware aggregation, rather than for explicitly modeling GReX–phenotype associations. In this sense, IBAS extends the TWAS paradigm by leveraging transcriptomic data as a mediator to guide SNP-level modeling, rather than focusing solely on gene-level expression–phenotype associations. This perspective enables a more flexible, expression-informed variant-set association framework that moves beyond the single-gene paradigm while remaining conceptually connected to TWAS.

We believe this revision improves conceptual clarity and helps position IBAS relative to existing TWAS methodologies.

*“It is worth noting that this perspective differs from the conventional objective of TWAS. In mainstream TWAS, gene expression is typically used to construct genetically regulated expression (GReX), and the association between GReX and the phenotype is then tested, often with the goal of identifying causal genes under an instrumental-variable framework. In contrast, the perspective adopted in our previous work and in this study treats gene expression as a data bridge that links genotype information to downstream association testing. Rather than explicitly modeling GReX–phenotype associations, expression-derived information is used to guide SNP-level modeling, including variant selection, weighting, and interaction-aware aggregation. This distinction clarifies that our framework is not a standard TWAS model, but instead an expression-informed variant-set association approach”*

3. Since the old and much smaller WTCCC GWAS data were used in the application while the authors claim that their proposed IBAS is more powerful, could the authors use the results from some new and larger GWAS to demonstrate that indeed some of their unique discoveries are likely true positives?

We thank the reviewer for this important and constructive suggestion. We agree that validation in larger and independent cohorts is critical to support the robustness and credibility of the discoveries made using IBAS.

To address this, we have added a new section entitled “**Validation of IBAS discoveries in a larger independent T1D cohort**” in the revised manuscript (Page 15, line 620). In this analysis, we applied IBAS to an independent genome-wide association dataset from dbGaP (phs000911), which includes 10,791 samples and is substantially larger than the WTCCC T1D cohort (4,901 samples) (Page 11, line 443).

Using the same reference transcriptome, pathway definitions, and interaction-derived SNP weights to ensure comparability, we observed that approximately 70% of the genes identified in the WTCCC analysis were replicated in the larger dbGaP cohort (**Supplementary Table S5**). These shared discoveries are strongly enriched in well-established immune-related loci, particularly within the MHC region, providing evidence that IBAS captures biologically meaningful and reproducible signals.

Importantly, the larger cohort also enabled the identification of additional canonical T1D susceptibility genes, including *INS, IL2RA, CTLA4, and ERBB3*, which were not detected in the WTCCC dataset. These genes are well known to be involved in immune regulation and pancreatic β-cell function, and their emergence in the larger dataset supports the increased sensitivity of IBAS in detecting moderate interaction-mediated effects as sample size increases.

Together, these results demonstrate that IBAS discoveries are not only reproducible across independent datasets but also biologically meaningful, and that the method benefits from increased statistical power in larger cohorts. We believe this additional validation substantially strengthens the empirical support for the proposed framework.

*“To further evaluate the performance of IBAS in larger cohorts, we additionally analyzed an independent T1D genome-wide association dataset from dbGaP (Accession: phs000911). This dataset comprises 10,791 samples, substantially exceeding the sample size of the WTCCC T1D cohort (4,901 samples). The inclusion of this dataset enables assessment of the reproducibility and scalability of IBAS findings in a higher-powered setting.”*

*“****Validation of IBAS discoveries in a larger independent T1D cohort***

*To further evaluate whether the discoveries from the WTCCC cohort remain detectable in larger datasets, we applied IBAS to an independent T1D genome-wide association cohort from dbGaP (Accession: phs000911). For comparability with the WTCCC analysis, the same reference data, pathway definitions, and interaction-derived variant weights from GTEx whole blood were used. Genotype preprocessing and quality control followed the same general pipeline as for the WTCCC data.*

*Applying IBAS to the dbGaP cohort produced a set of significant genes that showed substantial concordance with the WTCCC discoveries. The results reported here are based on the PCA IBAS framework with coefficient-based variant weighting, consistent with the primary configuration used in the WTCCC analysis. Approximately 70% of the genes identified in the WTCCC analysis were also detected in the dbGaP dataset, indicating that the interaction-mediated signals uncovered by IBAS are reproducible across independent cohorts despite differences in sample composition and cohort size (****Supplementary Table S5****). The shared genes are strongly enriched in the extended major histocompatibility complex (MHC) region, including multiple classical HLA genes such as HLA-DQA1, HLA-DQB1, HLA-DRB1, HLA-A, HLA-B, and HLA-C, as well as immune-related genes located in the same region such as TNF, LTA, LTB, TAP1, TAP2, and PSMB8. Additional immune-associated genes, such as ITPR3, were also consistently detected across the two cohorts. This pattern is consistent with the well-established role of the MHC region as the dominant genetic contributor to T1D susceptibility.*

*Importantly, the larger dbGaP cohort also revealed additional genes that were not detected in the WTCCC analysis but have strong biological relevance to T1D. These include several canonical non-HLA T1D susceptibility genes such as INS, IL2RA, CTLA4, and ERBB3, which are well known to regulate immune responses and pancreatic β-cell function. The appearance of these established loci in the larger dataset suggests that increasing sample size improves the sensitivity of IBAS to detect genes with moderate effects mediated through interaction patterns. At the same time, the preservation of a substantial subset of WTCCC discoveries demonstrates that IBAS captures stable interaction-mediated genetic signals rather than cohort-specific artifacts.*

*The partial overlap between cohorts is also expected given the complex genetic architecture of T1D. Association signals are highly concentrated within the extended MHC region, where strong linkage disequilibrium and dense gene clusters can lead to multiple correlated genes appearing significant depending on cohort structure and statistical power. Moreover, because IBAS identifies genes whose variants influence interaction structures within biological pathways, rather than relying solely on marginal single-gene effects, the exact ranking of genes can vary across datasets while still reflecting the same underlying interaction-driven genetic mechanisms.*

*Together, these results provide additional support that IBAS identifies biologically meaningful genes underlying T1D and that its discoveries remain detectable when applied to larger and independent cohorts.”*

Minor comments: Some notations or descriptions are not clear. Here are a few examples:
1. Is the same y used in eq (1) to represent an “interaction component in IBAS” and the outcome trait in eq (2)?

We thank the reviewer for pointing out this ambiguity in notation. We agree that using the same symbol for different quantities may lead to confusion.

In the revised manuscript, we have clarified this by introducing distinct notation for the two quantities. Specifically, in Equation (1), we now use $\boldsymbol{v}$ to denote the interaction component derived from the pathway-level gene expression matrix (Page 5, line 224), while in Equation (2), $\boldsymbol{y}$ is reserved for the normalized phenotype vector used in the gene-level association test (Page 6, line 243).

We have also expanded the corresponding descriptions to explicitly define each variable and its role in the IBAS framework. These changes ensure that the distinction between the interaction-derived mediator and the phenotype is clear and consistent throughout the manuscript.

*“For a specific pathway, the linear mixed model is defined as:*

$\boldsymbol{v}=\boldsymbol{X\beta}+\boldsymbol{u}_{g}+\boldsymbol{\epsilon}$	*(1)*

*where* $\boldsymbol{v}\in\mathbb{R}^{N\times1}$ *denotes a vector of interaction components in IBAS (e.g., one of the leading principal components) derived from the gene expression matrix of a pathway, where* $N$ *is the sample size.”*

## *“This test combines the effect of multiple variants in a kernel-based score test to test phenotypic association of the aggregate:*

$Q=\boldsymbol{y}^{T}\boldsymbol{K}_{\boldsymbol{s}}\boldsymbol{y}$	*(2)*

*where* $\boldsymbol{y}\in\mathbb{R}^{N\times1}$ *is the normalized phenotype vector, and* $\boldsymbol{K}_{\boldsymbol{s}}\in\mathbb{R}^{N\times N}$ *is the kernel matrix constructed from the centralized genotype matrix* $\boldsymbol{G}\in\mathbb{R}^{N\times c}$ *in the GWAS dataset, where* $c$ *is the number of cis-variants of a gene. ”*

2. Eq (2): should y be centered at its sample mean (or generally its mean under H0)?

We thank the reviewer for this helpful comment. In the SKAT framework, the phenotype is modeled under a null model that includes an intercept (and other covariates when applicable), and the score statistic is constructed based on residuals from this null model. As a result, centering of the phenotype is implicitly handled within the SKAT procedure.

To improve clarity, we have revised the manuscript to explicitly state that $\mathbf{y}$represents a normalized phenotype vector in Equation (2) (Page 6, line 243). This clarification ensures consistency with the standard implementation of kernel-based association tests and avoids potential confusion regarding centering.

## *“This test combines the effect of multiple variants in a kernel-based score test to test phenotypic association of the aggregate:*

$Q=\boldsymbol{y}^{T}\boldsymbol{K}_{\boldsymbol{s}}\boldsymbol{y}$	*(2)*

*where* $\boldsymbol{y}\in\mathbb{R}^{N\times1}$ *is the normalized phenotype vector, and* $\boldsymbol{K}_{\boldsymbol{s}}\in\mathbb{R}^{N\times N}$ *is the kernel matrix constructed from the centralized genotype matrix* $\boldsymbol{G}\in\mathbb{R}^{N\times c}$ *in the GWAS dataset, where* $c$ *is the number of cis-variants of a gene. ”*

3. Section “Other Methods compared to IBAS”: are PrediXcan and kTWAS applied to a set of genes (as IBAS), instead of one gene? If so, how? Please write down your models explicitly.

We thank the reviewer for this helpful comment and agree that the distinction in modeling units and implementation should be clarified. To improve clarity and conciseness, the original section “Other Methods compared to IBAS” has been removed, and its key content has been integrated into the revised description below.

In the revised manuscript, we have added explicit clarification at (Page 11, line 460). Specifically, all methods considered, including IBAS, perform gene-level association testing using cis-SNPs. PrediXcan and kTWAS are applied in their standard form, where each gene is modeled independently using its own cis-SNPs, and association with the phenotype is evaluated at the gene level.

We clarify that the key difference between IBAS and these methods lies not in the unit of testing, but in how SNP-level weights are constructed. PrediXcan and kTWAS derive weights from gene-specific genotype–expression models, whereas IBAS derives weights from regression on pathway-level interaction components. These interaction-informed weights are then applied to the cis-SNPs of each gene, followed by gene-level association testing using a kernel-based framework.

This revision makes explicit that IBAS retains gene-level testing comparable to PrediXcan and kTWAS, while incorporating pathway-level interaction information through the weighting scheme.

### *“All methods considered, including IBAS, perform gene-level association testing using cis-SNPs within a kernel-based framework. The key distinction lies in how SNP-level weights are constructed. In PrediXcan and kTWAS, weights are derived from gene-specific genotype–expression models, whereas IBAS obtains weights from regression on pathway-level interaction components. Thus, IBAS incorporates pathway-level information while retaining gene-level testing.*”

**Reviewer #2:** This manuscript introduces the IBAS (Interaction-Bridged Association Study) method as an improvement over traditional TWAS, emphasizing the incorporation of gene interaction patterns into genotype–phenotype association analysis. The overall concept is innovative, but the methodological details, simulation design, and comparative experiments require further refinement. Below are my specific suggestions:

We thank the reviewer for the thoughtful comments and for recognizing the novelty of incorporating gene–gene interaction patterns into association mapping. We also appreciate the suggestions regarding methodological clarity, simulation design, and comparative analyses.

In response, we have revised the manuscript to improve methodological details, strengthen simulations, clarify comparisons, and add additional validation analyses. Detailed responses are provided below.

Major Comments

1. In the Methods section, the authors present several formulas (e.g., X, y, W) but do not specify the dimensionality of each matrix. This omission makes it difficult for readers to follow the correspondence between sample size, gene number, and SNP number. I recommend explicitly annotating dimensions in the formulas to improve clarity.

We thank the reviewer for this helpful suggestion. We agree that explicitly specifying matrix dimensions improves clarity and readability.

In the revised manuscript, we have added dimension annotations for all key variables in the Methods section (Page 5, line 224; Page 6, line 246). In particular, we now explicitly define the dimensions of the response vector, design matrix, coefficient vector, genotype matrix, and kernel matrix in Equations (1) and (2), along with clear descriptions of sample size, number of SNPs, and number of covariates.

These additions clarify the correspondence between model components and data structure, making the formulation easier to follow.

*“For a specific pathway, the linear mixed model is defined as:*

$\boldsymbol{v}=\boldsymbol{X\beta}+\boldsymbol{u}_{g}+\boldsymbol{\epsilon}$	*(1)*

*where* $\boldsymbol{v}\in\mathbb{R}^{N\times1}$ *denotes a vector of interaction components in IBAS (e.g., one of the leading principal components) derived from the gene expression matrix of a pathway, where* $N$ *is the sample size.* $\boldsymbol{X}\in\mathbb{R}^{N\times\left( n_{c}+n_{p}+1 \right)}$ *is the design matrix containing the intercept, the variants to be tested, and other covariates, where* $n_{p}$ *is the number of cis-variants in this pathway,* $n_{c}$ *is the number of covariates, and the additional 1 corresponds to the intercept.* $\boldsymbol{\beta}\in\mathbb{R}^{\left( n_{c}+n_{p}+1 \right)\times1}$ *denotes the vector of coefficients corresponding to the features in* $\boldsymbol{X}$*.* $\boldsymbol{u}_{g}\mathcal{\sim N(}\boldsymbol{0},\sigma_{g}^{2}\boldsymbol{K})$ *denotes the random effects, and* $\boldsymbol{\epsilon}\mathcal{\sim N(}\boldsymbol{0},\sigma_{e}^{2}\boldsymbol{I})$ *denotes the residual error. Here* $\boldsymbol{K}$ *denotes the kinship matrix which provides a measure of the degree of relatedness of the individuals in the sample.* $\sigma_{g}^{2}$ *and* $\sigma_{e}^{2}$ *represent the variance components of the genetic random effects and residual error, respectively, and* $\boldsymbol{I}$ *denotes the identity matrix.*”

## *“Finally, gene-level testing is conducted using the Sequence Kernel Association Test (SKAT). This test combines the effect of multiple variants in a kernel-based score test to test phenotypic association of the aggregate:*

$Q=\boldsymbol{y}^{T}\boldsymbol{K}_{\boldsymbol{s}}\boldsymbol{y}$	*(2)*

*where* $\boldsymbol{y}\in\mathbb{R}^{N\times1}$ *is the normalized phenotype vector, and* $\boldsymbol{K}_{\boldsymbol{s}}\in\mathbb{R}^{N\times N}$ *is the kernel matrix constructed from the centralized genotype matrix* $\boldsymbol{G}\in\mathbb{R}^{N\times c}$ *in the GWAS dataset, where* $c$ *is the number of cis-variants of a gene. In the original SKAT framework, the kernel is defined as*

$$\boldsymbol{K}_{\boldsymbol{s}}=\boldsymbol{G}\boldsymbol{W}_{SKAT}\boldsymbol{G}^{\top},$$

*, and* $\boldsymbol{W}_{SKAT}=diag(w_{1},\ldots,w_{c})$ *is a diagonal* $c$ *by* $c$ *weight matrix.”*

2. In IBAS, each pathway generates a set of SNP weights, but the manuscript does not clearly specify how these weights are integrated into a meta-weight. I recommend presenting explicit formulas for different strategies, such as coefficient-based averaging or p-value based Fisher combination, and evaluating their performance in simulations or real data.

We thank the reviewer for this helpful suggestion. We agree that the aggregation of SNP-level weights across multiple interaction components should be explicitly defined.

In the revised manuscript, we have added explicit formulations for meta-weight construction . Specifically, we now present two strategies: (i) a coefficient-based approach that averages the absolute regression coefficients across components, and (ii) a p-value-based approach that combines evidence using Fisher’s method. These formulations clarify how SNP-level contributions from multiple interaction components are aggregated into a single meta-weight (Page 6, line 262).

We have also expanded the description to explain how these meta-weights are applied to cis-SNPs in gene-level association testing within the SKAT framework, ensuring a clear connection between interaction components, SNP weighting, and downstream testing.

*“When a single interaction component (e.g., the first principal component) is used, SNP weights reflect the association between genetic variants and a single dominant interaction pattern within the pathway. However, complex biological interactions are often distributed across multiple components, and relying on a single component may fail to capture the full interaction structure. To address this, we incorporate multiple interaction components and aggregate their corresponding SNP-level weights.*

*Specifically, for coefficient-based weighting, the meta-weight for SNP* $i$ *(*$i =1,2,...,c$*) is defined as*

$$\tilde{w}_{i}=\frac{1}{K}\sum_{k=1}^{K} {|\beta}_{ik}|,$$

*where* $\beta_{ik}$ *is the regression coefficient of SNP* $i$*obtained from the* $k$*-th interaction component, and* $K$ *is the number of components (e.g., the top 10 principal components).*

*For p-value-based weighting, we combine the evidence across components using Fisher’s method:*

$$X_{i}=-2\sum_{k=1}^{K} log(p_{ik}),$$

*where* $p_{ik}$ *is the p-value corresponding to SNP* $i$ *for the* $k$*-th component. Under the null hypothesis,* $X_{i}\sim\chi_{2K}^{2}$*, from which a combined p-value is obtained and subsequently transformed into SNP weights.*

*These meta-weights summarize the overall contribution of each SNP to the dominant interaction structures within the pathway. Unless otherwise specified, coefficient-based aggregation is used in this study.*

*IBAS performs association testing at the gene level. For each gene within a pathway, SNP-level weights derived from pathway-informed interaction components are applied to its cis-SNPs, and a gene-level association statistic is computed using a weighted SKAT framework. Thus, similar to TWAS, the basic unit of association in IBAS is the gene rather than individual SNPs.*

*The output of IBAS is therefore a set of genes associated with the phenotype of interest, mediated through pathway-level interaction effects. As each pathway may contain a large number of genes, multiple hypothesis testing correction is performed using the Benjamini–Hochberg procedure[45] to control false discoveries.”*

3. The current simulation only perturbs expression matrices under GTEx genotypes but lacks validation of statistical properties. I recommend adding analyses such as:
Checking whether null p-values follow a Uniform[0,1] distribution.
Plotting ROC curves to compare sensitivity and specificity of IBAS vs other methods.
Computing type I error rates to ensure proper error control under the null.

We thank the reviewer for this important suggestion. We agree that evaluating statistical calibration and error control is essential.

In the revised manuscript, we have added a new subsection “**Estimation of Type I Error Rates**” (Page 9, line 374) and corresponding results (Page 12, line 490). Specifically, we conducted phenotype permutation analyses to generate null datasets and applied the full IBAS pipeline to estimate empirical Type I error rates. The estimated Type I error at the nominal level of 0.05 is 0.0524, demonstrating appropriate error control.

In addition, we assessed the null distribution of p-values using quantile–quantile (QQ) plots (**Fig. 4**), which show close agreement with the theoretical Uniform(0,1) distribution. Together, these results confirm that IBAS is well calibrated and does not exhibit inflation of false positives.

While ROC curves are commonly used for classification tasks, association methods are typically evaluated using calibration metrics, particularly Type I error control. Accordingly, we focus on these standard criteria. Simulations further show that IBAS yields more stable p-values and a higher recurrence rate of significant genes under perturbation (**Fig. 3**), indicating that detected signals are more consistent, less sensitive to noise, and more likely to reflect true underlying associations. This improved stability is consistent with greater statistical power. In real data applications, IBAS also identifies a larger set of disease-related genes, further consistent with improved detection ability (**Fig. 6**). This observation aligns with the simulation results, where increased stability and recurrence of significant signals suggest that IBAS is more effective at recovering underlying associations.

*“****Estimation of Type I Error Rates***

*To assess whether IBAS properly controls false positive rates, we conducted an empirical Type I error evaluation using phenotype permutation. Let* $y_{i}\in\{0,1\}$ *denote the binary phenotype for individual* $i$*, and let* $n_{1}$*and* $n_{0}$ *denote the numbers of cases and controls in the original dataset. For each replicate, a null phenotype vector*

$$\boldsymbol{y}^{\left( b \right)}=(y_{1}^{\left( b \right)},\ldots,y_{n}^{\left( b \right)})$$

*was generated by randomly permuting the case–control labels while preserving the counts* $n_{1}$*and* $n_{0}$*. This procedure removes any genuine genotype–phenotype association while retaining the original genotype matrix* $\boldsymbol{G}$*, linkage disequilibrium structure, and gene-level variant composition.*

*For each permuted phenotype vector, the complete IBAS workflow was applied, including interaction-component construction, variant weighting, and the final gene-level association test using the SKAT statistic* $Q$ *defined in Equation (2). The empirical Type I error rate was then estimated as*

$$\hat{T1E}=\frac{1}{N_{g}}\sum_{i}^{N_{g}} \boldsymbol{1}\left( p_{i}<\alpha\right)$$

*where* $p_{i}$ *denotes the gene-level p-value obtained under the null phenotype,* $\alpha$ *is the nominal significance level, and* $N_{g}$ *is the total number of tested genes.*

*In addition to this numerical estimate, calibration of the null distribution was visually assessed using quantile–quantile (QQ) plots comparing observed p-values to the theoretical Uniform*$\left( 0,1 \right)$ *distribution. Agreement between observed and expected quantiles indicates appropriate control of false positives under the null hypothesis.”*

*“****Type I Error Is Under Control in IBAS***

*To further verify the statistical validity of IBAS, we evaluated its calibration under the null hypothesis using phenotype permutation while preserving the original case–control counts. Across replicated null datasets, the empirical Type I error at the nominal level of 0.05 was estimated to be 0.0524, demonstrating that the IBAS testing procedure maintains appropriate control of false positives and closely matches the expected nominal rate. Quantile–quantile analysis of gene-level p-values additionally showed strong agreement with the theoretical Uniform(0,1) distribution (****Fig. 4****), further confirming that the null distribution is well calibrated and that the observed stability of IBAS is not accompanied by inflation of spurious associations.”*

# *“****Figure 4 – Evaluation of Type I error control in IBAS using QQ plot of null p-values.***

*Quantile–quantile (QQ) plot of gene-level p-values obtained from the IBAS framework under the null hypothesis based on permuted phenotype vectors. The observed p-values closely follow the theoretical* $\text{Uniform}\left( 0,1 \right)$ *distribution (diagonal line), indicating well-calibrated Type I error and appropriate control of false positives.”*

4. The manuscript only compares IBAS with PrediXcan, which is insufficient. I recommend including additional benchmarks such as:
S-PrediXcan / MetaXcan: summary-statistics-based TWAS methods.
FUSION: another summary-level TWAS framework.
MAGMA: gene-set analysis method independent of eQTL.
SKAT-O: kernel-based gene-level association test.

We thank the reviewer for this valuable suggestion and agree that broader benchmarking improves the evaluation of IBAS.

In the revised manuscript, we have expanded the comparisons to include both SKAT-O and MAGMA. SKAT-O serves as a widely used kernel-based gene-level association method that does not rely on transcriptomic information, while MAGMA provides a reference framework for genotype-based gene analysis without SNP-level weighting (Page 10, line 406; Page 11, line 466). These additions allow us to evaluate IBAS relative to both expression-informed and expression-independent approaches (**Fig.** **3**).

Regarding S-PrediXcan and FUSION, these methods are designed for summary-statistics-based TWAS analyses, whereas our study is conducted using individual-level genotype data. As such, they are not directly comparable within the current analysis framework.

Our expanded results show that IBAS demonstrates improved stability and reproducibility compared with both PrediXcan and SKAT-O, while maintaining advantages over conventional gene-level approaches such as PrediXcan.

*“These datasets were used, together with unperturbed genotype data, to construct data bridges for IBAS under different settings (e.g., dimensionality reduction methods and weighting strategies), as well as to train PrediXcan models. In addition, SKAT-O was applied as a baseline method without expression-derived weighting, and MAGMA was included as a reference approach that does not use SNP-level weights. The resulting models were then evaluated through association testing in the WTCCC cohorts to compare the stability and robustness of results across methods.”*

*“To broaden benchmarking beyond TWAS-based approaches, we additionally included the kernel-based gene association method SKAT-O in the perturbation simulations for the T1D cohort. SKAT-O is a widely used gene-level framework that aggregates variant effects without relying on transcriptomic mediators. As an additional reference, we also applied the genotype-based gene analysis method MAGMA to the WTCCC dataset. Because MAGMA does not incorporate gene expression, it is not included in the main figure; its results are instead provided in the* ***Supplementary Table S2****.*

*Across all settings, IBAS-based methods showed clear advantages over both PrediXcan and SKAT-O in the stability of discoveries.* ***Fig. 3A*** *shows that IBAS achieves substantially lower variance in p-values across a wide range of perturbation levels for genes identified as significant (p < 0.05) in at least one replicate. In particular, PCA-based IBAS demonstrates markedly reduced variability compared with competing methods. Weighting by p-value, rather than by coefficient, further improves stability across all dimensionality reduction approaches.*

*A similar pattern is observed in the recurrence analysis (****Fig. 3B****), which measures the proportion of genes repeatedly identified as significant across perturbations. PrediXcan exhibits low recurrence, indicating that discoveries are highly sensitive to small changes in the reference data. In contrast, IBAS consistently achieves higher recurrence rates, with PCA-based, p-value–weighted IBAS performing best.*

*Taken together, these results demonstrate that interaction-based modeling in IBAS yields substantially more robust and reproducible gene-level associations than single gene-mediated TWAS approaches, while also outperforming alternative aggregation methods such as SKAT-O. The MAGMA results provide additional context, confirming that IBAS maintains advantages over conventional GWAS-based gene analysis frameworks.”*

***“Revised Figure 3 – Simulated Perturbations reveal robustness of IBAS to noise in reference data.***

*Simulations were conducted by perturbing gene expression values of each gene by percentage ranges (0.1, 0.25 and 0.5) in the reference data. 10 perturbed expression datasets (replicates) were generated for each of the noise levels and variability of results for case-control association with the Wellcome Trust Case Control Consortium Type 1 Diabetes cohort was evaluated (other cohorts were tested and available in* ***Supplementary Figs S1-S6****) using IBAS, PrediXcan and SKAT-O. PCA, t-SNE and UMAP dimensionality reduction methods as well as p-value based (p) and coefficient based (c) interaction association weights were tested in the case of IBAS.* ***A)*** *Indicates the variance of observed p-values in genes identified as significant (*$p<0.05$*) in at least one replicate while* ***B)*** *indicates the recurrent significance of genes identified as significant in at least one replicate.”*

5. In the dimensionality reduction of pathway expression matrices, the manuscript applies PCA but does not specify how many principal components are retained. The authors should provide a clear criterion for PCA dimensionality selection (e.g., cumulative variance explained ≥ 80% or a fixed number of top components) and evaluate the impact of different choices on results. Moreover, the manuscript also mentions alternative methods such as UMAP and t-SNE, which do not rely on variance explained. Therefore, it is even more important to clarify parameter selection strategies (e.g., number of neighbors and minimum distance for UMAP, perplexity for t-SNE) and assess robustness under different parameter settings. This would improve methodological transparency and reproducibility.

We thank the reviewer for this important comment regarding methodological transparency and reproducibility. We agree that the choice of dimensionality and parameter settings should be clearly specified.

In the revised manuscript, we have explicitly described our dimensionality selection strategy. Specifically, we retain the top 10 principal components (or all components when the pathway contains fewer than 10 genes), motivated by the observation that leading components capture the dominant correlation structure while higher-order components tend to reflect noise. We further clarify that this choice represents a practical balance between signal capture and noise reduction, and we explicitly discuss its role as a tuning parameter (Page 5, line 197).

For alternative methods, we now also specify the parameter settings used for UMAP and t-SNE, and note that these are chosen based on commonly used configurations in the literature. We additionally include a dedicated discussion subsection on dimensionality selection to highlight that, while our results indicate stability under reasonable choices, systematic exploration of optimal parameter selection remains an important direction for future work (Page 17, line 725).

While we did not perform an exhaustive sensitivity analysis across all parameter settings, the consistency of results across PCA, UMAP, and t-SNE in our experiments suggests that the IBAS framework is not overly sensitive to the specific representation learning configuration.

*“In the current implementation, we retain the first 10 principal components (PCs) as the latent representation of pathway-level expression (or all components when the pathway contains fewer than 10 genes). This choice is motivated by the observation that leading PCs capture the dominant correlation structure among genes, while higher-order components tend to reflect noise. As shown in the PCA loading analysis, the first few PCs are typically driven by groups of highly correlated genes with large variance contributions.*

*PCA in IBAS serves as a representation learning step to extract low-dimensional features that summarize pathway-level interaction patterns and guide SNP weight estimation. Therefore, the number of components acts as a tuning parameter: using too few components may miss relevant interaction structure, whereas including too many may introduce noise and dilute the signal. In practice, we find that a moderate number of components (e.g., 10 PCs) provides a stable balance.*

*For alternative methods, we reduce pathway dimensionality to a maximum of 10 components for UMAP and 2 components for t-SNE, while using all available components when the pathway contains fewer genes than the specified limit. Similar considerations apply to these methods, where parameters (e.g., number of neighbors, minimum distance, and perplexity) control the learned representation and are chosen based on commonly used settings.”*

## *“****Dimensionality Selection in Representation Learning***

*The choice of dimensionality in the representation learning step is an important practical consideration in IBAS. In this work, we adopt a fixed number of leading components (e.g., top 10 PCs), which provides a stable summary of pathway-level interaction structure in our experiments. However, this choice is not unique, and alternative criteria such as cumulative variance explained or data-adaptive selection strategies could also be considered. More broadly, the dimensionality reduction step introduces a tuning parameter that may influence the balance between signal capture and noise incorporation. While our results suggest that IBAS is robust to reasonable choices of dimensionality and to parameter settings in alternative methods such as UMAP and t-SNE, a systematic investigation of optimal representation learning strategies remains an important direction for future work.”*

6. The manuscript consistently uses blood eQTL across all diseases, but certain diseases (e.g., Crohn’s disease, Rheumatoid arthritis, Type 1 diabetes) may require tissue-specific eQTL (e.g., gut, synovium, pancreas). The authors should justify tissue choice and, where possible, incorporate GTEx tissue-specific data.

We thank the reviewer for this important suggestion and agree that tissue specificity is an important consideration in transcriptome-informed association analyses.

In the revised manuscript, we have added a new section entitled “**Evaluation of tissue-specific transcriptome references in IBAS**” (Page 15, line 661). In this analysis, we incorporated disease-relevant GTEx tissues, including Brain Cerebellum for Bipolar Disorder and Pancreas for Type 1 Diabetes, and compared the resulting discoveries with those obtained using Whole Blood.

Our results show that tissue-specific references yield gene sets that partially overlap with Whole Blood results but also highlight additional biologically relevant candidates consistent with disease context. For example, brain-derived analyses emphasize neuronal regulatory genes for BD, while pancreas-derived analyses capture immune and stress-response pathways relevant to T1D. These findings demonstrate that IBAS can flexibly incorporate tissue-specific transcriptomic information and recover biologically meaningful signals.

We also clarify that Whole Blood was used as the primary reference due to its larger sample size and broad regulatory coverage, which provide stable estimation of interaction structures (Page 18, line 765). Importantly, our results suggest that different tissues offer complementary insights rather than mutually exclusive signals.

These additions improve both the biological relevance and the practical applicability of the IBAS framework.

*“****Evaluation of tissue-specific transcriptome references in IBAS***

*To assess whether tissue specificity of the reference transcriptome influences IBAS discoveries, we conducted additional analyses using disease-relevant GTEx tissues as alternative reference datasets. Specifically, GTEx Brain Cerebellum expression data were used to train IBAS for the Bipolar Disorder (BD) cohort, and GTEx Pancreas expression data were used for Type 1 Diabetes (T1D). While the primary analyses employed Whole Blood expression due to its broad regulatory coverage, the IBAS framework is not restricted to a single tissue and can naturally incorporate alternative transcriptomic references when biologically appropriate. Across both diseases, tissue-matched references produced gene sets that partially overlapped with Whole Blood discoveries but also revealed additional biologically interpretable candidates, indicating that the interaction structures captured during dimensionality reduction depend on the biological context encoded in the training transcriptome.*

*In the BD analysis, the Brain-derived reference yielded an expanded set of associated genes, several of which have clear functional relevance to neuronal regulatory processes and psychiatric disease biology (****Supplementary Table S6****). These include genes involved in neuronal chromatin regulation (EHMT2), synaptic adhesion and structural connectivity (CTNNA2), and axon guidance and neural signaling (EFNA2), all of which represent plausible mediators of transcriptional programs affecting neural circuitry. The Brain-based analysis also identified multiple immune-related loci, including members of the TNF signaling pathway and the HLA region, consistent with increasing evidence supporting neuroimmune contributions to psychiatric disorders. Additional candidates associated with mitochondrial respiration and neuronal energy metabolism (NDUFS7, ATP6V1G2) further highlight the importance of energy-demanding neuronal processes. Together, these findings indicate that when IBAS is trained on brain-derived transcriptomic interaction patterns, it preferentially highlights genes involved in neural regulatory, immune, and metabolic systems that are biologically consistent with the central nervous system context of BD.*

*To evaluate whether this tissue-dependent pattern extends beyond psychiatric disease, we performed a parallel analysis for T1D using pancreas-derived transcriptomic data. The pancreas-trained model again retained core immune components detectable from Whole Blood, including genes in the TNF pathway and multiple HLA loci, reflecting the established autoimmune basis of T1D (****Supplementary Table S7****). Beyond these shared immune signals, the pancreas-based reference additionally emphasized regulators of cellular stress and inflammatory signaling such as ATF6B, a key mediator of the unfolded protein response implicated in endoplasmic reticulum stress, and ADAM17, an important modulator of cytokine activation and inflammatory signaling. Additional candidates including AGER, an inflammatory receptor associated with metabolic and immune dysfunction, further suggest that pancreas-derived interaction structures capture regulatory programs related to β-cell stress and immune-mediated damage.*

*Importantly, these observations do not suggest that Whole Blood references are inappropriate; rather, they demonstrate that different tissues encode distinct but complementary interaction-mediated regulatory programs. Because IBAS uses the reference transcriptome primarily to derive pathway-level interaction weights rather than to predict expression directly, the method can flexibly accommodate tissue-specific transcriptomic resources such as those provided by GTEx. This flexibility enables biologically informed analyses tailored to disease context when tissue-matched expression data are available, while still allowing robust discoveries using broadly sampled tissues when they provide greater statistical power.”*

*“****Tissue choice influences IBAS discoveries while providing complementary biological insights***

*A critical consideration in the IBAS framework is the choice of reference tissue used to construct the interaction bridge. Our primary discovery analyses relied on Whole Blood transcriptomes, which provide large sample sizes and broad regulatory coverage across common biological pathways. Nevertheless, many complex diseases are expected to involve interaction networks that are partly tissue-specific, particularly in organs directly related to disease pathology. To evaluate this possibility, we performed comparative analyses using disease-relevant GTEx tissues as alternative reference transcriptomes. For Bipolar Disorder, training IBAS on Brain Cerebellum expression data revealed additional genes with clear roles in neuronal regulation and circuitry, including EHMT2 and CTNNA2, supporting the relevance of neural interaction programs. Similarly, for Type 1 Diabetes, using pancreas-derived transcriptomic data emphasized immune and cellular stress regulators such as members of the TNF and HLA pathways together with stress-response genes including ATF6B and inflammatory mediators such as ADAM17, consistent with autoimmune β-cell damage as a central disease mechanism. Importantly, these observations do not imply that Whole Blood is unsuitable; rather, they indicate that different tissues encode complementary interaction-mediated regulatory structures. This flexibility allows researchers to select reference tissues according to disease biology while still leveraging broadly sampled tissues when they offer greater statistical power, thereby enhancing the practical applicability of IBAS across diverse complex traits.”*

Minor Comments

1. The authors emphasize the importance of gene interaction patterns in disease mechanisms, but the manuscript does not provide biological interpretation of the dimensionality reduction results. For example, PCA loadings can reveal which genes contribute most to interaction components. I recommend presenting gene loadings in supplementary materials and linking them to known pathway functions.

We thank the reviewer for this insightful suggestion and agree that biological interpretation of the interaction components is important.

In the revised manuscript, we have added a new section entitled **“PCA loadings identify biologically coherent gene modules within pathways”** (Page 12, line 500). In this section, we analyze gene loadings from principal components and identify the top contributing genes within representative pathways.

We further link these genes to known biological functions. For example, in the MAPK signaling pathway, the leading principal component is driven by heat-shock protein genes (e.g., HSPA family) and immune-related genes, reflecting coordinated stress-response and inflammatory processes. Similarly, in the cytokine–cytokine receptor interaction pathway, top loadings correspond to chemokines and their receptors, capturing coherent immune signaling programs.

These additions demonstrate that the low-dimensional components used in IBAS reflect biologically meaningful gene modules, thereby improving the interpretability of the framework.

*“****PCA loadings identify biologically coherent gene modules within pathways***

*To characterize the biological content of the interaction components generated by dimensionality reduction, we analyzed gene loadings within representative pathways and evaluated whether dominant contributors correspond to known functional modules. We found that genes with the largest absolute loadings consistently formed biologically coherent groups aligned with established pathway functions. In each examined case, the leading principal components were driven by coordinated gene modules reflecting structured pathway-level gene activity. These results demonstrate that the low-dimensional interaction representations derived in IBAS capture biologically organized pathway mechanisms.*

*For a pathway containing* $p$ *genes, PCA decomposes the covariance matrix of the expression matrix into eigenvalues* $\lambda_{1}\geq\lambda_{2}\geq\ldots\geq$ $\lambda_{p}$*and corresponding eigenvectors* $v_{1},v_{2},\ldots,v_{p}$ *. The first principal component (PC1), associated with* $\lambda_{1}$*, captures the maximal variance within the pathway-level expression space. The entries of* $v_{1}=(v_{11},\ldots,v_{1p})^{\top}$*represent gene loadings, quantifying each gene’s contribution to the dominant interaction axis. Genes were ranked by absolute loadings* $\mid v_{1j}\mid$*, as magnitude reflects strength of contribution independent of sign. The top five genes were then evaluated for functional consistency with pathway biology.*

*As an illustrative example, we examined the MAPK signaling pathway (hsa04010), under Environmental Information Processing and Signal Transduction. Ranking genes by absolute loadings of the first principal component identified HSPA1A, HSPA6, CSF1, HSPA1L, and HSPA1B as the strongest contributors to the dominant variance axis (****Supplementary Table S3****). Four of these genes belong to the HSP70 heat-shock protein family, which are well known to be strongly co-expressed under cellular stress conditions. MAPK cascades regulate stress-responsive transcriptional programs, and coordinated induction of heat-shock genes is a characteristic downstream response. In addition, CSF1, a cytokine involved in immune and inflammatory signaling, engages MAPK pathways and participates in coordinated activation programs. The emergence of these genes as top contributors indicates that the principal component captures a structured co-expression pattern consistent with stress and inflammatory signaling within the pathway.*

*A similar pattern was observed in the Cytokine–cytokine receptor interaction pathway (hsa04060), categorized under Environmental Information Processing and Signaling Molecules and Interaction (****Supplementary Table S3****). The genes with the largest absolute loadings included CCL4, IL10RB, TNFRSF1A, CCL3, and CXCR2, comprising chemokines and their cognate receptors central to immune signaling. Chemokines such as CCL3 and CCL4 are frequently co-induced during inflammatory activation, while receptors including CXCR2 and TNFRSF1A mediate coordinated cytokine responses. The concentration of these immune signaling genes among the top loadings indicates that the principal component reflects a coherent inflammatory signaling program within the pathway, arising from shared covariance structure among functionally related genes.*

*Together, these examples demonstrate that principal components derived from pathway-level expression matrices capture coordinated gene activity that aligns with established biological functions. Because IBAS leverages these structured interaction representations as mediators in genotype–phenotype association testing, the method effectively links genetically driven variation in coordinated pathway programs to phenotypic outcomes. This provides both statistical stabilization through dimensionality reduction and mechanistic interpretability grounded in known pathway biology.”*

2. In Figure 4, the overlap of significant genes across methods is very limited. The authors should explain this phenomenon. I suspect it is due to the relatively small genotype sample size (GTEx ~1000 individuals, WTCCC ~5000 samples per disease). Larger datasets such as UK Biobank (>400,000 individuals) should be tested to validate robustness.

We thank the reviewer for this insightful comment. We agree that the limited overlap across methods warrants further explanation.

In the revised manuscript, we clarify that the differences in discoveries arise from the distinct modeling strategies. IBAS incorporates pathway-level interaction information, whereas methods such as PrediXcan and kTWAS primarily rely on single-gene expression signals. As a result, IBAS is able to identify genes with modest marginal effects that are mediated through interaction structures, which may not be detected by conventional approaches.

To further address the concern regarding sample size and robustness, we have added a new section entitled **“Validation of IBAS discoveries in a larger independent T1D cohort”** (Page 15, line 620). Using a substantially larger dataset (10,791 samples) (Page 11, line 443), we demonstrate that a significant proportion of IBAS discoveries are reproducible, while additional known disease-associated genes emerge with increased sample size.

These results suggest that the observed differences across methods are not solely due to different sample size, but also reflect the distinct biological signals captured by IBAS.

*“To further evaluate the performance of IBAS in larger cohorts, we additionally analyzed an independent T1D genome-wide association dataset from dbGaP (Accession: phs000911). This dataset comprises 10,791 samples, substantially exceeding the sample size of the WTCCC T1D cohort (4,901 samples). The inclusion of this dataset enables assessment of the reproducibility and scalability of IBAS findings in a higher-powered setting.”*

*“****Validation of IBAS discoveries in a larger independent T1D cohort***

*To further evaluate whether the discoveries from the WTCCC cohort remain detectable in larger datasets, we applied IBAS to an independent T1D genome-wide association cohort from dbGaP (Accession: phs000911). For comparability with the WTCCC analysis, the same reference data, pathway definitions, and interaction-derived variant weights from GTEx whole blood were used. Genotype preprocessing and quality control followed the same general pipeline as for the WTCCC data.*

*Applying IBAS to the dbGaP cohort produced a set of significant genes that showed substantial concordance with the WTCCC discoveries. The results reported here are based on the PCA IBAS framework with coefficient-based variant weighting, consistent with the primary configuration used in the WTCCC analysis. Approximately 70% of the genes identified in the WTCCC analysis were also detected in the dbGaP dataset, indicating that the interaction-mediated signals uncovered by IBAS are reproducible across independent cohorts despite differences in sample composition and cohort size (****Supplementary Table S5****). The shared genes are strongly enriched in the extended major histocompatibility complex (MHC) region, including multiple classical HLA genes such as HLA-DQA1, HLA-DQB1, HLA-DRB1, HLA-A, HLA-B, and HLA-C, as well as immune-related genes located in the same region such as TNF, LTA, LTB, TAP1, TAP2, and PSMB8. Additional immune-associated genes, such as ITPR3, were also consistently detected across the two cohorts. This pattern is consistent with the well-established role of the MHC region as the dominant genetic contributor to T1D susceptibility.*

*Importantly, the larger dbGaP cohort also revealed additional genes that were not detected in the WTCCC analysis but have strong biological relevance to T1D. These include several canonical non-HLA T1D susceptibility genes such as INS, IL2RA, CTLA4, and ERBB3, which are well known to regulate immune responses and pancreatic β-cell function. The appearance of these established loci in the larger dataset suggests that increasing sample size improves the sensitivity of IBAS to detect genes with moderate effects mediated through interaction patterns. At the same time, the preservation of a substantial subset of WTCCC discoveries demonstrates that IBAS captures stable interaction-mediated genetic signals rather than cohort-specific artifacts.*

*The partial overlap between cohorts is also expected given the complex genetic architecture of T1D. Association signals are highly concentrated within the extended MHC region, where strong linkage disequilibrium and dense gene clusters can lead to multiple correlated genes appearing significant depending on cohort structure and statistical power. Moreover, because IBAS identifies genes whose variants influence interaction structures within biological pathways, rather than relying solely on marginal single-gene effects, the exact ranking of genes can vary across datasets while still reflecting the same underlying interaction-driven genetic mechanisms.*

*Together, these results provide additional support that IBAS identifies biologically meaningful genes underlying T1D and that its discoveries remain detectable when applied to larger and independent cohorts.”*

3. The manuscript does not report the computational complexity of IBAS. I recommend that the authors provide both a time complexity analysis and actual runtime results, and compare them with other methods such as TWAS and MAGMA.

We thank the reviewer for this helpful suggestion. We agree that reporting computational complexity and runtime is important for evaluating the practical applicability of the method.

In the revised manuscript, we have added a new subsection entitled **“Computational Complexity and Runtime Evaluation”** (Page 16, line 709). In this section, we provide both theoretical complexity analysis and empirical runtime comparisons. Specifically, we describe the computational cost of the dimensionality reduction step and the downstream association testing, and report wall-clock runtimes for IBAS, PrediXcan, and MAGMA under the same computational environment.

Our results show that IBAS incurs additional computational cost compared to existing methods, primarily due to the dimensionality reduction step, but this cost enables the extraction of pathway-level interaction patterns that are not captured by standard approaches.

*“****Computational Complexity and Runtime Evaluation***

*IBAS introduces additional computational cost due to its dimensionality reduction step, compared to PrediXcan and MAGMA. For a pathway with* $N$ *samples,* $g$ *genes, and* $m$ *variants, PCA scales on the order of* $\mathcal{O(}Ng^{2}+g^{3})$*, while t-SNE and UMAP typically scale between* $\mathcal{O(}NlogN)$ *and* $\mathcal{O(}N^{2})$*, depending on the implementation. The downstream association and aggregation steps scale approximately linearly with the number of variants, i.e.,* $\mathcal{O(}Nm)$*. In our experiments, MAGMA required 52 seconds, PrediXcan 221.7 seconds, and IBAS 667 seconds, with the additional runtime of IBAS primarily driven by the dimensionality reduction step (~400 seconds). Although IBAS incurs higher computational cost, this additional step enables the extraction of pathway-level interaction patterns through low-dimensional representations, which are not captured by standard TWAS or GWAS approaches. All methods were executed on the same computational node (80 CPU cores; 4 × Intel Xeon Gold 6148 @ 2.40GHz; 3022 GB RAM), ensuring a fair comparison.”*

Reviewer #3: In this paper, Kossina et al. developed IBAS (Interaction-Bridged Association Study), a general framework that incorporates interaction patterns into association mapping without exhaustive testing. IBAS employs statistical learning to derive low-dimensional representations of pathway-level interactions from transcriptomic data, which then serve as mediators in genotype–phenotype association tests. Overall the idea is interesting and reasonable. I have the following questions.

We thank the reviewer for the positive evaluation of our work and for recognizing the rationale and potential of the proposed IBAS framework. We appreciate the reviewer’s thoughtful questions and have addressed them carefully in the revised manuscript. Detailed responses are provided below.

The authors kept saying that their IBAS method is able to capture interaction effects within the pathway. I don’t understand why they are saying this. Is interaction indicate gene-gene interaction? If so, why and how? The intermediate phenotypes, also known as endophenotypes in literature, are principal components (PCs) if using PCA, or some transformed gene expression measures if using t-SNE or UMAP. I don’t think these phenotypes can be interpreted as interactions. The authors need to justify their claim using specific example (can be artificial example) or drop the claim if they are unable to do so.

We thank the reviewer for this important and thoughtful comment. We agree that the term “interaction” requires careful clarification, and that the connection between dimensionality reduction and gene–gene interaction structure should be explicitly justified.

In the revised manuscript, we have added a new section entitled **“Theoretical and Empirical Illustration of PCA for Interaction Structure”** (Page 7, line 291), along with **Fig 2** and **Supplementary Note S1**, to clarify this point.

In our framework, “interaction patterns” primarily refer to structured dependence among genes, reflected in pathway-level expression covariance. Such coordinated activity is a common mode of gene regulation. For example, heat-shock protein genes (e.g., HSPA family) are co-regulated and show strong co-expression under stress (10.1146/annurev.bi.55.070186.005443), while cytokine genes (e.g., CCL3, CCL4, TNF) are jointly activated during immune responses, forming correlated modules (https://doi.org/10.1038/s41467-021-24360-w). Beyond this, more complex interactions also exist. For instance, MYC and MAX act jointly to regulate downstream targets (10.1080/21541264.2025.2453315), and genetic interactions such as synthetic lethality (e.g., BRCA–PARP) reflect nonlinear, non-additive effects (https://doi.org/10.1038/s41586-024-07217-2). While these interactions are difficult to model explicitly, PCA captures dominant covariance structure, and nonlinear methods such as t-SNE and UMAP can approximate more complex patterns by preserving local similarity.

Importantly, this interpretation is consistent with prior work. For example, Böhm et al. (<https://doi.org/10.1145/1007568.1007620>) note that principal component analysis (PCA) is a standard tool for detecting correlations (i.e., linear dependencies) among features, where dominant components represent underlying correlated structures in the data. In the context of gene expression, such correlations are widely interpreted as coordinated or co-regulated gene activity within pathways.

Building on this principle, IBAS applies PCA (or alternative methods) to pathway-level expression matrices to extract dominant patterns in the gene–gene correlation structure. These components summarize coordinated gene modules and are subsequently used to guide SNP weighting and downstream association testing.

These results provide both theoretical and empirical evidence that the low-dimensional representations used in IBAS capture pathway-level coordinated activity arising from gene–gene dependence. More specifically, IBAS leverages the correlation structure of gene expression within pathways, where structured covariance reflects coordinated gene activity. Dimensionality reduction methods such as PCA extract dominant patterns in this correlation structure, which are then used to guide SNP weighting and downstream association testing.

“***Theoretical and Empirical Illustration of PCA for Interaction Structure***

*To demonstrate that PCA can recover gene–gene interaction structure and its associated co-expression patterns, we design a simulation with two gene-expression scenarios.*

*Let* $p=10$ *denote the number of genes in a pathway and* $N=100$ *the number of samples. For each scenario, we generate a gene expression matrix. For each scenario, we generate a gene expression matrix* $X\in\mathbb{R}^{p\times N}$ *, where the* $i$*-th column* $x_{i}\in\mathbb{R}^{p}$ *represents the expression profile of the* $p$ *genes in sample* $i$ *, and*

$x_{i}\mathcal{\sim N}\left( 0,\Sigma\right)$*,* $i=1,...,N$

*In the independent scenario, we set the covariance matrix to be isotropic,* $\Sigma=\Sigma_{0}=\sigma^{2}I_{p}$ *with* $\sigma^{2}=1.44$*, yielding an expression matrix* $X^{\left( 0 \right)}$*. In the interaction scenario, we instead generate an expression matrix* $X^{\left( 1 \right)}$ *under a structured covariance* $\Sigma=\Sigma_{1}$ *, where correlations among a subset of genes are introduced to simulate gene–gene interactions. Specifically,* $\Sigma_{1}$ *contains two disjoint correlated blocks:* $\{gene_{1},gene_{2}\}$ *share pairwise covariance* $\tau$*, and* $\{gene_{3},gene_{4},gene_{5}\}$ *share the same pairwise covariance* $\tau$ *(with* $\tau=1.152$*), while all remaining gene pairs are uncorrelated. Each gene has marginal variance* $\sigma^{2}$*.*

*We then perform PCA by eigen decomposing the empirical gene–gene covariance matrix* $\hat{\Sigma}=\frac{1}{N-1}XX^{T}\in\mathbb{R}^{p\times p}$*. We let* $\Lambda=diag\left( \lambda_{1}\geq\cdots\geq\lambda_{p} \right)$ *to denote the eigenvalues, which quantify the variance explained by each principal component, and let* $V=[v_{1},\ldots,v_{p}]$ *to denote the PC loading vectors.*

*Under* $\Sigma_{1}$*, the two correlated blocks generate inflated leading eigenvalues* $\sigma^{2}+2\tau$ *and* $\sigma^{2}+\tau$ *(****Supplementary Notes S1****), implying that for* $\tau>0$ *, variance concentrates along a small number of dominant eigendirections and the explained-variance ratio* $\lambda_{1}/\sum_{j=1}^{p} \lambda_{j}$ *(and similarly for the next PCs). Correspondingly, the leading loading vectors align with the correlated gene modules, assigning large-magnitude weights to genes* $\{gene_{1},gene_{2}\}$ *and* $\{gene_{3},gene_{4},gene_{5}\}$*. In contrast, under* $\Sigma_{0}$ *(independence), the empirical loadings fluctuate around zero without a reproducible block structure.*

*In our simulation results, PCA clearly distinguishes the interaction and independent scenarios. Under the interaction model, PC1 shows structured loadings with large magnitudes concentrated on genes from the dominant correlated module (****Fig. 2A****), while PC2 captures the remaining module (****Fig. 2B****). Consistent with this behavior, variance is strongly concentrated in the first few principal components in the interaction scenario (****Fig. 2C****), reflecting the emergence of a low-dimensional dependence structure. Theoretically, under the normalized interaction model with within-block correlation* $\rho=0.8$*, the leading eigenvalues are expected to be* $1+2\rho=2.6$ *and* $1+\rho=1.8$ *for the second eigenvalue. In our simulation, the corresponding empirical eigenvalues are* $2.74$ *and* $1.86$*, respectively, closely matching these theoretical predictions and confirming that the interaction structure inflates the leading eigendirections as expected. In contrast, under the independent model, both PC1 and PC2 exhibit loadings that fluctuate around zero with no reproducible block pattern across genes (****Figs. 2A and 2B****), and the explained variance is more evenly distributed across components (****Fig. 2C****).*

*Prior work in data mining has also highlighted PCA as a fundamental tool for detecting correlation structure in high-dimensional data. For example, Böhm et al. [45] note that PCA can “perfectly find” linear correlations between genes, and they build correlation-based clustering methods on top of PCA for applications including gene expression analysis.*

*Beyond PCA, we also examined whether nonlinear manifold learning methods can recover the same interaction-driven dependence patterns. t-distributed stochastic neighbor embedding (t-SNE) is a nonlinear dimension-reduction method that learns a low-dimensional embedding by preserving local neighborhood structure, typically by minimizing a divergence between neighborhood similarities in the original space and in the embedding, e.g.,*

$$minKL(P\parallel Q)=\sum_{i\neq j} p_{ij}log\frac{p_{ij}}{q_{ij}}.$$

*As a result, genes with similar expression profiles tend to be mapped close together. Therefore, when gene–gene interaction structure induces strong within-module correlation, t-SNE tends to place genes from the same correlated module in close proximity, whereas under independence such clustering is not expected. Consequently, when gene–gene interaction structure induces strong within-module correlation, genes in the same module share similar local neighborhoods and are mapped nearby in the t-SNE embedding. In* ***Fig. 2D****, this is reflected by the tight grouping of* $\{gene_{1},gene_{2}\}$ *(red) and the clustering of* $\{gene_{3},gene_{4},gene_{5}\}$ *(purple) under the interaction scenario, whereas under independence the highlighted genes are intermixed without clear module separation.*

*Similarly, Uniform manifold approximation and projection (UMAP) is a nonlinear embedding method that builds a weighted* $k$*-nearest-neighbor graph to represent local neighborhood structure in the original space and then learns a low-dimensional embedding that best preserves these neighbor relationships by minimizing a cross-entropy objective. A common choice for the embedding-space affinity is*

$$\hat{w}_{ij}=\frac{1}{1+a\parallel y_{i}-y_{j}\parallel^{2b}}.$$

*thus, when gene–gene interaction structure induces strong within-module correlation, genes within the same module tend to share local neighborhoods and are mapped close together, whereas under independence this clustering behavior is not expected. Under the interaction scenario, correlated genes receive consistently higher neighbor weights and form stable local neighborhoods, causing UMAP to place them in close proximity and yield module-level clustering. In contrast, under independence, neighbor relationships are weaker and less structured, producing embeddings with less coherent gene grouping (****Fig. 2E****).*

*Although the above derivation assumes an idealized equi-covariance block structure, it illustrates a general mechanism: dependence among genes induces dominant eigendirections in the covariance (or correlation) matrix, concentrating variance into a small number of principal components. In more general settings with heterogeneous correlations, PCA no longer admits closed-form eigenpairs, but the leading PCs still recover the dominant low-dimensional dependence patterns. Beyond PCA, nonlinear embedding methods such as t-SNE and UMAP can also reflect gene–gene interaction structure by preserving local neighborhood relationships among genes. When interaction-driven co-expression induces within-module similarity, these methods tend to map genes from the same correlated module to nearby locations in the embedding space, producing module-level clustering patterns.”*

# ***“Figure 2 – PCA and manifold learning reveal interaction-induced gene modules.***

***A–B)*** *PC1 and PC2 loadings for a simulated pathway under independent and interaction (block-correlated) scenarios. Under interaction, loadings reflect module structure (*$\{gene_{3},gene_{4},gene_{5}\}$ *for PC1;* $\{gene_{1},gene_{2}\}$ *for PC2), while under independence they appear unstructured.*
***C)*** *Variance explained by PCs, showing stronger concentration in leading components under interaction.*
***D–E)*** *t-SNE and UMAP embeddings. Correlated genes cluster by module under interaction (Pink and Yellow) but show no clear grouping under independence.*”

# ***“Supplementary Notes S1 – Spectral Properties of Equi-Correlation Covariance Structures***

***Eigenstructure of an equi-covariance block***

*Under the interaction scenario, consider a correlated block of size* $m$ *in the population covariance matrix, where each gene pair has covariance* $\tau$ *(assume* $\tau> 0$*). The corresponding* $m\times m$ *covariance submatrix can be written as*

$\Sigma_{block}=\left[ \begin{matrix} \begin{matrix} \begin{matrix} \begin{matrix} \sigma^{2} & \tau\end{matrix} \\ \begin{matrix} \tau& \sigma^{2} \end{matrix} \end{matrix} & \begin{matrix} \begin{matrix} \ldots& \tau\end{matrix} \\ \begin{matrix} \ldots& \tau\end{matrix} \end{matrix} \end{matrix} \\ \begin{matrix} \begin{matrix} \begin{matrix} \vdots& \vdots\end{matrix} \\ \begin{matrix} \tau& \tau\end{matrix} \end{matrix} & \begin{matrix} \begin{matrix} \ddots& \vdots\end{matrix} \\ \begin{matrix} \ldots& \sigma^{2} \end{matrix} \end{matrix} \end{matrix} \end{matrix} \right]=\left( \sigma^{2}-\tau\right)I_{m}+{\tau\boldsymbol{1}_{\boldsymbol{m}}\boldsymbol{1}}_{\boldsymbol{m}}^{\boldsymbol{T}}$ *,*

*where* $\boldsymbol{1}_{m}$ *denotes the* $m$*-dimensional all-ones vector.*

***Claim A:*** $\Sigma_{block}$ *has top eigenvector* $\boldsymbol{1}_{\boldsymbol{m}}$*.*

$\Sigma_{block}\boldsymbol{1}_{\boldsymbol{m}}=\left( \sigma^{2}+(m-1) \tau\right)\boldsymbol{1}_{\boldsymbol{m}}$ *,*

***Proof.*** *Use* $\boldsymbol{1}_{m}^{\top}\boldsymbol{1}_{m}=m$ *and* $(\boldsymbol{1}_{m}\boldsymbol{1}_{m}^{\top})\boldsymbol{1}_{m}=\boldsymbol{1}_{m}(\boldsymbol{1}_{m}^{\top}\boldsymbol{1}_{m})=m\boldsymbol{1}_{m}$*:*

$$\Sigma_{\text{block}}\boldsymbol{1}_{m}=(\sigma^{2}-\tau)\boldsymbol{1}_{m}+\tau(\boldsymbol{1}_{m}\boldsymbol{1}_{m}^{\top})\boldsymbol{1}_{m}=(\sigma^{2}-\tau)\boldsymbol{1}_{m}+\tau m\boldsymbol{1}_{m}=(\sigma^{2}+(m-1)\tau)\boldsymbol{1}_{m}.$$

*So* $\boldsymbol{1}_{m}$*is an eigenvector with eigenvalue* $\sigma^{2}+(m-1)\tau$*.*

***Claim B:*** $\Sigma_{block}$ *has eigenvector* $u$*.*

*For any* $w\in\mathbb{R}^{m}$ *with* $\boldsymbol{1}_{m}^{\top}u=0$*,*

$$\Sigma_{\text{block}}u=(\sigma^{2}-\tau)u.$$

***Proof.*** *If* $\boldsymbol{1}_{m}^{\top}w=0$*, then* $(\boldsymbol{1}_{m}\boldsymbol{1}_{m}^{\top})w=\boldsymbol{1}_{m}(\boldsymbol{1}_{m}^{\top}w)=0$*. Hence*

$$\Sigma_{\text{block}}w=(\sigma^{2}-\tau)w+\tau(\boldsymbol{1}_{m}\boldsymbol{1}_{m}^{\top})w=(\sigma^{2}-\tau)w.$$

*So* $u$ *that orthogonal to* $\boldsymbol{1}_{m}$*is an eigenvector with eigenvalue* $\sigma^{2}-\tau$*. This eigenspace has dimension* $m-1$*.*

*If* $\tau>0$*, then*

$$\sigma^{2}+(m-1)\tau\text{ }>\text{ }\sigma^{2}-\tau,$$

*so the largest eigenvalue of the block is* $\sigma^{2}+(m-1)\tau$*, and the other eigenvalue* $\sigma^{2}-\tau$ *has multiplicity* $m-1$*. (If* $\tau<0$*, the ordering flips.)*

***Extension to block-diagonal covariance matrices***

*Since* $\Sigma_{1}$ *is block-diagonal, the eigenpairs of each block lift to eigenpairs of the full matrix by padding zeros.*

*Let the first block be* $B\in\mathbb{R}^{m\times m}$*, also define the eigen vector of* $B$ *by* $w_{1},\ldots,w_{m}$*. Let the full* $p$*-dimensional vectors:*

$$u_{i}=(w_{i},0,\ldots,0)^{\top}.$$

*First note that* $Bw_{i}=\varphi_{i}w_{i}, i = 1,...,m$*, so* $\varphi_{i}$ *are eigenvalues of the block* $B$*.*

*Now write a diagonal block* $\Sigma$ *in form:*

$$\Sigma=\left( \begin{matrix} B & 0 \\ 0 & C \end{matrix} \right),$$

*where* $C$ *is the covariance matrix for the rest of genes. Then:*

$\Sigma u_{i}=\left( \begin{matrix} B & 0 \\ 0 & C \end{matrix} \right)\left( \begin{aligned} w_{i} \\ 0 \end{aligned} \right)=\left( \begin{aligned} Bw_{i} \\ 0 \end{aligned} \right)=\varphi_{i}\text{ }\left( \begin{aligned} w_{i} \\ 0 \end{aligned} \right)=\varphi_{i}u_{i}$*.*

*So* $u_{i}$ *is an eigenvector of the full* $\Sigma$*.*

***Eigenstructure of*** $\boldsymbol{\Sigma}_{\boldsymbol{1}}$***with two disjoint correlated blocks***

*Similarly, In our case* $\Sigma_{1}$*has two disjoint correlated blocks:* $\{gene_{1},gene_{2}\}$*(size* $m_{1}=2$*) and* $\{gene_{3},gene_{4},gene_{5}\}$ *(size* $m_{2}=3$*), and all cross-block covariances are 0. The remaining genes (6–10) are independent with variance* $\sigma^{2}$*. Then* $\Sigma_{1}$*is block diagonal, so its eigenvalues are the union of eigenvalues from each diagonal block.*

*For block* $\{gene_{1},gene_{2}\}$*: eigenvalues* $\sigma^{2}+(2-1)\tau=\sigma^{2}+\tau$ *(mult. 1) and* $\sigma^{2}-\tau$ *(mult. 1).*

*For block* $\{gene_{3},gene_{4},gene_{5}\}$*: eigenvalues* $\sigma^{2}+(3-1)\tau=\sigma^{2}+2\tau$ *(mult. 1) and* $\sigma^{2}-\tau$ *(mult. 2).*

*So, the top and second eigen value should be:* $\sigma^{2}+2\tau$ *and* $\sigma^{2}+\tau$*, thereby concentrating explained variance in the first few PCs and yielding loading vectors with large magnitude on the correlated genes.*

*In our interaction scenario we take* $\tau>0$*, corresponding to positively correlated (co-expressed) genes. When* $\tau<0$*, the largest eigenvalue is attained in the subspace orthogonal to* $\boldsymbol{1}_{m}$*, yielding contrast directions with mixed-sign loadings rather than a single co-expression direction.”*

I am able to follow the method description up to the point that a gene can be associated with a principal component for the gene expression matrix for a pathway. But from that point, how to connect to a phenotype is confusing. Since there are thousands of pathways. For each gene, there will be hundreds or thousands of association signals, one for each pathway. Not mentioning multiple “interaction components” within each pathway. The authors need to describe how to integrate association signals to calculate association with a phenotype.

We thank the reviewer for this important comment. We agree that the integration of association signals across pathways and interaction components should be more clearly described.

In the revised manuscript, we have clarified this process in the section “**IBAS Implementation (IV): Genotype–Phenotype Association Test via Feature Aggregation**” (Page 6, line 241).

Specifically, IBAS does not perform association testing separately for each pathway and then combine p-values across pathways. Instead, pathway-level interaction components are used to construct SNP-level weights, which are then mapped to the cis-SNPs of each gene. When multiple interaction components are considered, their contributions are first aggregated into a single meta-weight per SNP (e.g., via coefficient-based averaging or Fisher-based combination).

These SNP-level meta-weights are then used within a gene-level kernel (SKAT) test, producing a single association statistic per gene. Therefore, although multiple pathways and components contribute to weight construction, the final association is evaluated at the gene level, yielding one p-value per gene.

We have revised the manuscript to make this aggregation procedure explicit and to clarify the connection between pathway-level representations and gene-level phenotype association.

## *“****IBAS Implementation (IV): Genotype-Phenotype Association Test via Feature Aggregation.*** *Finally, gene-level testing is conducted using the Sequence Kernel Association Test (SKAT). This test combines the effect of multiple variants in a kernel-based score test to test phenotypic association of the aggregate:*

$Q=\boldsymbol{y}^{T}\boldsymbol{K}_{\boldsymbol{s}}\boldsymbol{y}$	*(2)*

*where* $\boldsymbol{y}\in\mathbb{R}^{N\times1}$ *is the normalized phenotype vector, and* $\boldsymbol{K}_{\boldsymbol{s}}\in\mathbb{R}^{N\times N}$ *is the kernel matrix constructed from the centralized genotype matrix* $\boldsymbol{G}\in\mathbb{R}^{N\times c}$ *in the GWAS dataset, where* $c$ *is the number of cis-variants of a gene. In the original SKAT framework, the kernel is defined as*

$$\boldsymbol{K}_{\boldsymbol{s}}=\boldsymbol{G}\boldsymbol{W}_{SKAT}\boldsymbol{G}^{\top},$$

*, and* $\boldsymbol{W}_{SKAT}=diag(w_{1},\ldots,w_{c})$ *is a diagonal* $c$ *by* $c$ *weight matrix. The weights* $w_{j}$ *are typically assigned based on minor allele frequency using a Beta distribution.*

*While multiple kernels may be defined for the use in the score test [27], we use the modified kernel previously used in our work [18,20]:*

$\boldsymbol{K}_{\boldsymbol{w}}=\boldsymbol{G}\boldsymbol{W}_{\boldsymbol{I}BAS}\boldsymbol{G}^{\top}$	*(3)*

*where* $\boldsymbol{W}_{IBAS}=diag(\alpha_{1},\ldots,\alpha_{i},\ldots,\alpha_{c})$*.* $\alpha_{i}$ *here, denotes the coefficients of association derived from Equation 1 for the reference data. These coefficients of association may be selected as the direct coefficients from the linear mixed model (i.e.* $\alpha_{i}=\left| \beta_{i} \right|$*; coefficient-based weighting) or as the p-values associated with each coefficient from the same (i.e.* $\alpha_{i}=-\log p_{i}$*; p-value based weighting). This is then used in Equation 2 to calculate Q which follows a mixture of chi-square distributions [44] (Fig 1C).*

*When a single interaction component (e.g., the first principal component) is used, SNP weights reflect the association between genetic variants and a single dominant interaction pattern within the pathway. However, complex biological interactions are often distributed across multiple components, and relying on a single component may fail to capture the full interaction structure. To address this, we incorporate multiple interaction components and aggregate their corresponding SNP-level weights.*

*Specifically, for coefficient-based weighting, the meta-weight for SNP* $i$ *(*$i =1,2,...,c$*) is defined as*

$$\tilde{w}_{i}=\frac{1}{K}\sum_{k=1}^{K} {|\beta}_{ik}|,$$

*where* $\beta_{ik}$ *is the regression coefficient of SNP* $i$*obtained from the* $k$*-th interaction component, and* $K$ *is the number of components (e.g., the top 10 principal components).*

*For p-value-based weighting, we combine the evidence across components using Fisher’s method:*

$$X_{i}=-2\sum_{k=1}^{K} log(p_{ik}),$$

*where* $p_{ik}$ *is the p-value corresponding to SNP* $i$ *for the* $k$*-th component. Under the null hypothesis,* $X_{i}\sim\chi_{2K}^{2}$*, from which a combined p-value is obtained and subsequently transformed into SNP weights.*

*These meta-weights summarize the overall contribution of each SNP to the dominant interaction structures within the pathway. Unless otherwise specified, coefficient-based aggregation is used in this study.*

*IBAS performs association testing at the gene level. For each gene within a pathway, SNP-level weights derived from pathway-informed interaction components are applied to its cis-SNPs, and a gene-level association statistic is computed using a weighted SKAT framework. Thus, similar to TWAS, the basic unit of association in IBAS is the gene rather than individual SNPs.*

*The output of IBAS is therefore a set of genes associated with the phenotype of interest, mediated through pathway-level interaction effects. As each pathway may contain a large number of genes, multiple hypothesis testing correction is performed using the Benjamini–Hochberg procedure[45] to control false discoveries.”*

I am also confused about what is the basic element for association in IBAS. For GWAS, association is calculated at the SNP level. For TWAS, association is calculated at the gene level. I wonder what about IBAS? Can you show a Manhattan plot for IBAS? This will clarify lots of confusion.

We thank the reviewer for this helpful comment. We agree that clarifying the unit of association in IBAS is important.

In the revised manuscript, we explicitly state that IBAS performs gene-level association testing, similar to TWAS (Page 7, line 282). Specifically, for each gene, SNP-level weights derived from pathway-level interaction components are applied to its cis-SNPs, and a gene-level association statistic is computed using a weighted SKAT framework. Thus, the basic unit of association in IBAS is the gene rather than individual SNPs.

To further improve clarity, we have added Manhattan plots of gene-level association results (Page 15, line 616; **Fig. 8**; **Supplementary Fig. S7–S12**), where each point represents a gene positioned by its genomic location and evaluated using the IBAS test statistic. These plots provide a genome-wide view of IBAS discoveries and facilitate direct comparison with standard GWAS-style visualizations.

*“IBAS performs association testing at the gene level. For each gene within a pathway, SNP-level weights derived from pathway-informed interaction components are applied to its cis-SNPs, and a gene-level association statistic is computed using a weighted SKAT framework. Thus, similar to TWAS, the basic unit of association in IBAS is the gene rather than individual SNPs.*

*The output of IBAS is therefore a set of genes associated with the phenotype of interest, mediated through pathway-level interaction effects. As each pathway may contain a large number of genes, multiple hypothesis testing correction is performed using the Benjamini–Hochberg procedure[45] to control false discoveries.”*

*“To further clarify the unit of association in IBAS, we present Manhattan plots of gene-level association results across the genome. Each point represents a gene, with its genomic position defined by gene location and its significance determined by the IBAS gene-level test statistic (Supplementary Fig. S7 – S12).”*

# *“****Figure 8 – Gene-level Manhattan plot for type 1 diabetes (T1D) in the WTCCC dataset using IBAS.***

*Gene-level Manhattan plot for the WTCCC type 1 diabetes (T1D) dataset using the IBAS framework. Each point represents a gene, plotted by chromosomal position (x-axis) and its association significance measured as* $-{log}_{10} \left( p \right)$ *(y-axis). Gene-level statistics were constructed using PCA-based interaction components and coefficient-based variant weighting, with expression weights derived from GTEx Whole Blood tissue. The labeled genes correspond to the top 10 most significant associations.*

# ***Supplementary Figure S7 – Gene-level Manhattan plot for bipolar disorder (BD) in the WTCCC dataset using IBAS.***

*Gene-level Manhattan plot for the WTCCC bipolar disorder (BD) dataset using the IBAS framework. Each point represents a gene, plotted by chromosomal position (x-axis) and its association significance measured as* $-{log}_{10} \left( p \right)$ *(y-axis). Gene-level statistics were constructed using PCA-based interaction components and coefficient-based variant weighting, with expression weights derived from GTEx Whole Blood tissue. The labeled genes correspond to the top 10 most significant associations.*

# ***Supplementary Figure S8 – Gene-level Manhattan plot for coronary artery disease (CAD) in the WTCCC dataset using IBAS.***

*Gene-level Manhattan plot for the WTCCC coronary artery disease (CAD) dataset using the IBAS framework.*

# ***Supplementary Figure S9 – Gene-level Manhattan plot for Crohn’s disease (CD) in the WTCCC dataset using IBAS.***

*Gene-level Manhattan plot for the WTCCC Crohn’s disease (CD) dataset using the IBAS framework.*

# ***Supplementary Figure S10 – Gene-level Manhattan plot for rheumatoid arthritis (RA) in the WTCCC dataset using IBAS.***

*Gene-level Manhattan plot for the WTCCC rheumatoid arthritis (RA) dataset using the IBAS framework.*

# ***Supplementary Figure S11 – Gene-level Manhattan plot for type 2 diabetes (T2D) in the WTCCC dataset using IBAS.***

*Gene-level Manhattan plot for the WTCCC type 2 diabetes (T2D) dataset using the IBAS framework.*

# ***Supplementary Figure S12 – Gene-level Manhattan plot for hypertension (HT) in the WTCCC dataset using IBAS.***

*Gene-level Manhattan plot for the WTCCC hypertension (HT) dataset using the IBAS framework. ”*

Related to the above points, how do you comparison performance between TWAS and IBAS?

We thank the reviewer for this important question. We agree that a fair comparison requires consistent units of analysis and comparable testing frameworks.

In the revised manuscript, we clarify that all methods considered, including IBAS and TWAS-based approaches (e.g., PrediXcan and kTWAS), perform gene-level association testing using cis-SNPs (Page 11, line 460). This ensures that comparisons are made on the same unit of inference.

The key distinction lies in how SNP-level weights are constructed. TWAS methods derive weights from gene-specific genotype–expression prediction models, whereas IBAS derives weights from pathway-level interaction components obtained through dimensionality reduction. These weights are then incorporated into a common gene-level association framework.

Performance is compared based on the resulting gene-level associations, including measures such as stability under perturbation, reproducibility across datasets, and biological interpretability. This setup allows for a direct and fair comparison between IBAS and existing TWAS methods.

### *“All methods considered, including IBAS, perform gene-level association testing using cis-SNPs within a kernel-based framework. The key distinction lies in how SNP-level weights are constructed. In PrediXcan and kTWAS, weights are derived from gene-specific genotype–expression models, whereas IBAS obtains weights from regression on pathway-level interaction components. Thus, IBAS incorporates pathway-level information while retaining gene-level testing.”*

I do not quite get the points the authors are trying to make in Figures 2,3 and 4. How to connect associational signals of “interaction components” to genes?

We thank the reviewer for this important comment. We agree that the interpretation of **Figures 3, 5 and 6** (Originally Figures 2–4) and the connection between interaction components and gene-level association can be further clarified.

Figure 3 illustrates the stability of gene-level association results produced by IBAS. Across perturbations of the expression data, IBAS shows reduced variance in gene-level p-values and higher recurrence of significant genes, indicating improved robustness of the interaction-guided weighting strategy. Figure 5 highlights the biological relevance of the identified genes, distinguishing previously validated genes from potentially novel candidates. Figure 6 compares gene-level results across methods, demonstrating that IBAS captures signals consistent with existing approaches while also identifying additional candidates.

Conceptually, IBAS does not directly test interaction components for association with the phenotype. Instead, it uses these components to construct an intermediate layer linking pathway-level expression structure to gene-level association. Specifically, interaction components (e.g., principal components derived from pathway-level expression data) are used to estimate SNP-level weights by modeling the relationship between genotype and each component. When multiple components are considered, their contributions are aggregated into a single meta-weight per SNP. These SNP-level weights are then applied to the cis-SNPs of each gene, and a gene-level association test is performed using a weighted SKAT framework. In this way, pathway-level interaction patterns influence gene-level association through SNP weighting rather than direct testing.

In the revised manuscript, we have clarified this pipeline in the section **“IBAS Implementation (IV): Genotype–Phenotype Association Test via Feature Aggregation”** (Page 6, line 241).

## *“****IBAS Implementation (IV): Genotype-Phenotype Association Test via Feature Aggregation.*** *Finally, gene-level testing is conducted using the Sequence Kernel Association Test (SKAT). This test combines the effect of multiple variants in a kernel-based score test to test phenotypic association of the aggregate:*

$Q=\boldsymbol{y}^{T}\boldsymbol{K}_{\boldsymbol{s}}\boldsymbol{y}$	*(2)*

*where* $\boldsymbol{y}\in\mathbb{R}^{N\times1}$ *is the normalized phenotype vector, and* $\boldsymbol{K}_{\boldsymbol{s}}\in\mathbb{R}^{N\times N}$ *is the kernel matrix constructed from the centralized genotype matrix* $\boldsymbol{G}\in\mathbb{R}^{N\times c}$ *in the GWAS dataset, where* $c$ *is the number of cis-variants of a gene. In the original SKAT framework, the kernel is defined as*

$$\boldsymbol{K}_{\boldsymbol{s}}=\boldsymbol{G}\boldsymbol{W}_{SKAT}\boldsymbol{G}^{\top},$$

*, and* $\boldsymbol{W}_{SKAT}=diag(w_{1},\ldots,w_{c})$ *is a diagonal* $c$ *by* $c$ *weight matrix. The weights* $w_{j}$ *are typically assigned based on minor allele frequency using a Beta distribution.*

*While multiple kernels may be defined for the use in the score test [27], we use the modified kernel previously used in our work [18,20]:*

$\boldsymbol{K}_{\boldsymbol{w}}=\boldsymbol{G}\boldsymbol{W}_{\boldsymbol{I}BAS}\boldsymbol{G}^{\top}$	*(3)*

*where* $\boldsymbol{W}_{IBAS}=diag(\alpha_{1},\ldots,\alpha_{i},\ldots,\alpha_{c})$*.* $\alpha_{i}$ *here, denotes the coefficients of association derived from Equation 1 for the reference data. These coefficients of association may be selected as the direct coefficients from the linear mixed model (i.e.* $\alpha_{i}=\left| \beta_{i} \right|$*; coefficient-based weighting) or as the p-values associated with each coefficient from the same (i.e.* $\alpha_{i}=-\log p_{i}$*; p-value based weighting). This is then used in Equation 2 to calculate Q which follows a mixture of chi-square distributions [44] (Fig 1C).*

*When a single interaction component (e.g., the first principal component) is used, SNP weights reflect the association between genetic variants and a single dominant interaction pattern within the pathway. However, complex biological interactions are often distributed across multiple components, and relying on a single component may fail to capture the full interaction structure. To address this, we incorporate multiple interaction components and aggregate their corresponding SNP-level weights.*

*Specifically, for coefficient-based weighting, the meta-weight for SNP* $i$ *(*$i =1,2,...,c$*) is defined as*

$$\tilde{w}_{i}=\frac{1}{K}\sum_{k=1}^{K} {|\beta}_{ik}|,$$

*where* $\beta_{ik}$ *is the regression coefficient of SNP* $i$*obtained from the* $k$*-th interaction component, and* $K$ *is the number of components (e.g., the top 10 principal components).*

*For p-value-based weighting, we combine the evidence across components using Fisher’s method:*

$$X_{i}=-2\sum_{k=1}^{K} log(p_{ik}),$$

*where* $p_{ik}$ *is the p-value corresponding to SNP* $i$ *for the* $k$*-th component. Under the null hypothesis,* $X_{i}\sim\chi_{2K}^{2}$*, from which a combined p-value is obtained and subsequently transformed into SNP weights.*

*These meta-weights summarize the overall contribution of each SNP to the dominant interaction structures within the pathway. Unless otherwise specified, coefficient-based aggregation is used in this study.*

*IBAS performs association testing at the gene level. For each gene within a pathway, SNP-level weights derived from pathway-informed interaction components are applied to its cis-SNPs, and a gene-level association statistic is computed using a weighted SKAT framework. Thus, similar to TWAS, the basic unit of association in IBAS is the gene rather than individual SNPs.*

*The output of IBAS is therefore a set of genes associated with the phenotype of interest, mediated through pathway-level interaction effects. As each pathway may contain a large number of genes, multiple hypothesis testing correction is performed using the Benjamini–Hochberg procedure (Benjamini and Hochberg 1995) to control false discoveries.*”

I felt that the Discussion section was not fully developed. The pros and cons of the IBAS method should be discussed at least.

We thank the reviewer for this helpful suggestion. We agree that a balanced discussion of the strengths and limitations of IBAS is important.

In the revised manuscript, we have added a new subsection entitled **“Strengths and Limitations of IBAS”** (Page 18, line 786). In this section, we highlight key advantages of the framework, including its ability to capture pathway-level interaction structure through dimensionality reduction without explicitly modeling combinatorial interactions, and its flexibility in incorporating different representation learning methods and weighting strategies.

We also discuss several limitations, including the dependence on the quality and relevance of reference transcriptomic data, the presence of tuning parameters in the dimensionality reduction step, reliance on pathway annotations, and the fact that IBAS captures interaction structure indirectly rather than modeling specific interaction pairs.

*“****Strengths and Limitations of IBAS***

*IBAS offers several methodological advantages for association studies involving gene–gene interactions. First, by leveraging dimensionality reduction, IBAS captures structured interaction patterns at the pathway level without explicitly enumerating combinatorial interactions, thereby avoiding the severe statistical and computational burden associated with high-order interaction modeling. Second, the use of pathway-informed representation learning enables IBAS to incorporate biologically meaningful correlation structures into SNP weighting, which can improve power compared to methods that rely solely on gene-level or SNP-level signals. Third, the modular design of IBAS allows flexibility in choosing representation learning techniques (e.g., PCA, UMAP, t-SNE) and weighting strategies, making the framework adaptable to different data types and biological contexts.*

*Despite these advantages, IBAS also has several limitations. The method depends on the quality and relevance of the reference transcriptomic data, including tissue selection and sample size, which may influence the extracted interaction patterns. In addition, the dimensionality reduction step introduces tuning parameters (e.g., number of components or embedding parameters), and while our results suggest robustness to reasonable choices, suboptimal settings may affect performance. Furthermore, IBAS relies on pathway annotations, and incomplete or inaccurate pathway definitions may limit its ability to capture relevant interaction structures. Finally, while IBAS improves stability by summarizing interaction patterns, it does not explicitly model specific interaction pairs, which may limit interpretability at the individual interaction level.”*

Lines 277-278, “Since IBAS works best if there is a high degree of overlap between the GWAS and reference datasets when considering genotypes”. I do not understand what does “overlap” means here?

We thank the reviewer for pointing out this ambiguity. We agree that the term “overlap” was not sufficiently clear in the original manuscript.

In the revised manuscript, we have clarified that “overlap” refers to the shared SNP positions between the GWAS dataset and the reference dataset used for expression modeling (Page 10, line 424). A higher degree of overlap ensures that sufficient variants are available for constructing SNP-level weights and performing downstream association testing.

*“The GWAS genotype dataset consisted of 838 individuals and 43,066,422 SNPs in the raw VCF data. Since IBAS relies on SNP-level information shared between the GWAS dataset and the reference dataset used for expression modeling, a high degree of overlap in SNP positions is desirable to ensure sufficient variants are available for weighting and association testing.”*

Line 271, it says GWAS “contains samples from 670 individuals”. But in line 276, “Genotype data for 838 individuals and 43,066,422 SNPs were present in the raw VCF data.” This is contradictory.

We thank the reviewer for pointing out this confusion. We agree that the description in the original manuscript was not sufficiently clear.

In the revised manuscript, we have clarified that these numbers refer to two different datasets (Page 10, line 415; Page 10, line 424). Specifically, the 670 individuals correspond to the GTEx Whole Blood expression dataset used as the reference for expression modeling, while the 838 individuals refer to the GWAS genotype dataset used for association testing.

We note that it is common in genomic studies for expression datasets to contain fewer samples than genotype datasets, as transcriptomic measurements are more resource-intensive to obtain. We have revised the text to clearly distinguish these datasets and avoid ambiguity.

*“Publicly available, normalized, and filtered expression data from the “Whole Blood” tissue of the GTEx Consortium [19] , comprising 670 individuals and 20,315 transcripts, were used as the reference expression dataset.”*

*“The GWAS genotype dataset consisted of 838 individuals and 43,066,422 SNPs in the raw VCF data.”*
