# Supplementary material for: IBAS: Interaction-bridged association studies discovering novel genes underlying complex traits
Source: PLoS Comput Biol. 2026 Aug 17;22(8):e1014640. doi: 10.1371/journal.pcbi.1014640 (PMC13480648; doi:10.1371/journal.pcbi.1014640)
Supplement: Supplementary file 18 [file pcbi.1014640.s025.docx]

**Reviewer's Responses to Questions**

**Reviewer #1:** This is one of the most responsive revisions I have seen! A huge “thank you” to the authors! I am satisfied and pleased with the revision!

We sincerely thank the reviewer for the very positive and encouraging evaluation of our revised manuscript! We are grateful that the reviewer found the revision responsive and satisfactory!

I also just realize that what the authors mean by “gene-gene interaction” is coordinated or co-regulated gene expression, or simply co-expression, instead of typical statistical “interaction” or genetic “epistasis” that I thought it meant; if so, then why dimension reduction techniques like PCA work is more obvious; that’s also why “meta-genes” or “modules” are used in other related techniques/papers. I don’t want to cause more troubles, but feel that if it is possible that the authors can clarify this at the beginning, it would make it much easier to understand; I leave it to the authors to decide.

We thank the reviewer for pointing out the potential ambiguity in the term “gene–gene interaction.” We agree that, in IBAS, this term refers to coordinated or co-regulated transcriptional activity within pathways, rather than statistical epistasis or explicit pairwise interaction terms. To address this, we revised the Introduction (Page 2, Line 74; Page 3, Line 129) to clarify this terminology and to explain that dimensionality reduction methods are used to summarize structured co-expression or covariance patterns into interaction-informed mediators for downstream association analysis.

*“…In this study, the term gene–gene interaction refers to coordinated transcriptional activity within biological pathways, reflected by structured co-expression or covariance patterns among genes, rather than explicit statistical interaction or genetic epistasis between individual genes or variants. Our objective is therefore not to explicitly model pairwise interaction terms, but to learn low-dimensional representations that summarize these coordinated pathway-level expression programs.”*

*“…Linear methods such as PCA naturally capture dominant covariance structures, while nonlinear approaches including t-SNE and UMAP can additionally preserve complex local relationships induced by coordinated expression patterns. These learned representations serve as interaction-informed mediators that bridge transcriptomic organization and downstream genotype–phenotype association analysis.”*

**Reviewer #2:** I thank the authors for the careful revision. The new Supplementary Note S1, the Type I error analysis, the tissue-specific analyses, the dbGaP replication, and the additional benchmarks together represent a clear improvement, and most of my original concerns have been addressed. Two small points remain.

We sincerely thank the reviewer for the careful evaluation and for recognizing the improvements made in the revised manuscript. We are grateful that most of the original concerns have been addressed, and we respond to the two remaining points below.

1. Supplementary Note S1 elegantly shows that PCA recovers block-correlated covariance, i.e., co-expression structure. This is distinct from gene–gene interaction in the statistical-genetics sense. I suggest either softening the terminology throughout to "coordinated activity" or "co-expression structure", or acknowledging this scope in the Introduction.

We thank the reviewer for this helpful suggestion. We agree that, in IBAS, this term refers to coordinated or co-regulated transcriptional activity within pathways, rather than statistical epistasis or explicit pairwise interaction terms. To clarify the scope of our terminology, we revised the Introduction (Page 2, Line 74; Page 3, Line 129) to state that “gene–gene interaction” in IBAS refers to coordinated or co-regulated transcriptional activity within pathways, reflected by structured co-expression or covariance patterns, rather than statistical epistasis or explicit pairwise interaction terms. We also clarified that dimensionality reduction methods summarize these coordinated expression patterns into interaction-informed mediators for downstream association analysis.

*“…In this study, the term gene–gene interaction refers to coordinated transcriptional activity within biological pathways, reflected by structured co-expression or covariance patterns among genes, rather than explicit statistical interaction or genetic epistasis between individual genes or variants. Our objective is therefore not to explicitly model pairwise interaction terms, but to learn low-dimensional representations that summarize these coordinated pathway-level expression programs.”*

*“…Linear methods such as PCA naturally capture dominant covariance structures, while nonlinear approaches including t-SNE and UMAP can additionally preserve complex local relationships induced by coordinated expression patterns. These learned representations serve as interaction-informed mediators that bridge transcriptomic organization and downstream genotype–phenotype association analysis.”*

2. Fig. 3 demonstrates stability under perturbation, but a method that detects nothing would also be perfectly stable. Could the authors add a simulation in which a known set of causal genes is planted within a pathway, and report the empirical power (proportion of these recovered) for IBAS vs. competitors at a fixed significance level?

We thank the reviewer for this important suggestion. We agree that stability alone does not fully demonstrate method performance, since a method with few or no discoveries could also appear stable. To address this concern, we added a new empirical power simulation with predefined causal genes planted within pathway-level structures.

Specifically, we added a new Methods section, “**Simulation Framework for Empirical Power Evaluation**” (Page 10, Line 420), in which causal genes were selected from pathway, and phenotypes were generated under additive, hybrid, and interaction architectures across multiple heritability levels. We then compared the ability of IBAS and competing methods, including SKAT-O, PrediXcan, and MAGMA, to recover the planted causal genes after multiple-testing correction. We defined empirical power as the proportion of planted causal genes recovered after Benjamini–Hochberg correction at FDR 0.05.

We also added a new Results section, “**IBAS improves empirical power in simulation studies**” (Page 13, Line 562), together with the new **Figure 5**. The results show that IBAS using PCA-derived weights achieved comparable or higher power under the additive model and showed increasingly clear advantages under the hybrid and interaction models. These findings indicate that the stability observed in IBAS is not due to a lack of discoveries, but is accompanied by improved recovery of known causal genes in a controlled simulation setting.

*“****Simulation Framework for Empirical Power Evaluation***

*To complement the perturbation-based stability analysis and empirical Type I error evaluation, we designed a controlled power simulation with predefined causal genes. The simulation was intended to mimic a pathway-mediated disease architecture, where multiple genes within the same coordinated regulatory program jointly contribute to phenotypic variation. In each replicate, causal genes were planted within pathway-level expression structures, and phenotypes were generated under additive, hybrid, and interaction models across multiple heritability levels, allowing us to evaluate the ability of IBAS and competing methods to recover known causal genes under controlled genetic architectures. As in the main IBAS analysis, GTEx expression data were used as the reference transcriptome to derive pathway-level PCA components and estimate SNP weights, while WTCCC genotype data were used as the target dataset from which simulated phenotypes were generated.*

*Specifically, in each simulation replicate, one pathway was randomly selected, and the genes within that pathway were ranked according to the absolute values of their PC1 loadings derived from the reference expression data. Because PC1 captures the dominant coordinated expression pattern within a pathway, genes with large absolute loadings represent major contributors to this pathway-level regulatory structure. We therefore randomly selected 10 causal genes from the top 50 ranked genes, mimicking a setting in which disease risk is driven by genetic perturbations of genes central to a shared pathway program. Then for each causal gene, five cis-variants were randomly selected. When two genes were assigned as an interaction pair, their selected SNPs were matched one-to-one to form five SNP-pair interaction terms.*

*For each model, a genotype-derived genetic component* $g$ *was first constructed from the selected causal variants and then combined with environmental noise to achieve the target heritability. Given a prespecified* $h^{2}$*, we generated*

$$y=g+\epsilon,$$

*where* $\epsilon\sim N\left( 0,\sigma_{\epsilon}^{2}I \right)$*, and* $\sigma_{\epsilon}^{2}$ *was chosen such that*

$$h^{2}=\frac{Var\left( g \right)}{Var\left( g \right)+Var\left( \epsilon\right)}.$$

*We considered four heritability levels:* $h^{2}$ *= 0.05, 0.1, 0.4, and 0.7.*

*To evaluate method performance across different levels of interaction involvement, we used three phenotype-generating architectures. The additive model served as a baseline setting in which phenotypic variation was driven by marginal SNP effects, the interaction model represented an interaction-enriched setting driven by gene-gene interaction terms, and the hybrid model provided an intermediate architecture containing both marginal and interaction-mediated effects.*

*In the additive model, phenotypic variation was driven by marginal SNP effects across all 10 causal genes:*

$$g_{add}=\sum_{j=1}^{10} \sum_{k=1}^{5} \beta_{jk}x_{jk},$$

*where* $x_{jk}$ *is the genotype vector of the* $k$*-th selected SNP in causal gene* $j$*, and* $\beta_{jk}\sim N\left( 0,1 \right)$ *is the corresponding additive effect size.*

*In the hybrid model, four causal genes contributed additive effects, while the remaining six causal genes were assigned into three interaction pairs,*

$$g_{hyb}=\sum_{j=1}^{4} \sum_{k=1}^{5} \beta_{jk}x_{jk}+\sum_{\left( a,b \right)\in\mathcal{P}_{hyb}} \sum_{k=1}^{5} \gamma_{abk}\left( x_{ak}\odot x_{bk} \right).$$

*Where* $\mathcal{P}_{hyb}=\left( G_{5},G_{6} \right),\left( G_{7},G_{8} \right),\left( G_{9},G_{10} \right)$*.
In the interaction model, the 10 causal genes were assigned into five gene pairs,* $\mathcal{P}_{int}=\left( G_{1},G_{2} \right),\left( G_{3},G_{4} \right),\left( G_{5},G_{6} \right),\left( G_{7},G_{8} \right),\left( G_{9},G_{10} \right)$*, and phenotypic variation was generated only from cross-gene SNP interaction effects:*

$$g_{int}=\sum_{\left( a,b \right)\in\mathcal{P}_{int}} \sum_{k=1}^{5} \gamma_{abk}\left( x_{ak}\odot x_{bk} \right),$$

*where* $\odot$ *denotes elementwise multiplication across individuals, and* $\gamma_{abk}$ *is the interaction effect size.*

*For each combination of genetic architecture and heritability level, 500 simulation replicates were generated. We compared IBAS using PCA-derived weights with SKAT-O, PrediXcan, and MAGMA. For each method, gene-level p-values were adjusted using the Benjamini–Hochberg procedure at FDR 0.05, and empirical power was defined as the proportion of planted causal genes declared significant after correction.*

$$Power=\frac{\left| \hat{G}_{sig} \right|}{\left| G_{causal} \right|},$$

*where* $G_{causal}$ *is the set of planted causal genes and* $\hat{G}_{sig}$ *is the set of causal genes significant after BH correction.”*

*“****IBAS improves empirical power in simulation studies***

*To further evaluate the performance of IBAS, we assessed empirical power in simulations with predefined causal genes. This analysis tested whether the pathway-level interaction bridge used by IBAS improves the recovery of true genetic signals across different genetic architectures. By comparing additive, hybrid, and interaction architectures, the simulation further assessed whether the advantage of IBAS depends on the extent to which phenotypic variation is mediated through coordinated genetic effects within pathways. Across all three simulation models, IBAS using PCA-derived weights showed stronger recovery of causal genes than SKAT-O, PrediXcan, and MAGMA (****Fig. 5****). IBAS achieved comparable or higher power under the additive model, with increasingly clear advantages under the hybrid and interaction models. These results suggest that the pathway-level interaction representations learned by IBAS provide informative genetic prioritization beyond marginal gene effects or unweighted variant aggregation, enabling more effective recovery of coordinated genetic signals.*

*Although this simulation provides a simplified representation of complex disease biology, it offers a controlled setting for evaluating the recovery of known causal genes. The higher empirical power of IBAS suggests that pathway-informed interaction weights can improve sensitivity beyond conventional gene-based methods, while the real-data analyses further support its practical utility in identifying biologically meaningful genes and pathways across complex diseases.”*

*“****Figure 5 – Empirical power evaluation using planted causal gene simulations.***

*Empirical power was evaluated as the proportion of predefined causal genes recovered after Benjamini–Hochberg correction across 500 simulation replicates for each heritability level. IBAS using PCA-derived weights was compared with SKAT-O, PrediXcan, and MAGMA under three phenotype-generating models:* ***A)*** *additive model, where causal variants contribute through marginal SNP effects;* ***B)*** *hybrid model, where both additive and cross-gene interaction effects contribute to the phenotype; and* ***C)*** *interaction-enriched model, where phenotypic variation is driven by cross-gene product terms designed to mimic pathway-level dependency effects. Across the three settings, IBAS showed consistently strong recovery of causal genes, with more pronounced advantages under interaction-enriched architectures.”*

Minor follow-up: A small sensitivity check on the number of retained PCs (e.g., K ∈ {5, 10, 20} on one cohort) would help substantiate the Discussion's claim that IBAS is "robust to reasonable choices of dimensionality", since all current results use K = 10.

We thank the reviewer for this helpful suggestion. To address this point, we added a sensitivity analysis evaluating the effect of the number of retained principal components on the WTCCC T1D cohort. Specifically, we repeated the IBAS analysis using K = 5, 10, and 20 pathway components and compared the resulting top-ranked genes.

The results showed that, although the exact rankings varied across settings, the strongest biological signals remained stable. Several well-established T1D-associated genes, particularly within the MHC region, were consistently prioritized across different choices of K and were also supported by the independent dbGaP T1D cohort.

We added these results as a new Results section, “**Sensitivity to the number of principal components**” (Page 16, Line 701). This analysis supports the conclusion that the strongest disease-relevant signals, particularly MHC-region T1D genes, are stable across reasonable choices of K, whereas the exact ranking and composition of lower-priority genes remain sensitive to dimensionality.

***Sensitivity to the number of principal components***

*To evaluate the sensitivity of IBAS to the choice of dimensionality, we repeated the WTCCC T1D analysis using K = 5, 10, and 20 retained pathway components. As expected, the exact composition of the top-ranked genes varied across settings, reflecting the distributed covariance structure within biological pathways. The overlap between the top 50 genes identified using K = 10 and K = 20 was 23 genes (46%), and 14 genes were consistently identified across all three analyses. Importantly, these shared genes included several well-established T1D-associated genes within the major histocompatibility complex (MHC) region, including AGER, C2, HLA-B, HLA-DRA, LTA, TNF, and TNXB. Many of these genes were also prioritized in the independent dbGaP T1D cohort, indicating that the principal biological signals recovered by IBAS are reproducible across datasets and are not driven by a particular choice of dimensionality. These results suggest that the strongest disease-relevant signals, particularly MHC-region T1D genes, are stable across reasonable choices of K, whereas the exact ranking and composition of lower-priority genes remain sensitive to dimensionality. Notably, the MHC region represents the most robust and extensively validated genetic locus for T1D, and the persistence of multiple MHC genes across all K values and across independent cohorts supports the stability of the major biological conclusions derived from IBAS.”*

**Reviewer #3:** The authors have done an outstanding job revising their paper. All my concerns have been addressed. Great job.

We sincerely thank the reviewer for the very positive evaluation of our revised manuscript. We are grateful that the reviewer found that all concerns have been addressed, and we appreciate the encouraging comments!
