# Supplementary material for: IBAS: Interaction-bridged association studies discovering novel genes underlying complex traits
Source: PLoS Comput Biol. 2026 Aug 17;22(8):e1014640. doi: 10.1371/journal.pcbi.1014640 (PMC13480648; doi:10.1371/journal.pcbi.1014640)
Supplement: Peer review history

# Peer review: Decision Letter 0

**DOI:** 10.1371/journal.pcbi.1014640.r001

**Authors:** Ilya Ioshikhes, Can Yang

11 Dec 2025

IBAS: Interaction-bridged association studies discovering novel genes underlying complex traits

PLOS Computational Biology

Dear Dr. Wang,

Thank you for submitting your manuscript to PLOS Computational Biology. After careful consideration, we feel that it has merit but does not fully meet PLOS Computational Biology's publication criteria as it currently stands. Therefore, we invite you to submit a revised version of the manuscript that addresses the points raised during the review process.

Please submit your revised manuscript by Feb 10 2026 11:59PM. If you will need more time than this to complete your revisions, please reply to this message or contact the journal office at ploscompbiol@plos.org. When you're ready to submit your revision, log on to https://www.editorialmanager.com/pcompbiol/ and select the 'Submissions Needing Revision' folder to locate your manuscript file.

Please include the following items when submitting your revised manuscript:

* A rebuttal letter that responds to each point raised by the editor and reviewer(s). You should upload this letter as a separate file labeled 'Response to Reviewers'. This file does not need to include responses to formatting updates and technical items listed in the 'Journal Requirements' section below.

* A marked-up copy of your manuscript that highlights changes made to the original version. You should upload this as a separate file labeled 'Revised Manuscript with Track Changes'.

* An unmarked version of your revised paper without tracked changes. You should upload this as a separate file labeled 'Manuscript'.

If you would like to make changes to your financial disclosure, competing interests statement, or data availability statement, please make these updates within the submission form at the time of resubmission. Guidelines for resubmitting your figure files are available below the reviewer comments at the end of this letter

We look forward to receiving your revised manuscript.

Kind regards,

Can Yang

Section Editor

PLOS Computational Biology

Ilya Ioshikhes

Section Editor

PLOS Computational Biology

**Additional Editor Comments:**

Three experts have reviewed your manuscript, and they are positive on your proposed method in general. However, they still have some major comments to improve the current version of your manuscript. Please address them in a point-to-point manner in the revision.

**Journal Requirements:**

If the reviewer comments include a recommendation to cite specific previously published works, please review and evaluate these publications to determine whether they are relevant and should be cited. There is no requirement to cite these works unless the editor has indicated otherwise.

1) Please ensure that the CRediT author contributions listed for every co-author are completed accurately and in full.

At this stage, the following Authors/Authors require contributions: Pathum Kossinna, Dinghao Wang, Senitha Kumarapeli, and Qingrun Zhang. Please ensure that the full contributions of each author are acknowledged in the "Add/Edit/Remove Authors" section of our submission form.

The list of CRediT author contributions may be found here: https://journals.plos.org/ploscompbiol/s/authorship#loc-author-contributions

2) We ask that a manuscript source file is provided at Revision. Please upload your manuscript file as a .doc, .docx, .rtf or .tex. If you are providing a .tex file, please upload it under the item type u2018LaTeX Source Fileu2019 and leave your .pdf version as the item type u2018Manuscriptu2019.

3) Please provide an Author Summary. This should appear in your manuscript between the Abstract (if applicable) and the Introduction, and should be 150-200 words long. The aim should be to make your findings accessible to a wide audience that includes both scientists and non-scientists. Sample summaries can be found on our website under Submission Guidelines:

https://journals.plos.org/ploscompbiol/s/submission-guidelines#loc-parts-of-a-submission

4) Please upload all main figures as separate Figure files in .tif or .eps format. For more information about how to convert and format your figure files please see our guidelines:

https://journals.plos.org/ploscompbiol/s/figures

**Reviewers' comments:**

Reviewer's Responses to Questions

**Comments to the Authors:**

**Please note here if the review is uploaded as an attachment.**

Reviewer #1: Aiming to account for higher-order interactions of genes, the authors proposed IBAS (Interaction-Bridged Association Study), “a general framework that incorporates interaction patterns into association mapping” as a new TWAS method. Numerical comparisons with some existing methods and application to WTCCC GWAS data showed some advantages of the proposed method. The proposed method has potential to be useful in practice.

Main comments.

1. As stated in the abstract, the authors proposed “a general framework that incorporates interaction patterns into association mapping”. More specifically, some components, e.g. PCs, of the expression levels of a set of functionally related genes (taken form GO or KEGG etc) are associated with SNPs; the association parameter estimates are used as weights to form a kernel to test the association between the SNPs and an outcome. My main question is: would the authors show both theoretically and empirically how “interaction patterns” of the genes are in indeed incorporated into the proposed analysis (though for example use of PCs)?

2. The authors' view of TWAS, as demonstrated in a series of their publications, is to use gene expression data to select or weight SNPs, then conduct SNPs-outcome association testing (e.g. by a kernel method). This view is interesting and not wrong, but it is indeed different from the purpose of the mainstream TWAS: either for detecting causal genes to the outcome (in the framework of instrument variable regression) or for associating the genetically regulated gene expression (GReX) with the outcome. It would help the reader if the authors can state this difference clearly.

3. Since the old and much smaller WTCCC GWAS data were used in the application while the authors claim that their proposed IBAS is more powerful, could the authors use the results from some new and larger GWAS to demonstrate that indeed some of their unique discoveries are likely true positives?

Minor comments: Some notations or descriptions are not clear. Here are a few examples:

1. Is the same y used in eq (1) to represent an “interaction component in IBAS” and the outcome trait in eq (2)?

2. Eq (2): should y be centered at its sample mean (or generally its mean under H0)?

3. Section “Other Methods compared to IBAS”: are PrediXcan and kTWAS applied to a set of genes (as IBAS), instead of one gene? If so, how? Please write down your models explicitly.

Reviewer #2: This manuscript introduces the IBAS (Interaction-Bridged Association Study) method as an improvement over traditional TWAS, emphasizing the incorporation of gene interaction patterns into genotype–phenotype association analysis. The overall concept is innovative, but the methodological details, simulation design, and comparative experiments require further refinement. Below are my specific suggestions:

Major Comments

1. In the Methods section, the authors present several formulas (e.g., X, y, W) but do not specify the dimensionality of each matrix. This omission makes it difficult for readers to follow the correspondence between sample size, gene number, and SNP number. I recommend explicitly annotating dimensions in the formulas to improve clarity.

2. In IBAS, each pathway generates a set of SNP weights, but the manuscript does not clearly specify how these weights are integrated into a meta-weight. I recommend presenting explicit formulas for different strategies, such as coefficient-based averaging or p-value based Fisher combination, and evaluating their performance in simulations or real data.

3. The current simulation only perturbs expression matrices under GTEx genotypes but lacks validation of statistical properties. I recommend adding analyses such as:

Checking whether null p-values follow a Uniform[0,1] distribution.

Plotting ROC curves to compare sensitivity and specificity of IBAS vs other methods.

Computing type I error rates to ensure proper error control under the null.

4. The manuscript only compares IBAS with PrediXcan, which is insufficient. I recommend including additional benchmarks such as:

S-PrediXcan / MetaXcan: summary-statistics-based TWAS methods.

FUSION: another summary-level TWAS framework.

MAGMA: gene-set analysis method independent of eQTL.

SKAT-O: kernel-based gene-level association test.

5. In the dimensionality reduction of pathway expression matrices, the manuscript applies PCA but does not specify how many principal components are retained. The authors should provide a clear criterion for PCA dimensionality selection (e.g., cumulative variance explained ≥ 80% or a fixed number of top components) and evaluate the impact of different choices on results. Moreover, the manuscript also mentions alternative methods such as UMAP and t-SNE, which do not rely on variance explained. Therefore, it is even more important to clarify parameter selection strategies (e.g., number of neighbors and minimum distance for UMAP, perplexity for t-SNE) and assess robustness under different parameter settings. This would improve methodological transparency and reproducibility.

6. The manuscript consistently uses blood eQTL across all diseases, but certain diseases (e.g., Crohn’s disease, Rheumatoid arthritis, Type 1 diabetes) may require tissue-specific eQTL (e.g., gut, synovium, pancreas). The authors should justify tissue choice and, where possible, incorporate GTEx tissue-specific data.

Minor Comments

1. The authors emphasize the importance of gene interaction patterns in disease mechanisms, but the manuscript does not provide biological interpretation of the dimensionality reduction results. For example, PCA loadings can reveal which genes contribute most to interaction components. I recommend presenting gene loadings in supplementary materials and linking them to known pathway functions.

2. In Figure 4, the overlap of significant genes across methods is very limited. The authors should explain this phenomenon. I suspect it is due to the relatively small genotype sample size (GTEx ~1000 individuals, WTCCC ~5000 samples per disease). Larger datasets such as UK Biobank (>400,000 individuals) should be tested to validate robustness.

3. The manuscript does not report the computational complexity of IBAS. I recommend that the authors provide both a time complexity analysis and actual runtime results, and compare them with other methods such as TWAS and MAGMA.

Reviewer #3: In this paper, Kossina et al. developed IBAS (Interaction-Bridged Association Study), a general framework that incorporates interaction patterns into association mapping without exhaustive testing. IBAS employs statistical learning to derive low-dimensional representations of pathway-level interactions from transcriptomic data, which then serve as mediators in genotype–phenotype association tests. Overall the idea is interesting and reasonable. I have the following questions.

The authors kept saying that their IBAS method is able to capture interaction effects within the pathway. I don’t understand why they are saying this. Is interaction indicate gene-gene interaction? If so, why and how? The intermediate phenotypes, also known as endophenotypes in literature, are principal components (PCs) if using PCA, or some transformed gene expression measures if using t-SNE or UMAP. I don’t think these phenotypes can be interpreted as interactions. The authors need to justify their claim using specific example (can be artificial example) or drop the claim if they are unable to do so.

I am able to follow the method description up to the point that a gene can be associated with a principal component for the gene expression matrix for a pathway. But from that point, how to connect to a phenotype is confusing. Since there are thousands of pathways. For each gene, there will be hundreds or thousands of association signals, one for each pathway. Not mentioning multiple “interaction components” within each pathway. The authors need to describe how to integrate association signals to calculate association with a phenotype.

I am also confused about what is the basic element for association in IBAS. For GWAS, association is calculated at the SNP level. For TWAS, association is calculated at the gene level. I wonder what about IBAS? Can you show a Manhattan plot for IBAS? This will clarify lots of confusion.

Related to the above points, how do you comparison performance between TWAS and IBAS?

I do not quite get the points the authors are trying to make in Figures 2,3 and 4. How to connect associational signals of “interaction components” to genes?

I felt that the Discussion section was not fully developed. The pros and cons of the IBAS method should be discussed at least.

Lines 277-278, “Since IBAS works best if there is a high degree of overlap between the GWAS and reference datasets when considering genotypes”. I do not understand what does “overlap” means here?

Line 271, it says GWAS “contains samples from 670 individuals”. But in line 276, “Genotype data for 838 individuals and 43,066,422 SNPs were present in the raw VCF data.” This is contradictory.

**********

**Have the authors made all data and (if applicable) computational code underlying the findings in their manuscript fully available?**

The PLOS Data policy requires authors to make all data and code underlying the findings described in their manuscript fully available without restriction, with rare exception (please refer to the Data Availability Statement in the manuscript PDF file). The data and code should be provided as part of the manuscript or its supporting information, or deposited to a public repository. For example, in addition to summary statistics, the data points behind means, medians and variance measures should be available. If there are restrictions on publicly sharing data or code —e.g. participant privacy or use of data from a third party—those must be specified.

Reviewer #1: None

Reviewer #2: Yes

Reviewer #3: Yes

**********

PLOS authors have the option to publish the peer review history of their article (what does this mean?). If published, this will include your full peer review and any attached files.

If you choose “no”, your identity will remain anonymous but your review may still be made public.

**Do you want your identity to be public for this peer review?**  For information about this choice, including consent withdrawal, please see our Privacy Policy.

Reviewer #1: No

Reviewer #2: No

Reviewer #3: No

**Figure resubmission:**

**Reproducibility:**

?>

# Author comment: Author response to Decision Letter 1

**DOI:** 10.1371/journal.pcbi.1014640.r002

19 Apr 2026

Attachment Submitted filename: IBAS Revision PLoS CB_v3.docx

# Peer review: Decision Letter 1

**DOI:** 10.1371/journal.pcbi.1014640.r003

**Authors:** Ilya Ioshikhes, Can Yang

2 Jun 2026

PCOMPBIOL-D-25-02242R1

IBAS: Interaction-bridged association studies discovering novel genes underlying complex traits

PLOS Computational Biology

Dear Dr. Zhang,

Thank you for submitting your manuscript to PLOS Computational Biology. After careful consideration, we feel that it has merit but does not fully meet PLOS Computational Biology's publication criteria as it currently stands. Therefore, we invite you to submit a revised version of the manuscript that addresses the points raised during the review process.

Please submit your revised manuscript by Aug 02 2026 11:59PM. If you will need more time than this to complete your revisions, please reply to this message or contact the journal office at ploscompbiol@plos.org. When you're ready to submit your revision, log on to https://www.editorialmanager.com/pcompbiol/ and select the 'Submissions Needing Revision' folder to locate your manuscript file.

Please include the following items when submitting your revised manuscript:

* A letter that responds to each point raised by the editor and reviewer(s). You should upload this letter as a separate file labeled 'Response to Reviewers'. This file does not need to include responses to formatting updates and technical items listed in the 'Journal Requirements' section below.

* A marked-up copy of your manuscript that highlights changes made to the original version. You should upload this as a separate file labeled 'Revised Manuscript with Track Changes'.

* An unmarked version of your revised paper without tracked changes. You should upload this as a separate file labeled 'Manuscript'.

If you would like to make changes to your financial disclosure, competing interests statement, or data availability statement, please make these updates within the submission form at the time of resubmission. Guidelines for resubmitting your figure files are available below the reviewer comments at the end of this letter.

As the corresponding author, your ORCID iD is verified in the submission system and will appear in the published article. PLOS supports the use of ORCID, and we encourage all coauthors to register for an ORCID iD and use it as well. Please encourage your coauthors to verify their ORCID iD within the submission system before final acceptance, as unverified ORCID iDs will not appear in the published article. Only the individual author can complete the verification step; PLOS staff cannot verify ORCID iDs on behalf of authors.

We look forward to receiving your revised manuscript.

Kind regards,

Can Yang

Section Editor

PLOS Computational Biology

Ilya Ioshikhes

Section Editor

PLOS Computational Biology

Note: If the reviewer comments include a recommendation to cite specific previously published works, please review and evaluate these publications to determine whether they are relevant and should be cited. There is no requirement to cite these works unless the editor has indicated otherwise.

**Reviewers' comments:**

Reviewer's Responses to Questions

Reviewer #1: This is one of the most responsive revisions I have seen! A huge “thank you” to the authors! I am satisfied and pleased with the revision!

I also just realize that what the authors mean by “gene-gene interaction” is coordinated or co-regulated gene expression, or simply co-expression, instead of typical statistical “interaction” or genetic “epistasis” that I thought it meant; if so, then why dimension reduction techniques like PCA work is more obvious; that’s also why “meta-genes” or “modules” are used in other related techniques/papers. I don’t want to cause more troubles, but feel that if it is possible that the authors can clarify this at the beginning, it would make it much easier to understand; I leave it to the authors to decide.

Reviewer #2: I thank the authors for the careful revision. The new Supplementary Note S1, the Type I error analysis, the tissue-specific analyses, the dbGaP replication, and the additional benchmarks together represent a clear improvement, and most of my original concerns have been addressed. Two small points remain.

1. Supplementary Note S1 elegantly shows that PCA recovers block-correlated covariance, i.e., co-expression structure. This is distinct from gene–gene interaction in the statistical-genetics sense. I suggest either softening the terminology throughout to "coordinated activity" or "co-expression structure", or acknowledging this scope in the Introduction.

2. Fig. 3 demonstrates stability under perturbation, but a method that detects nothing would also be perfectly stable. Could the authors add a simulation in which a known set of causal genes is planted within a pathway, and report the empirical power (proportion of these recovered) for IBAS vs. competitors at a fixed significance level?

Minor follow-up: A small sensitivity check on the number of retained PCs (e.g., K ∈ {5, 10, 20} on one cohort) would help substantiate the Discussion's claim that IBAS is "robust to reasonable choices of dimensionality", since all current results use K = 10.

Reviewer #3: The authors have done an outstanding job revising their paper. All my concerns have been addressed. Great job.

**********

**Have the authors made all data and (if applicable) computational code underlying the findings in their manuscript fully available?**

The PLOS Data policy requires authors to make all data and code underlying the findings described in their manuscript fully available without restriction, with rare exception (please refer to the Data Availability Statement in the manuscript PDF file). The data and code should be provided as part of the manuscript or its supporting information, or deposited to a public repository. For example, in addition to summary statistics, the data points behind means, medians and variance measures should be available. If there are restrictions on publicly sharing data or code —e.g. participant privacy or use of data from a third party—those must be specified.

Reviewer #1: None

Reviewer #2: Yes

Reviewer #3: Yes

**********

PLOS authors have the option to publish the peer review history of their article (what does this mean?). If published, this will include your full peer review and any attached files.

If you choose “no”, your identity will remain anonymous but your review may still be made public.

**Do you want your identity to be public for this peer review?**  For information about this choice, including consent withdrawal, please see our Privacy Policy.

Reviewer #1: No

Reviewer #2: No

Reviewer #3: **Yes:**  Zhaohui Qin

[NOTE: If reviewer comments were submitted as an attachment file, they will be attached to this email and accessible via the submission site. Please log into your account, locate the manuscript record, and check for the action link "View Attachments". If this link does not appear, there are no attachment files.]

**Figure resubmission:**

**Reproducibility:**

To enhance the reproducibility of your results, we recommend that authors of applicable studies deposit laboratory protocols in protocols.io, where a protocol can be assigned its own identifier (DOI) such that it can be cited independently in the future. Additionally, PLOS ONE offers an option to publish peer-reviewed clinical study protocols. Read more information on sharing protocols at https://plos.org/protocols?utm_medium=editorial-email&utm_source=authorletters&utm_campaign=protocols

# Author comment: Author response to Decision Letter 2

**DOI:** 10.1371/journal.pcbi.1014640.r004

14 Jul 2026

Attachment Submitted filename: Second_Revision_Response_PLoS_CB.docx

# Peer review: Decision Letter 2

**DOI:** 10.1371/journal.pcbi.1014640.r005

**Authors:** Ilya Ioshikhes

29 Jul 2026

Dear Dr Zhang,

We are pleased to inform you that your manuscript 'IBAS: Interaction-bridged association studies discovering novel genes underlying complex traits' has been provisionally accepted for publication in PLOS Computational Biology.

Before your manuscript can be formally accepted you will need to complete some formatting changes, which you will receive in a follow up email. A member of our team will be in touch with a set of requests.

Please note that your manuscript will not be scheduled for publication until you have made the required changes, so a swift response is appreciated.

IMPORTANT: The editorial review process is now complete. PLOS will only permit corrections to spelling, formatting or significant scientific errors from this point onwards. Requests for major changes, or any which affect the scientific understanding of your work, will cause delays to the publication date of your manuscript.

Should you, your institution's press office or the journal office choose to press release your paper, you will automatically be opted out of early publication. We ask that you notify us now if you or your institution is planning to press release the article. All press must be co-ordinated with PLOS.

Thank you again for supporting Open Access publishing; we are looking forward to publishing your work in PLOS Computational Biology.

Best regards,

Ilya Ioshikhes

Section Editor

PLOS Computational Biology

Ilya Ioshikhes

Section Editor

PLOS Computational Biology

***********************************************************

Reviewer's Responses to Questions

**Comments to the Authors:**

**Please note here if the review is uploaded as an attachment.**

Reviewer #2: I thank the authors for the careful and complete response. I have no further concerns.

**********

**Have the authors made all data and (if applicable) computational code underlying the findings in their manuscript fully available?**

The PLOS Data policy requires authors to make all data and code underlying the findings described in their manuscript fully available without restriction, with rare exception (please refer to the Data Availability Statement in the manuscript PDF file). The data and code should be provided as part of the manuscript or its supporting information, or deposited to a public repository. For example, in addition to summary statistics, the data points behind means, medians and variance measures should be available. If there are restrictions on publicly sharing data or code —e.g. participant privacy or use of data from a third party—those must be specified.

Reviewer #2: Yes

**********

PLOS authors have the option to publish the peer review history of their article (what does this mean?). If published, this will include your full peer review and any attached files.

If you choose “no”, your identity will remain anonymous but your review may still be made public.

**Do you want your identity to be public for this peer review?**  For information about this choice, including consent withdrawal, please see our Privacy Policy.

Reviewer #2: No

# Editor report: Acceptance letter

**DOI:** 10.1371/journal.pcbi.1014640.r006

**Authors:** Ilya Ioshikhes

PCOMPBIOL-D-25-02242R2

IBAS: Interaction-bridged association studies discovering novel genes underlying complex traits

Dear Dr Zhang,

I am pleased to inform you that your manuscript has been formally accepted for publication in PLOS Computational Biology. Your manuscript is now with our production department and you will be notified of the publication date in due course.

The corresponding author will soon be receiving a typeset proof for review, to ensure errors have not been introduced during production. Please review the PDF proof of your manuscript carefully, as this is the last chance to correct any errors. Please note that major changes, or those which affect the scientific understanding of the work, will likely cause delays to the publication date of your manuscript.

Soon after your final files are uploaded, unless you have opted out, the early version of your manuscript will be published online. The date of the early version will be your article's publication date. The final article will be published to the same URL, and all versions of the paper will be accessible to readers.

For Research, Software, and Methods articles, you will receive an invoice from PLOS for your publication fee after your manuscript has reached the completed accept phase. If you receive an email requesting payment before acceptance or for any other service, this may be a phishing scheme. Learn how to identify phishing emails and protect your accounts at https://explore.plos.org/phishing.

Thank you again for supporting PLOS Computational Biology and open-access publishing. We are looking forward to publishing your work!

With kind regards,

Sharmila Kamatchi

PLOS Computational Biology | Carlyle House, Carlyle Road, Cambridge CB4 3DN | United Kingdom ploscompbiol@plos.org | Phone +44 (0) 1223-442824 | ploscompbiol.org | @PLOSCompBiol
